# 5-(1*H*-Indol-3-ylmethylene)-4-oxo-2-thioxothiazolidin-3-yl)alkancarboxylic Acids as Antimicrobial Agents: Synthesis, Biological Evaluation, and Molecular Docking Studies

**DOI:** 10.3390/molecules25081964

**Published:** 2020-04-23

**Authors:** Volodymyr Horishny, Victor Kartsev, Athina Geronikaki, Vasyl Matiychuk, Anthi Petrou, Jasmina Glamoclija, Ana Ciric, Marina Sokovic

**Affiliations:** 1 Department of Chemistry, Danylo Halytsky Lviv National Medical University, Pekarska 69, 79010 Lviv, Ukraine; vgor58@ukr.net; 2 InterBioScreen, 85355 Moscow, Russia; vkartsev@ibscreen.chg.ru; 3 Department of Phram Chemistry, School of Pharmacy, Aristotle University of Thessaloniki, 54124 Thessaloniki, Greece; anthi.petrou.thessaloniki1@gmail.com; 4 Department of Chemistry, Ivan Franko National University of Lviv, Kyryla i Mefodia 6, 79005 Lviv, Ukraine; v_matiychuk@ukr.net; 5 Mycological Laboratory, Institute of Biological Research Sinisa Stankovic, National Institute of Republic of Serbia, Belgrade University, 11000 Belgrade, Serbia; jasna@ibiss.bg.ac.rs (J.G.); rancic@ibiss.bg.ac.rs (A.C.); mris@ibiss.bg.ac.rs (M.S.)

**Keywords:** indole, antimicrobial, antifungal, *E. coli* MurB, *Candida albicans* 14^α^-demethylase, CYP51, molecular docking

## Abstract

Background: Infectious diseases symbolize a global consequential strain on public health security and impact on the socio-economic stability all over the world. The increasing resistance to the current antimicrobial treatment has resulted in crucial need for the discovery and development of novel entity for the infectious treatment with different modes of action that could target both sensitive and resistant strains. Methods: Compounds were synthesized using classical methods of organic synthesis. Results: All 20 synthesized compounds showed antibacterial activity against eight Gram-positive and Gram-negative bacterial species. It should be mentioned that all compounds exhibited better antibacterial potency than ampicillin against all bacteria tested. Furthermore, 18 compounds appeared to be more potent than streptomycin against *Staphylococcus aureus, Enterobacter cloacae, Pseudomonas aeruginosa*, *Listeria monocytogenes,* and *Escherichia coli*. Three the most active compounds **4h**, **5b**, and **5g** appeared to be more potent against MRSA than ampicillin, while streptomycin did not show any bactericidal activity. All three compounds displayed better activity also against resistant strains *P. aeruginosa* and *E. coli* than ampicillin. Furthermore, all compounds were able to inhibit biofilm formation 2- to 4-times more than both reference drugs. Compounds were evaluated also for their antifungal activity against eight species. The evaluation revealed that all compounds exhibited antifungal activity better than the reference drugs bifonazole and ketoconazole. Molecular docking studies on antibacterial and antifungal targets were performed in order to elucidate the mechanism of antibacterial activity of synthesized compounds. Conclusion: All tested compounds showed good antibacterial and antifungal activity better than that of reference drugs and three the most active compounds could consider as lead compounds for the development of new more potent agents.

## 1. Introduction

Infectious diseases symbolize a global consequential strain on public health security and impact on the socio-economic stability all over the world [1]. For centuries they have monopolized the prevailing factors of death and disability of millions of humans and are presently plaguing and even ravaging populations worldwide each year, far surpassing the impact of wars. The growing challenges on health and human economic progresses posed by infectious diseases is further aggravated by the continuous emergence of new, obscure, and old endemic infections of global impact [1]. Indeed, during the past two decades, the world’s scientific community was besieged by tremendous concerns caused by infectious diseases whose incidence in humans has augmented for reasons such as the emergence of new pathogens and the development of antimicrobial resistance [2]. At least 30 new infections have risen insidiously and scattered to threaten the health of billions of humans across the planet especially in low-income countries. Unfortunately, for many of them, there is no effective treatment or vaccine alongside with limited scope of control or prevention strategies [3].

Despite the achievements in treatment of infective diseases during the last 50 years, unfortunately the new infections and diseases affecting large populations, are instigating significant morbidity and mortality, most recently as the syndrome of acquired immunodeficiency.

The increasing resistance to the current antimicrobial treatment has resulted in crucial need for the discovery and development of novel entity for the infectious treatment with different modes of action that could target both sensitive and resistant strains [4]. This need is even greater for patients suffering from chronic inflammatory bowel diseases. During an inflammatory response in the gut, some commensally microorganisms such as *Escherichia coli* and *Candida albicans* can thrive and contribute to illness [5].

One of the promising methods for solving the resistance problem is screening of potential antimicrobial agents among new classes of chemical compounds [6].

Rhodanine (2-thioxo-4-thiazolidone) derivatives during last years attracted the interest of scientists due to their wide range of biological activities mainly to control human immunodeficiency virus (HIV), hepatitis C virus (HCV), and dengue virus proteins [7].

5-Arylidene derivatives of rhodanines were found to possess various types of activity, in particular, antitumor [8], antiviral [7,9], anti-inflammatory, antidiabetic [10,11,12], antioxidant [13], LOX and cholinesterase inhibitory activities [14,15] as well as aldose reductase inhibitory activities inhibitory [16].

On the other hand, 5-arylidene-2-thioxo-4-thiazolidinones **1** are highly selective inhibitors of UDP-MurNAc/L-Ala ligase, which are characterized by the influence on gram-positive methicillin-resistant strains on the bacterial wall formation process of *Staphylococcus aureus* (MRSA) and are promising for in-depth studies [17]. A number of 5-benzylidene-2,4-thiazolidinediones **2** exhibit high effect against Gram-positive microorganisms (*Staphylococcus aureus, Enterococcus faecalis, Streptococcus pneumoniae*). These microorganism are among the six pathogens with growing multidrug resistance and virulence, named ESKAPE (*Enterococcus faecium, Staphylococcus aureus, Klebsiella pneumoniae, Acinetobacter baumannii, Pseudomonas aeruginosa,* and *Enterobacter* *spp*.) [18]. They are among recently listed ESKAPE pathogens in the list of 12 bacteria by World Health Organization (WHO) against which new antibiotics are urgently needed [19].

There are many references in the literature regarding antimicrobial activity of rhodanine derivatives [20,21,22,23,24,25]. In particular, the high potential of 5-arylidenerodanine-3-carboxylic acids was reported [4,5,7,8]. It could be noticed that, the rhodanine cycle is considered to be privileged [11,12,13,14].

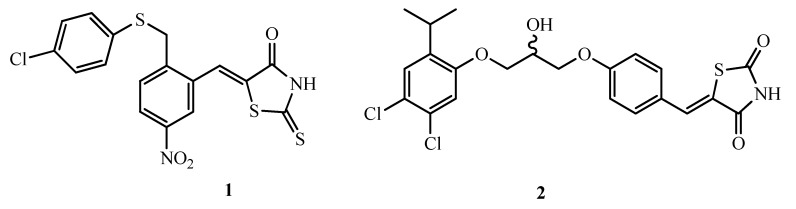



Another interesting class of heterocyclic compounds is 5-(1*H*-Indol-3-ylmethylene) -2-thioxothiazolidin-4-ones with wide spectrum of biological activities as well. Among them are antitumor [26,27] and antimicrobial [28,29], inhibitors of proteases anthrax lethal factor, inhibitors against neurotoxin type A [28], aldose reductase [30], PIM kinase [31], PI3Kα [32], IKKβ [33], and GSK-3 [34] enzymes.

Taking all mentioned above into account, herein we present the synthesis of new rodanin derivatives and evaluation their antimicrobial activity. as well as their effect on biofilm formation since it is one of the most considerable bacterial virulence factors. It was found that biofilm formation is involved in a wide range of microbial infections in the body and is responsible for the serious chronic diseases (80%) resistant to the most antibiotics used for therapy [35].

## 2. Results and Discussion

### 2.1. Chemistry

**Scheme 1:**
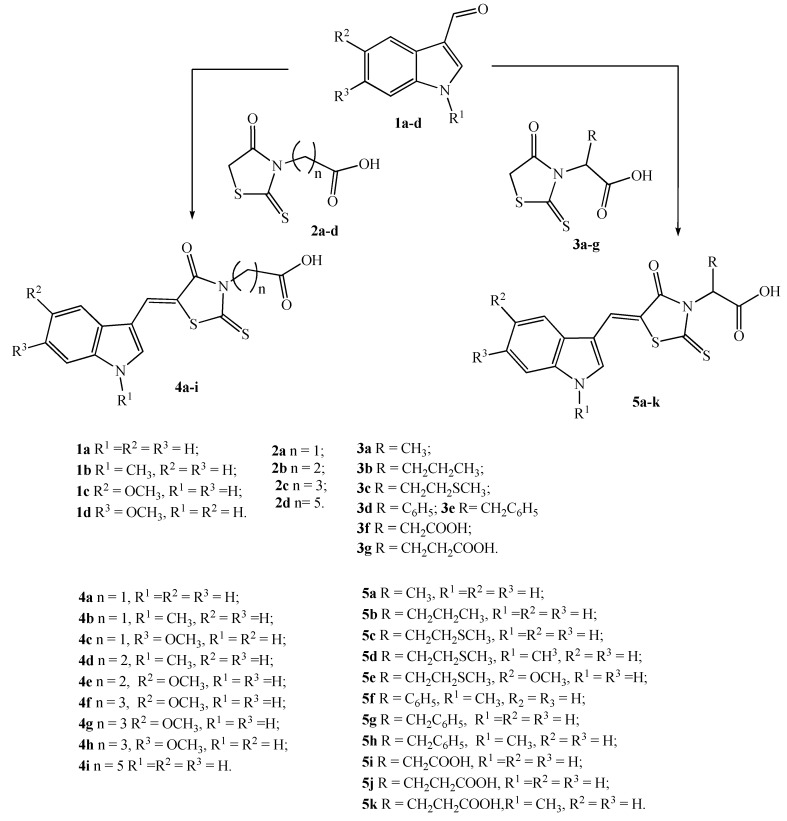
Synthesis of title compounds.

The title compounds were synthesized according to Scheme 1. As starting reagents for the synthesis of the target (*Z*)-[5-(1-R^1^,5-R^2^,6-R^3^-1*H*-indol-3-ylmethylene)-4-oxo-2-thioxothiazolidin -3-yl] alkanecarboxylic acids **4a**–**i** and **5a**–**k** the amino acids glycine, β-alanine, GABA, ε-aminocaproic acid and a number of α-amino acids such as l-alanine, d, l-norvaline, methionine, d, l-amino(phenyl)acetic acid, l-phenylalanine, d, l-aspartic and l-glutamic acid were used. Interaction of amino acids with carbon disulfide in alkaline medium converted them to dithiocarbamic salts. After alkylation of the latest with monochloracetic acid and subsequent cyclization, 4-oxo-2-thioxothiazolidin-3-ylalkanecarboxylic acids **2a**–**d** and **3a**–**g** were obtained.

At the final stage of the synthesis of the target products, compounds **2a**–**d** and **3a**–**g** underwent the condensation with indole-3-carbaldehydes (**1a**–**d**). The interaction was carried out in boiling alcohol in the presence of ammonium acetate. As a result, (*Z*)-[5-(1-R1,5-R^2^,6-R^3^-1*H*-indol-3-ylmethylene)-4-oxo-2-thioxothiazolidin-3-yl]alkanecarboxylic acids **4a**–**i** and **5a**–**k** were obtained.

The structure of all synthesized compounds was confirmed by NMR spectroscopy. In the 1 H NMR spectra, signals of all protons are observed in regions corresponding to the structure of the synthesized substances. In particular, the signals of the methylene group at position 5 of 4-oxo-2-thioxothiazolidin-3-ylalkanecarboxylic acids **2a**–**d** and **3a**–**g** are in the range 4.43–4.22 ppm. Signals of NH protons of the indole cycle are observed at 11.96–12.43 ppm. The signals of the protons of the methylidene group CH = appear as a singlet at 8.10–7.95 ppm, which indicates the *Z*-configuration of these compounds. Protons NCH_2_ of the group of compounds **2a**–**d** and **4a**–**i** resonate at 2.63–2.19 ppm. and protons of the CH_2_COOH group of the same compounds at 4.72–3.76 ppm. At the same time, protons of the NCH group in compounds **3a**–**g** and **5a**–**k** are observed in the range of 6.76–5.30 ppm.

### 2.2. Biological Evaluation

#### 2.2.1. Antibacterial Activity

Synthesized compounds were evaluated for their antibacterial activity using microdilution method for the determination of their minimal inhibitory and minimal bactericidal/fungicidal concentrations.

All compounds showed antibacterial activity and results are shown in Table 1.The antibacterial potential of these compounds can be presented as follows: **5b** > **4h** > **5g** > **5d** > **4g** > **5e** > **4b** > **5c** > **5k** > **5h** > **4e** > **4d** > **4i** > **4c** > **5f** > **4f** > **5a** > **4a** > **5i** > **5j**. The best antibacterial activity was observed for compound **19 5b** with MIC at 0.56–12.50 μM × 10^−2^ and MBC at 2.08–16.67 μM × 10^−2^, while compound **9 5j** was the less active with MIC and MBC at 7.68–30.74 μM × 10^−2^ and 15.37–61.48 μM × 10^−2^ respectively.

It was observed that bacteria in general showed different sensitivity towards compounds tested. Thus, the most sensitive bacterium appeared to be *S. aureus* followed by *P. aeruginosa*, while *L. monocytogenes* and *E. coli* were the most resistant representatives of Gram-positive and Gram-negative bacteria. The antibacterial potency of compounds against *S. aureus* can be presented as: **5b** > **5g** > **4h** > **4b** > **4i** > **5h** > **5d** > **5k** > **4g** > **5i** > **4e** > **4f** > **5a** > **5f** > **4a** > **5e** > **5c** > **5j** > **4c** > **4d**, while against *E. coli* as: **4g** > **5d** > **5c** > **4h** > **5b** > **4i** > **5k = 5e** > **5f** > **4f** > **4b** > **5g** > **4d** > **5h** > **5i** > **4e** > **4c** > **5a** > **4a** > **5j**. Compounds **5b** and **5g** exhibited very good activity against *Bacillus cereus, S. aureus, L. monocytogenes, En. cloacae,* and *S. typhimirium* with MIC at 0.56–4.17 μM × 10^−2^ and MBC at 2.08–3.68 μM × 10^−2^.Good activity was observed for compound **4h** against *M. flavus* and *S. aureus* with MIC and MBC at 1.99 μM × 10^−2^and 3.98μM × 10^−2^ respectively and against all other bacteria with MIC value at 3.69 μM × 10^−2^and MBC at 7.38 μM × 10^−2^. Compound **5d** showed the same good activity with compound **4h** with MIC and MBC value at 3.69 μM × 10^−2^and 7.38 μM × 10^−2^ respectively against all bacteria tested except of *L. monocytogenes* and *S typhimirium*.

It was observed that for Gram-positive bacteria the range of MIC and MBC was 0.56–30.74 μM × 10^−2^ and 3.68–61.48 μM × 10^−2 ^ respectively, while for Gram-negative bacteria this range was MIC at 1.67–28.5 μM × 10^−2^ and MBC at 3.68–37.72 μM × 10^−2^. It seems that Gram-negative bacteria appeared to be more sensitive to compounds tested than Gram-positive.

Finally, it should be mentioned that all compounds exhibited better antibacterial potency than ampicillin against all bacteria tested. Furthermore, all compounds appeared to be more potent than streptomycin against *S. aureus, En. cloacae, P. aeruginosa*, *L. monocytogenes* (except of compound **5j**), and *E. coli* (except of **5j** and **4a**). Compounds **4f**, **4h**, **4i**, and **5b**, **5d**, **5g**, and **5k** were more potent than streptomycin against *B. cereus* while compound **4b**, **4d**–**4i**, **5c**, **5d**, **5h**, **5i**, and **5k** exhibited better antibacterial potential than streptomycin against *M. flavus* (Table 1).

Ampicillin exhibited showed an inhibitory potential at 24.8–74.4 × 10^−2^ μM and bactericidal at 37.2–124.0 × 10^−2^ μM, while MIC/MBC of streptomycin is 4.3–25.8/8.6–34.4× 10^−2^ μM.

Three the most active compounds were tested against three resistant strains: Methicillin resistant *S. aureus, MRSA, P. aeruginosa and E. coli.* (Table 2) All compounds appeared to be more potent against MRSA, which is in the list of high priority group according to the urgency of need for new antibiotics, than ampicillin, while streptomycin did not show any bactericidal activity. All three compounds displayed better activity also against resistant strains *P. aeruginosa* and *E. coli* than ampicillin, which did not show any bactericidal effect. These compounds were tested also for their effect on biofilm formation. The evaluation revealed that all compounds were able to inhibit biofilm formation 2-to 4 times more than both reference drugs (Table 2). The best ability was achieved for compound **4h** followed by **5g** and the most active compound **5b**.It should be mentioned that two compounds (**5b, 5g**) displayed better effect on biofilm formation than reference drugs even in concentration of 0.5 MIC. The best effect was observed for compound **5g.**

A structure-activity relationship study revealed that the presence of pentanoic acid as substituent on the nitrogen of 2-thioxothiazolidin-4-one ring of 5-(1*H*-indol-3-ylmethylene) (**5b**) was beneficial for the antibacterial activity. Replacement of pentanoic acid by butyric and introduction of methoxy group in position 6 of indole ring gave the second most active compound (**4h**). The presence of 3-phenylpropanoic acid (**5g**) decreased a little activity. Introduction of 4-(methylthio) butanoic acid to the nitrogen of 2-thioxothiazolidin-4-one ring and at the same time methyl group to the nitrogen of 1H-indole resulted less active compound (**5d**) than compound **5g**, but still being among the most active compounds. On the other hand, replacement of methyl indole in compound **5d** by indole led to compound (**5c**) which is in the middle of activity order. The presence of butyric acid in 2-thioxothiazolidin-4-one moiety in combination with 5-OCH_3_ group in indole ring (**12 4g**) decrease more activity compared to **11 5d**. The replacement of butyric acid 2-methyl-4-(methylthio)butanoic acid of **11 5d** by 4-(methylthio)butanoic acid **(5e**) did not improved activity, decreasing it more. Finally, the presence of dicarboxylic glutaric acid (**5j**) as a substituent of (*Z*)-5-(1*H*-indol-3-ylmethylene)-2-thioxothiazolidin-4-one was detrimental for antibacterial activity.

From all mentioned above, it is obvious that the activity depends not only from the nature of substituents in 2-thioxothiazolidin-4-one moiety and indole ring but also from their position (compounds **4h** and **4e**). The presence of dicarboxylic acids is not beneficial for antibacterial activity, no for N-methylindole derivatives, no for indole derivatives. In this case the activity decreased in the following order: pentanoic (valeric) acid > 3-phenyl propanoic acid > propionic acid > acetic acid. In case of *N*-methylindole derivatives the activity decreased from acetic acid as substituent to butyric acid. In case of metoxy subastitution in positions 5 and 6 of the indole ring as longer is the chain of acids as better is activity.

#### 2.2.2. Antifungal Activity

Compounds were tested also against of panel of eight fungi, using ketoconazole and bifonazole as reference drugs. The antifungal potential of synthesized compounds is presented in Table 3 and the order of activity is: **5f** > **4d** > **4h** > **4g** > **5e** > **5i** > **4b** > **5j** > **4c** > **5g** > **5c** > **5k** > **5b** > **5a** > **5h** > **4e** > **4f** > **5d** > **4a** > **4i**. Compound **5f** appear to be the most potent, achieving inhibitory activity with MIC values ranging at 2.31–4.33 μM × 10^−2^ and MFC at 3.67–7.34 μM × 10^−2^, while compound **4i** showed the lowest antifungal activity with MIC and MFC at 4.00–32.04 μM × 10^−2^ and 8.01–64.09 μM × 10^−2^ respectively.

Ketoconazole showed antifungal potential at MIC 38.00–475.00 × 10^−2^ and MFC 57.00–570.00 × 10^−2^ respectively, while bifonazole showed MIC at 32.00–64.00 × 10^−2^ and MFC at 48.30–80.00 × 10^−2^ μM × 10^−2^ respectively. The obtained results revealed that all tested compounds exhibited higher antifungal activity than both drugs tested (Table 3).

The most sensitive fungal species is T*richoderma viride* whereas *Aaspergillus fumigatus* appeared to be the most resistant one (Table 3).

It should be mentioned that fungi, as in case of bacteria showed different sensitivity towards compounds tested. Thus, the sensitivity of *T. viride* can be presented as follows: **5e** > **4g** > **5c** > **5h** > **5b** > **5f** > **4c** > **4d** > **5j** > **4h** > **4e** > **4b** > **5g** > **5d** > **5k** > **5c** > **5i** > **4i** > **4f** > **5a** > **4a**, while of *A. fumigates* was: **4h** > **4d** > **4b** > **5f** > **5i** = **4g** > **5k** > **5e** > **5g** > **5b** > **5j** > **4c** > **5c** > **5h** > **5a** > **4a** > **5d** > **4i** > **4f** > **4e**. Nevertheless, despite different sensitivity of fungi towards compounds tested, all of them, except *A. fumigatus* appeared to be sensitive to compound **5e** and in most cases to compound **5f**. Compound **4h** showed very good activity against *A. fumigatus* with MIC at 1.98–3.98 μM × 10^−2^ and MFC at 3.98–7.97 μM × 10^−2^, followed by compounds **4d** and **4b** with MIC at 2.31–4.33 μM × 10^−2^ μM and 3.31–4.51 μM × 10^−2^ respectively and MFC at 4.33–8.66 μM × 10^−2^ and 4.51–9.03 μM × 10^−2^ respectively. Good activity was exhibited by compound **4g** against *T. viride* with MIC at 2.12–3.98 μM × 10^−2^ and MFC at 3.96-7.97 μM × 10^−2^.

From the study of structure-activity relationships it is obvious that the presence of phenylacetic acid as substituent in (*Z*)-5-(1-methyl-1*H*-indol-3-ylmethylene)-2-thioxothiazolidin-4-one (**5f**) is the most beneficial for antifungal activity. Replacement of phenylacetic acid by propionic acid gave a little less active compound (**4d**), while introduction of butyric acid (**4h**) decreased more the antifungal activity. In general, for (*Z*)-5-(1-methyl-1*H*-indol-3-ylmethylene)-2-thioxothiazolidin-4-one derivatives the substituent such as 4-(methylthio) butanoic acid and butyric acid had negative effect on antifungal activity followed by 3-phenyl propanoic acid. On the other hand, for not beneficial for activity for (*Z*)-5-(1*H*-indol-3-ylmethylene)-2- thioxothiazolidin-4-one derivatives appeared to be the 5-methoxy propanoic acid, acetic and hexanoic acid. The presence of the last one, as already mentioned, was very negative for antifungal activity.

It should be mentioned, that as in case of antibacterial activity, antifungal activity depends not only on the nature of substituent in 2-thioxothiazolidin-4-one moiety but also on its nature and position in indole ring. Thus, the replacement of acetic acid substituent in (*Z*)-5-(5-methoxy-1*H*-indol-3-ylmethylene)-2-thioxothiazolidin-4-one (**4c**) by propanoic acid remarkably decreased the activity. On the other hand, shifting the methoxy group from position 5 of indole ring of (*Z*)-4-[5-(5-methoxy-1H-indol-3-ylmethylene)-4-oxo-2-thioxothiazolidin-3-yl)butanoic acid to position 6 led to small increase in activity.

### 2.3. Docking Studies

In order to elucidate the probable mechanism of antibacterial and antifungal activity of tested compounds docking studies were performed on three bacterial targets; DNA Gyrase, Thymidylate kinase and *E. coli* MurB enzymes. Compounds were also docked to lanosterol 14a-demethylase of *C. albicans* for antifungal activity mechanism.

#### 2.3.1. Docking to Antibacterial Targets

The docking studies revealed that Free Energy of Binding to DNA Gyrase (−1.28–−7.15kcal/mol) as well as to Thymidylate kinase (−2.26–−4.66 kcal/mol), were higher than that to *E. coli* MurB (−5.73–−12.33), therefore it may be resolved that *E. coli* MurB is the most suitable enzyme where binding scores were consistent with biological activity (Table 4).

**Figure 1:**
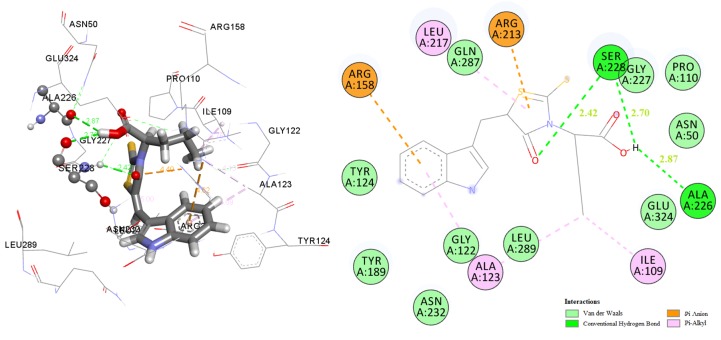
Docked conformation of the most active compound **5b** in *E. coli* MurB.

The docking pose of the most active compound **5b** in *E. coli* MurB enzyme showed three favorable hydrogen bond interactions. The first one between the hydrogen atom of OH group of the compound and the oxygen of the side chain of Ser228, the second between the oxygen atom of the C=O group of the compound and the side chain of Ser228 (distance 2.70 Å and 2.42 Å respectively), and the last one hydrogen bond between the hydrogen atom of OH group of the compound and side chain of Ala226 (distance 2.87 Å). The benzothiazole imidazole moiety interacts hydrophobically with Arg158, Tyr124, Tyr189, Gly122, Asn232, Ala123, and Leu289, while the thiazolidinone interact hydrophobically with the residues Arg213, Gln287 and Leu217 (Figure 1). These interactions stabilize the complex compound-enzyme and play a crucial role to the increased inhibitory action of the compound **5b**.

#### 2.3.2. Docking to Lanosterol 14α-Demethylase of *C. albicans*

As already mentioned in order to study the probable mechanism of antifungal activity all the synthesized compounds and reference drug were docked to lanosterol 14α-demethylase of *C. albicans* (Table 5).

**Figure 2:**
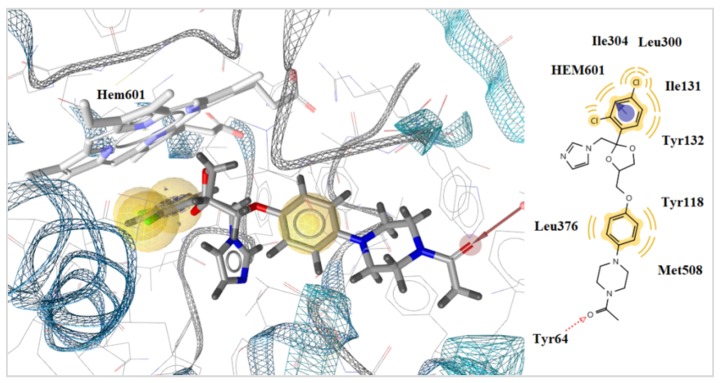
Docked conformation of ketoconazole in lanosterol 14α-demethylase of *C*. *albicans* (CYP51_ca_).

**Figure 3:**
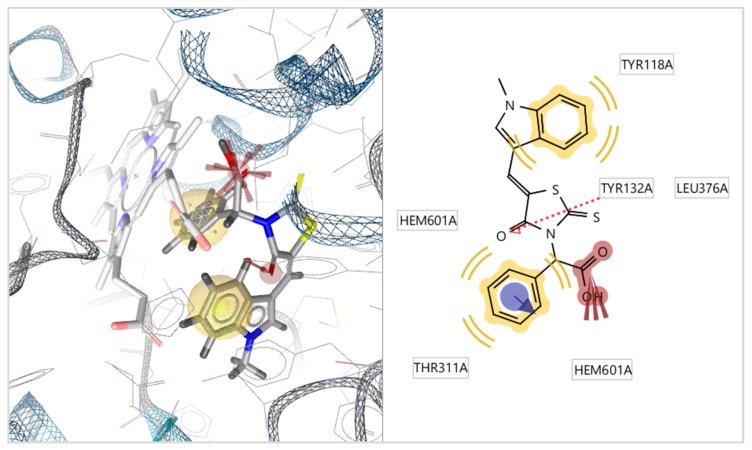
Docked conformation of compound **5f** in lanosterol 14α-demethylase of *C*. *albicans* (CYP51_ca_).

Docking results showed that the most active compound **5f** take place inside the enzyme alongside to heme group, interacting with the heme group of CYP51_Ca_ throughout its benzene ring and –NO_2_ group its forms pi and negative ionizable interactions with heme group respectively. A hydrogen bond interaction is formed between the oxygen atom of thiazolidinone moiety and the hydrogen atom of the side chain of Tyr134. Moreover, hydrophobic interactions between Tyr118, Leu376, and Thr311 and the benzene rings of the compound **5f** were detected. Interaction with the heme group was also observed with the benzene ring of ketoconazole which forms positive ionizable interactions (Figure 2 and Figure 3). However, compound **5f** forming more interaction than ketoconazole and more stable complex of ligand with enzyme. This is probably the reason why compound **8 5f** have better antifungal activity than ketoconazole.

## 3. Experimental Part

### 3.1. General Procedure for the Synthesis of 4-Oxo-2-thioxothiazolidin-3-ylalkanecarboxylic Acids 2**a**–**d** and 3**a**–**g**

A mixture of a corresponding amino acid (50 mmol), cooled solution of KOH in water (20 mL) (150 mmol in case of dicarboxylic acids) and CS_2_ (55 mmol) were stirred in a flat-bottomed flask until a solution was formed. A solution of monochloracetic acid (55 mmol) was added with stirring, pre-neutralized with sodium bicarbonate (55 mmol) in water (25 mL) and left at room temperature for 2 days.

Then, to the formed solution a 6N HCl solution (20 mL) was added and heated to boiling and kept at a slow boil for 1 h. After cooling, the precipitate formed was filtered off, dried and recrystallized, alternately, from diluted acetic acid, ethanol and toluene.

*(4-Oxo-2-thioxothiazolidin-3-yl)acetic acid* (**2a**). Yield 79%; m.p. 146–149 °C. ^1^H-NMR (400 MHz, DMSO-*d_6_*, ppm) δ 13.33 (s, 1H, COOH), 4.56 (s, 2H, CH_2_COOH), 4.41 (s, 2H, CH_2_). MS (ESI): *m*/*z* = 190.0 [M − H]^−^. Anal. Calcd. for C_5_H_5_NO_3_S_2_ (%): C, 31.41; H, 2.64; N, 7.32; S, 33.53 Found (%): C, 31.53; H, 2.59; N, 7.38; S, 33.46.

*3-(4-Oxo-2-thioxothiazolidin-3-yl)-propionic acid* (**2b**). Yield 80%; m.p. 158–160 °C. ^1^H-NMR (400 MHz, DMSO-*d*_6_, ppm) δ 12.50 (s, 1H, COOH), 4.22 (s, 2H, CH_2_), 4.05 (t, 2H, *J* = 8.0 Hz, CH_2_COOH), 2.51 (t, *J* = 8.0 Hz, 2H, NCH_2_). MS (ESI): *m*/*z* = 204.0 [M − H]^−^. Anal. Calcd. for C_6_H_7_NO_3_S_2_ (%): C, 35.11; H, 3.44; N, 6.82; S, 31.24 Found (%): C, 35.23; H, 3.51; N, 6.71; S, 31.18.

*4-(4-Oxo-2-thioxothiazolidin-3-yl)butyric acid* (**2c**). Yield 91%; m.p. 121–122 °C. ^1^H-NMR (400 MHz, DMSO-*d*_6_, ppm) δ 12.07 (s, 1H, COOH), 4.18 (s, 2H, SCH2), 3.86 (t, *J* = 7.0 Hz, 2H, CH_2_COOH), 2.21 (t, *J* = 7.4 Hz, 2H, NCH_2_), 1.79–1.71 (m, 2H, NCH_2_CH_2_CH_2_COOH).). MS (ESI): *m*/*z* = 218.0 [M − H]^−^. Anal. Calcd. for C_7_H_9_NO_3_S_2_ (%): C, 38.34; H, 4.14; N, 6.39; S, 29.24 Found (%):C, 38.25; H, 4.01; N, 6.45; S, 29.32

*6-(4-Oxo-2-thioxothiazolidin-3-yl)-hexanoic acid* (**2d**). Yield 88%; m.p. 86–89 °C. ^1^H-NMR (400 MHz, DMSO-*d*_6_, ppm) δ 12.01 (s, 1H, COOH), 4.25 (s, 2H, SCH_2_), 3.88–3.76 (m, 2H, CH_2_COOH), 2.19 (t, *J* = 7.3 Hz, 2H, NCH_2_), 1.61–1.40 (m, 4H, 2CH_2_), 1.34–1.18 (m, 2H, CH_2_). MS (ESI): *m*/*z* = 246.0 [M − H]^−^. Anal. Calcd. for C_9_H_13_NO_3_S_2_ (%): C, 43.71; H, 5.30; N, 5.66; S, 25.93 Found (%): C, 43.84; H, 5.24; N, 5.72; S, 26.04.

*2-(4-Oxo-2-thioxothiazolidin-3-yl)propionic acid* (**3a**). Yield 64%; m.p. 148–151 °C. ^1^H-NMR (400 MHz, DMSO-*d*_6_, ppm) δ 13.12 (s, 1H, COOH), 5.42 (q, *J* = 7.1 Hz, 1H, NCH), 4.31 (s, 2H, CH_2_), 1.43 (d, *J* = 7.1 Hz, 3H, CH_3_). MS (ESI): *m*/*z* = 204.0 [M − H]^−^. Anal. Calcd. for C_6_H_7_NO_3_S_2_ (%): C, 35.11; H, 3.44; N, 6.82; S, 31.24 Found (%): C, 35.04; H, 340; N, 6.88; S, 31.31.

*2-(4-Oxo-2-thioxothiazolidin-3-yl) pentanoic acid* (**3b**). Yield 67%; m.p. 87–90 °C. ^1^H-NMR (400 MHz, DMSO-*d*_6_, ppm) δ 13.16 (s, 1H, COOH), 5.44–5.30 (m, 1H, NCH), 4.37 (q, *J* = 18.7 Hz, 2H, SCH_2_), 2.03 (dd, *J* = 15.5, 7.5 Hz, 2H, CH_2_), 1.31–1.13 (m, 2H, CH_2_), 0.85 (t, *J* = 7.3 Hz, 3H, CH_3_). MS (ESI): *m*/*z* = 232.0 [M − H]^−^. Anal. Calcd. for C_8_H_11_NO_3_S_2_ (%): C, 41.19; H, 4.75; N, 6.00; S, 27.49 Found (%): C, 41.03; H, 4.82; N, 6.11; S, 27.41.

*4-Methylsulfanyl-2-(4-oxo-2-thioxothiazolidin-3-yl)butyric acid* (**3c**). Yield 65%; m.p. 116–119 °C. ^1^H-NMR (400 MHz, DMSO-*d*_6_, ppm) δ 13.27 (s, 1H, COOH), 5.55 (s, 1H, CH), 4.32 (s, 2H, SCH_2_), 2.47–2.22 (m, 4H, 2CH_2_), 2.01 (s, 3H, CH_3_). MS (ESI): *m*/*z* = 264.0 [M − H]^−^. Anal. Calcd. for C_8_H_11_NO_3_S_3_ (%): C, 36.21; H, 4.18; N, 5.28; S, 36.25 Found (%): C, 36.14; H, 4.27; N, 5.28; S, 36.19.

*(4-Oxo-2-thioxothiazolidin-3-yl)-phenylacetic acid* (**3d**). Yield 57%; m.p. 169–171 °C. ^1^H-NMR (400 MHz, DMSO-*d*_6_, ppm) δ 13.49 (s, 1H, COOH), 7.47–7.42 (m, 2H, Ph), 7.37–7.29 (m, 3H, Ph), 6.63 (s, 1H, NCH), 4.43 (s, 2H, CH_2_). MS (ESI): *m*/*z* = 266.0 [M − H]^−^. Anal. Calcd. for C_11_H_9_NO_3_S_2_ (%): C, 49.42; H, 3.39; N, 5.24; S, 23.99 Found (%): C, 49.31; H, 3.45; N, 5.35; S, 24.06.

*2-(4-Oxo-2-thioxothiazolidin-3-yl)-3-phenylpropionic acid***(3e**). Yield; 58%; m.p. 102–105 °C. ^1^H-NMR (400 MHz, DMSO-*d*_6_, ppm) δ 13.25 (s, 1H, COOH), 7.29–7.04 (m, 6H, C_6_H_5_), 5.64 (s, 1H, CH), 4.40–4.05 (m, 2H, SCH_2_), 3.49–3.15 (m, 2H, CH_2_). ^13^C-NMR (101 MHz, DMSO-d6, ppm) δ 203.07, 174.40, 169.31, 137.15, 129.48, 128.69, 127.08, 58.50, 35.12, 33.39. MS (ESI): *m*/*z* = 280.0 [M − H]^−^. Anal. Calcd. for C_12_H_11_NO_3_S_2_ (%): C, 51.23; H, 3.94; N, 4.98; S, 22.79 Found (%):C, 51.32; H, 3.89; N, 4.91; S, 22.86.

*2-(4-Oxo-2-thioxothiazolidin-3-yl) succinic acid* (**3f**). Yield 53%; m.p. 197–199 °C. ^1^H-NMR (400 MHz, DMSO-*d*_6_, ppm) δ 13.33 (s, 1H, COOH), 12.59 (s, 1H, COOH), 5.79 (s, 1H, NCH), 4.32 (s, 2H, CH_2_), 3.17 (dd, *J* = 16.2, 10.0 Hz, 1H, CH_2_), 2.69 (dd, *J* = 16.6, 3.3 Hz, 1H, CH_2_). MS (ESI): *m*/*z* = 248.0 [M − H]^−^. Anal. Calcd. for C_7_H_7_NO_5_S_2_ (%): C, 33.73; H, 2.83; N, 5.62; S, 25.73 Found (%): C, 33.83; H, 2.92; N, 5.55; S, 25.64.

*2-(4-Oxo-2-thioxothiazolidin-3-yl)pentanedioic aci*d (**3g**). Yield 79%; m.p. 146–149 °C. ^1^H-NMR (400 MHz, DMSO-*d*_6_, ppm) δ 5.44 (s, 1H, NCH), 4.30 (s, 2H, SCH_2_), 2.42–2.18 (m, 4H, 2CH_2_). MS (ESI): *m*/*z* = 262.0 [M − H]^−^. Anal. Calcd. for C_8_H_9_NO_5_S_2_ (%): C, 36.50; H, 3.45; N, 5.32; S, 24.36 Found (%): C, 36.62; H, 3.38; N, 5.35; S, 24.27.

### 3.2. General Procedure for the Synthesis of 5-(1-R^1^,5-R^2^,6-R^3^-1H-Indol-3-ylmethylene)-4-oxo-2- thioxothiazolidin-3-yl] Alkane Carboxylic Acids **4a**–**i** and **5a**–**k**

4-Oxo-2-thioxothiazolidin-3-ylalkanecarboxylic acid **2a**–**d** or **3a**–**g** (2 mmol), the corresponding indole-3-carbaldehyde (2.5 mmol), ammonium acetate (2 mmol) and ethanol (7 mL) were placed in a round-bottom flask under reflux. The reaction mixture was boiled for 2 to 3 h, cooled, the reaction product filtered off, washed with ethanol, water, dried and recrystallized from acetic acid or acetic acid - DMF.

*(Z)-[5-(1H-Indol-3-ylmethylene)-4-oxo-2-thioxothiazolidin-3-yl] acetic acid* (**4a**). Yield 99%; m.p. 277–278 °C. ^1^H-NMR (300 MHz, DMSO-*d*_6_, ppm) δ 12.11 (s, 1H, NH), 8.10 (s, 1H, CH=), 7.86 (d, *J* = 6.8 Hz, 1H, Ar), 7.73 (d, *J* = 2.9 Hz, 1H, Ar), 7.53–7.43 (m, 1H, Ar), 7.29–7.13 (m, 2H, Ar), 4.72 (s, 2H, NCH_2_). ^13^C-NMR (101 MHz, DMSO-d_6_, ppm) δ 192.21, 167.42, 166.02, 136.43, 130.90, 126.97, 126.73, 123.42, 121.60, 118.62, 113.88, 112.50, 111.06, 44.94. MS (ESI): *m*/*z* = 319.0 [M + H]^+^. Anal. Calcd. for C_14_H_10_N_2_O_3_S_2_ (%): C, 52.82; H, 3.17; N, 8.80; S, 20.14 Found (%): C, 52.71; H, 3.25; N, 8.87; S, 20.08.

*(Z)-[5-(1-Methyl-1H-indol-3-ylmethylene)-4-oxo-2-thioxothiazolidin-3-yl] acetic acid* (**4b**). Yield 99%; m.p. 273–275 °C. ^1^H-NMR (300 MHz, DMSO-*d*_6_, ppm) δ 8.05 (s, 1H, CH=), 7.89 (d, *J* = 7.7 Hz, 1H, Ar), 7.84 (s, 1H, Ar), 7.49 (d, *J* = 8.3 Hz, 1H, Ar), 7.37–7.20 (m, 2H, Ar), 4.71 (s, 2H, NCH_2_), 3.98 (s, 3H, CH_3_). ^13^C-NMR (101 MHz, DMSO-d_6_, ppm) δ 192.11, 167.43, 165.94, 136.99, 134.21, 127.24, 126.23, 123.47, 121.91, 118.66, 113.61, 110.98, 110.03, 44.27, 33.45. MS (ESI): *m*/*z* = 333.2 [M + H]^+^. Anal. Calcd. for C_15_H_12_N_2_O_3_S_2_ (%): C, 54.20; H, 3.64; N, 8.43; S, 19.29 Found (%): C, 54.33; H, 3.60; N, 8.34 S, 19.21.

*(Z)-[5-(6-Methoxy-1H-indol-3-ylmethylene)-4-oxo-2-thioxothiazolidin-3-yl] acetic acid* (**4c**). Yield 89%; m.p. > 270 °C. ^1^H-NMR (300 MHz, DMSO-*d*_6_, ppm) δ 12.00 (s, 1H, NH), 8.03 (s, 1H, CH=), 7.72 (d, *J* = 8.7 Hz, 1H, Ar), 7.62 (d, *J* = 2.8 Hz, 1H, Ar), 6.94 (d, *J* = 1.7 Hz, 1H, Ar), 6.85–6.78 (m, 1H, Ar), 4.70 (s, 2H, NCH_2_), 3.83 (s, 3H, CH_3_O). ^13^C-NMR (101 MHz, DMSO-d_6_, ppm) δ 192.19, 167.49, 166.04, 156.96, 137.42, 129.99, 127.24, 120.70, 119.40, 113.72, 111.60, 111.29, 95.51 55.34 (s), 44.97. MS (ESI): *m*/*z* = 349.2 [M + H]^+^. Anal. Calcd. for C_15_H_12_N_2_O_4_S_2_ (%): C, 51.71%; H, 3.47; N, 8.04; S, 18.41 Found (%): C, 51.69%; H, 3.40; N, 8.11; S, 18.39.

*(Z)-3-[5-(1-Methyl-1H-indol-3-ylmethylene)-4-oxo-2-thioxothiazolidin-3-yl] propionic acid* (**4d**). Yield 83%; m.p. 246–248 °C. ^1^H-NMR (300 MHz, DMSO-*d*_6_, ppm) δ 8.01 (s, CH=), 7.88 (d, *J* = 7.6 Hz, 1H, Ar), 7.80 (s, 1H, Ar), 7.48 (d, *J* = 8.0 Hz, 1H, Ar), 7.33–7.21 (m, 2H, Ar), 4.30 (t, *J* = 9.0 Hz, 2H, CH_2_COOH), 3.97 (s, 3H, CH_3_), 2.63 (t, *J* = 9.0 Hz, 2H, NCH_2_). ^13^C-NMR (101 MHz, DMSO-d_6_, ppm) δ 192.02, 171.83, 166.29, 136.94, 133.94, 127.25, 125.48, 123.41, 121.85, 118.60, 114.13, 110.95, 110.06, 33.42, 30.94. MS (ESI): *m*/*z* = 347.0 [M + H]^+^. Anal. Calcd. for C_16_H_14_N_2_O_3_S_2_ (%): C, 55.47; H, 4.07; N, 8.09; S, 18.51 Found (%):C, 55.38; H, 4.01; N, 7.98; S, 18.46.

*(Z-)3-[5-(5-Methoxy-1H-indol-3-ylmethylene)-4-oxo-2-thioxothiazolidin-3-yl] propionic acid* (**4e**). Yield 96%; m.p. 226–228 °C. ^1^H-NMR (300 MHz, DMSO-*d*_6_, ppm) δ 12.07 (s, 1H, NH), 8.05 (s, 1H, CH=), 7.62 (d, *J* = 3.1 Hz, 1H, Ar), 7.37–7.31 (m, 2H, Ar), 6.81 (dd, *J* = 8.8, 2.1 Hz, 1H, Ar), 4.27 (t, *J* = 9.0 Hz 2H, CH_2_COOH), 3.86 (s, 3H, CH_3_O), 2.61 (t, *J* = 9.0 Hz, 2H, NCH_2_). ^13^C-NMR (101 MHz, DMSO-d_6_, ppm) δ 192.01, 171.74, 155.31, 131.15, 130.55, 127.73, 126.72, 114.45, 113.53, 113.50, 113.31, 111.15, 100.37, 55.48, 30.93. MS (ESI): *m*/*z* = 363.0 [M + H]^+^. Anal. Calcd. for C_16_H_14_N_2_O_4_S_2_ (%): C, 53.03; H, 3.89; N, 7.73; S, 17.69 Found (%):C, 53.14; H, 3.91; N, 7.65; S, 17.58.

*(Z)-4-[5-(1-Methyl-1H-indol-3-ylmethylene)-4-oxo-2-thioxothiazolidin-3-yl] butyric acid* (**4f**). Yield 96%; m.p. 229–230 °C. ^1^H-NMR (300 MHz, DMSO-*d*_6_, ppm) δ 8.00 (s, 1H, CH=), 7.88 (d, *J* = 7.3 Hz, 1H, Ar), 7.79 (s, 1H, Ar), 7.48 (d, *J* = 7.6 Hz, 1H, Ar), 7.33–7.22 (m, 2H, Ar), 4.14 (t, *J* = 7.1 Hz, 2H, CH_2_COOH), 3.97 (s, 3H CH_3_), 2.30 (t, *J* = 7.4 Hz, 2H, NCH_2_), 2.04–1.89 (m, 2H, NCH_2_CH_2_CH_2_COOH). ^13^C-NMR (101 MHz, DMSO-d_6_, ppm) δ 192.41, 173.71, 166.74, 136.95, 133.89, 127.26, 125.32, 123.40, 121.83 (s), 118.62, 114.31, 110.95, 110.10, 43.53, 33.42, 31.01, 22.13. MS (ESI): *m*/*z* = 361.2 [M + H]^+^. Anal. Calcd. for C_17_H_16_N_2_O_3_S_2_ (%): C, 56.65; H, 4.47; N, 7.77; S, 17.79 Found (%): C, 56.53; H, 4.41; N, 7.85; S, 17.71.

*(Z)-4-[5-(5-Methoxy-1H-indol-3-ylmethylene)-4-oxo-2-thioxothiazolidin-3-yl] butyric acid* (**4g**). Yield 87%; m.p. 214–216 °C. ^1^H-NMR (300 MHz, DMSO-*d*_6_, ppm) δ 11.96 (s, 1H, NH), 7.97 (s, 1H, CH=), 7.72 (d, *J* = 8.7 Hz, 1H, Ar), 7.57 (d, *J* = 2.8 Hz, 1H, Ar), 6.93 (d, *J* = 1.6 Hz, 1H, Ar), 6.81 (dd, *J* = 8.7, 1.9 Hz, 1H, Ar), 4.12 (t, *J* = 7.0 Hz, 2H, CH_2_COOH), 3.83 (s, 3H, CH_3_O), 2.29 (t, *J* = 7.3 Hz, 2H, NCH_2_), 1.95 (p, *J* = 7.0 Hz, 2H, NCH_2_CH_2_CH_2_COOH). ^13^C-NMR (101 MHz, DMSO-d_6_, ppm) δ 192.37, 173.66, 166.74, 155.27, 131.11, 130.50, 127.73, 126.58, 113.64, 113.52, 113.31, 111.17, 100.30, 55.45, 43.49, 30.95, 22.08. MS (ESI): *m*/*z* = 377.2 [M + H]^+^. Anal. Calcd. for C_17_H_16_N_2_O_4_S_2_ (%): C, 54.24; H, 4.28; N, 7.44; S, 17.03 Found (%):C, 54.18; H, 4.22; N, 7.48; S, 17.15.

*(Z)-4-[5-(6-Methoxy-1H-indol-3-ylmethylene)-4-oxo-2-thioxothiazolidin-3-yl] butyric acid* (**4h**). Yield 84%; m.p. 213–215 °C. ^1^H-NMR (300 MHz, DMSO-*d*_6_, ppm) δ 11.96 (s, 1H, NH), 7.97 (s, 1H, CH=), 7.71 (d, *J* = 8.7 Hz, 1H, Ar), 7.56 (d, *J* = 2.5 Hz, 1H, Ar), 6.92 (s, 1H, Ar), 6.81 (d, *J* = 8.7 Hz, 1H, Ar), 4.12 (t, *J* = 6.9 Hz, 2H, CH_2_COOH), 3.83 (s, 3H, CH_3_), 2.28 (t, *J* = 7.3 Hz, 2H, NCH_2_), 2.01–1.87 (m, 2H, NCH_2_CH_2_CH_2_COOH). ^13^C-NMR (101 MHz, DMSO-d_6_, ppm) δ 192.39, 173.61, 166.73, 156.85, 137.31, 129.61, 126.24, 120.65, 119.26, 114.34, 111.44, 111.26, 95.41, 55.28, 43.53, 30.99, 22.09. MS (ESI): *m*/*z* = 377.2 [M + H]^+^. Anal. Calcd. for C_17_H_16_N_2_O_4_S_2_ (%): C, 54.24; H, 4.28; N, 7.44; S, 17.03 Found (%):C, 54.31; H, 4.20; N, 7.38; S, 17.09.

*(Z)-6-[5-(1H-Indol-3-ylmethylene)-4-oxo-2-thioxothiazolidin-3-yl] hexanoic acid* (**4i**). Yield 98%; m.p. 205–207 °C. ^1^H-NMR (300 MHz, DMSO-*d*_6_, ppm) δ 12.17 (s, 1H, NH), 8.03 (s, 1H, CH=), 7.85 (d, *J* = 6.8 Hz, 1H, Ar), 7.68 (d, *J* = 2.9 Hz, 1,H, Ar), 7.48–7.46 (m, 1H, 1H, Ar), 7.25–7.13 (m, 2H, Ar), 4.12–3.97 (m, 2H, CH_2_COOH), 2.20 (t, *J* = 7.3 Hz, NCH_2_), 1.78–1.55 (m, 4H, 2CH_2_), 1.48–1.34 (m, 2H, CH_2_). ^13^C-NMR (101 MHz, DMSO-d_6_, ppm) δ 192.23, 174.33, 166.66, 136.43, 130.58, 126.77, 126.17, 123.38, 121.54 (s), 118.52, 114.47, 112.58, 111.15, 43.97, 33.44, 26.21, 25.73, 24.07. MS (ESI): *m*/*z* = 375.2 [M + H]^+^. Anal. Calcd. for C_18_H_18_N_2_O_3_S_2_ (%): C, 57.73; H, 4.84; N, 7.48; S, 17.12 Found (%): C, 57.67; H, 4.79; N, 7.53; S, 17.01.

*(Z)-2-[5-(1H-Indol-3-ylmethylene)-4-oxo-2-thioxothiazolidin-3-yl] propionic acid* (**5a**) Yield 90%; m.p. > 270 °C. ^1^H-NMR (400 MHz, DMSO-*d*_6_, ppm) δ 12.43 (s, 1H, NH), 8.10 (s, 1H, CH=), 8.00–7.93 (m, 2H, Ar), 7.52 (d, *J* = 7.9 Hz, 1H, Ar), 7.25 (dt, *J* = 14.7, 7.1 Hz, 2H, Ar), 5.62 (q, *J* = 6.9 Hz, 1H, NCH), 1.55 (d, *J* = 7.1 Hz, 3H, CH_3_). ^13^C-NMR (101 MHz, DMSO-d_6_, ppm) δ 191.83, 169.83, 165.96, 136.46, 130.94 (s), 126.86, 126.79, 123.47, 121.66, 118.59, 113.38, 112.64, 111.12, 52.73, 13.58. Anal. MS (ESI): *m*/*z* = 333.2 [M + H]^+^. Calcd. for C_15_H_12_N_2_O_3_S_2_ (%): C, 54.20; H, 3.64; N, 8.43; S, 19.29 Found (%): C, 54.14; H, 3.72; N, 8.51; S, 19.20.

*(Z)-2-[5-(1H-Indol-3-ylmethylene)-4-oxo-2-thioxothiazolidin-3-yl] pentanoic acid* (**5b**). Yield 90%; m.p. 257–259 °C. ^1^H-NMR (300 MHz, DMSO-*d*_6_, ppm) δ 12.10 (s, 1H, Ar), 8.03 (s, 1H, CH=), 7.85 (d, *J* = 7.0 Hz, 1H, Ar), 7.71 (d, *J* = 2.9 Hz, 1H, Ar), 7.52–7.46 (m, 1H, Ar), 7.27–7.16 (m, 2H, Ar), 5.55 (dd, *J* = 9.2, 5.6 Hz, 1H, NCH), 2.38–2.12 (m, 2H, CH_2_), 1.49–1.21 (m, 2H, CH_2_), 0.97 (t, *J* = 7.3 Hz, 3H, CH_3_). ^13^C-NMR (101 MHz, DMSO-d_6_, ppm) δ 192.59, 169.44, 166.27, 136.49, 131.02, 127.05, 126.79, 123.48, 121.67, 118.59, 113.06, 112.65, 111.16, 56.92, 29.71, 19.04, 13.65. Anal. MS (ESI): *m*/*z* = 361.0 [M + H]^+^. Anal. Calcd. for C_17_H_16_N_2_O_3_S*_2_* (%): C, 56.65; H, 4.47; N, 7.77; S, 17.79 Found (%): C, 56.55; H, 4.41; N, 7.84; S, 17.70.

*(Z)-2-[5-(1H-Indol-3-ylmethylene)-4-oxo-2-thioxothiazolidin-3-yl]-4-methylsulfanylbutyric acid* (**5c**). Yield 98%; m.p. 204–205 °C. ^1^H-NMR (400 MHz, DMSO-*d*_6_, ppm) δ 12.43 (s, 1H, NH), 8.10 (s, 1H, CH=), 7.99–7.94 (m, 2H, Ar), 7.52 (d, *J* = 7.7 Hz, 1H, Ar), 7.26 (ddd, *J* = 14.9, 13.8, 6.6 Hz, 2H, Ar), 5.74 (s, 1H, NCH), 3.34 (b. s, 4H, 2CH_2_), 2.02 (s, 3H, CH_3_). ^13^C-NMR (101 MHz, DMSO-d_6_, ppm) δ 192.54, 169.20, 166.35, 136.41, 130.90, 126.83, 126.74, 123.42, 121.61, 118.54, 113.30, 112.59, 111.09, 56.08, 30.13, 27.16, 14.57. Anal. MS (ESI): *m*/*z* = 393.0 [M + H]^+^. Anal. Calcd. for C_17_H_16_N_2_O_3_S_3_ (%): C, 52.02; H, 4.11; N, 7.14; S, 24.51 Found (%): C 51.94; H, 4.08; N, 7.10; S, 24.42.

*(Z)-2-[5-(1-Methyl-1H-indol-3-ylmethylene)-4-oxo-2-thioxothiazolidin-3-yl]-4-methylsulfanyl-butyric acid* (**5d**). Yield 94%; m.p. 238–240 °C. ^1^H-NMR (300 MHz, DMSO-*d*_6_, ppm) δ 8.00 (s, 1H, CH=), δ 7.89 (s, 1H, Ar), 7.86 (s, 1H, Ar), 7.49 (d, *J* = 8.0 Hz, 1H, Ar), 7.34–7.20 (m, 2H, Ar), 5.70 (d, *J* = 5.7 Hz, 1H, NCH), 3.98 (s, CH_3_N), 2.65–2.34 (m, 4H, CH_2_), 2.07 (s, 3H, CH_3_). ^13^C-NMR (101 MHz, DMSO-d_6_, ppm) δ 169.27, 166.37, 137.04, 134.25, 127.30, 126.19, 123.53, 121.99, 118.73, 113.13, 111.07, 110.12, 56.13, 33.52, 30.20, 27.22, 14.62. MS (ESI): *m*/*z* = 407.0 [M + H]^+^. Anal. Calcd. for C_18_H_18_N_2_O_3_S_3_ (%): C, 53.18; H, 4.46; N, 6.89; S, 23.66 Found (%): C 53.23; H, 4.53; N, 6.78; S, 23.54.

*(Z)-2-[5-(5-Methoxy-1H-indol-3-ylmethylene)-4-oxo-2-thioxothiazolidin-3-yl]-4-methylsulfanyl-butyric acid* (**5e**). Yield 93%; m.p. 222–223 °C. ^1^H-NMR (300 MHz, DMSO-*d*_6_, ppm) δ 12.11 (s, 1H, NH), 8.07 (s, 1H, CH=), 7.66 (s, 1H, Ar), 7.35 (d, *J* = 8.8 Hz, 2H, Ar), 6.81 (d, *J* = 8.7 Hz, 1H Ar), 5.69 (s, 1H, NCH), 3.86 (s, 3H, CH_3_O), 2.61–2.33 (m, 4H, 2CH_2_), 2.06 (s, 1H, CH_3_). ^13^C-NMR (101 MHz, DMSO-d_6_, ppm) δ 192.50, 169.18, 166.31, 155.38, 131.18, 130.85, 127.78, 127.38, 113.56, 113.36, 112.50, 111.20, 100.50, 56.08, 55.49, 30.17, 27.21, 14.59. MS (ESI): *m*/*z* = 423.0 [M + H]^+^. Anal. Calcd. for C_18_H_18_N_2_O_4_S_3_ (%): C, 51.17; H, 4.29; N, 6.63; S, 22.76 Found (%): C 51.25; H, 4.21; N, 6.69; S, 22.69.

*(Z-)[5-(1-Methyl-1H-indol-3-ylmethylene)-4-oxo-2-thioxothiazolidin-3-yl]-phenylacetic acid* (**5f**). Yield 90%; m.p. > 270 °C. ^1^H-NMR (300 MHz, DMSO-*d*_6_, ppm) δ 8.02 (s, 1H, CH=), 7.89–7.82 (m, 2H, Ar), 7.62–7.56 (m, 2H, Ar), 7.48 (d, *J* = 7.9 Hz, 1H, Ar), 7.38–7.21 (m, 5H, Ar), 6.76 (s, 1H, NCH), 3.97 (s, 3H, CH_3_). ^13^C-NMR (101 MHz, DMSO-d_6_, ppm) δ 191.89, 167.96, 165.89, 137.05, 134.46, 133.77, 129.70, 128.32, 128.11, 127.31, 126.91, 123.57, 122.07, 118.74, 112.35, 111.07, 110.14, 60.06, 33.53. MS (ESI): *m*/*z* = 409.2 [M + H]^+^. Anal. Calcd. for C_21_H_16_N_2_O_3_S_2_ (%): C, 61.75; H, 3.95; N, 6.86; S, 15.70 Found (%): C 61.67; H, 3.89; N, 6.94; S, 15.81.

*(Z)-2-[5-(1H-Indol-3-ylmethylene)-4-oxo-2-thioxothiazolidin-3-yl]-3-phenylpropionic acid.* (**5g**). Yield 97%; m.p. 271 °C _decomp_. ^1^H-NMR (400 MHz, DMSO-*d*_6_, ppm) δ 12.42 (s, 1H, NH), 8.06 (s, 1H, CH=), 7.96 (d, *J* = 7.6 Hz, 1H, Ar), 7.88 (d, *J* = 2.7 Hz, 1H, Ar), 7.51 (d, *J* = 7.7 Hz, 1H, Ar), 7.30–7.10 (m, 7H, Ar), 5.88 (s, 1H, NCH), 3.52 (d, *J* = 5.3 Hz, 2H, CH_2_). ^13^C-NMR (101 MHz, DMSO-d_6_, ppm) δ 192.03, 168.99, 166.29, 136.72, 136.45, 131.00, 129.04, 128.24, 126.80, 126.75, 126.66, 123.49, 121.67, 118.60, 112.91, 112.63, 111.07, 58.04, 33.22. MS (ESI): *m*/*z* = 409.2 [M + H]^+^. Anal. Calcd. for C_21_H_16_N_2_O_3_S_2_ (%): C, 61.75; H, 3.95; N, 6.86; S, 15.70 Found (%): C 61.74; H, 3.99; N, 6.91; S, 15.64

*(Z)-2-[5-(1-Methyl-1H-indol-3-ylmethylene)-4-oxo-2-thioxothiazolidin-3-yl]-3-phenylpropionic acid* (**5h**). Yield 99%; m.p. 227–228 °C. ^1^H-NMR (300 MHz, DMSO-*d*_6_, ppm) δ 7.95 (s, 1H, CH=), 7.87 (d, *J* = 7.4 Hz, 1H, Ar), 7.78 (s, 1H, Ar), 7.48 (d, *J* = 7.7 Hz, 1H, Ar), 7.34–7.21 (m, 2H, Ar), 7.20–7.12 (m, 5H, Ar), 5.83 (dd, *J* = 9.2, 6.7 Hz, 1H, NCH), 3.96 (s, 3H, CH_3_N), 3.65–3.46 (m, 2H, CH_2_). ^13^C-NMR (101 MHz, DMSO-d_6_, ppm) δ 191.94, 169.06, 166.24, 137.02, 136.70, 134.32, 129.05, 128.24, 127.25, 126.67, 126.16, 123.55, 122.00, 118.74, 112.64, 111.05, 110.03, 58.00, 33.49, 33.19. MS (ESI): *m*/*z* = 423.0 [M + H]^+^. Anal. Calcd. for C_22_H_18_N_2_O_3_S_2_ (%): C, 62.54; H, 4.29; N, 6.63; S, 15.18 Found (%): C 62.47 H, 4.35; N, 6.56; S, 15.09.

*(Z)-2-[5-(1H-Indol-3-ylmethylene)-4-oxo-2-thioxothiazolidin-3-yl] succinic acid* (**5i**). Yield 99%; m.p. 241 °C _decomp_. ^1^H-NMR (300 MHz, DMSO-*d*_6_, ppm) δ 12.12 (s, 1H, NH), 8.05 (s, 1H, CH=), 7.86 (d, *J* = 6.8 Hz, 1H, Ar), 7.72 (d, *J* = 2.9 Hz, 1H, Ar), 7.53–7.46 (m, 1H, Ar), 7.28–7.15 (m, 2H, Ar), 5.98 (t, *J* = 6.9 Hz, 1H, NCH), 3.30 (dd, *J* = 16.5, 7.7 Hz, 1H, CH_2_), 2.82 (dd, *J* = 16.5, 6.0 Hz, 1H, CH_2_). ^13^C-NMR (101 MHz, DMSO-d_6_, ppm) δ 191.93, 171.24, 168.83, 166.19, 136.43, 130.92, 126.92, 126.73, 123.43, 121.62, 118.54, 113.32, 112.60, 111.07, 53.20, 33.14. MS (ESI): *m*/*z* = 377.0 [M + H]^+^. Anal. Calcd. for C_16_H_12_N_2_O_5_S_2_ (%): C, 51.06; H, 3.21; N, 7.44; S, 17.04 Found (%): C 51.12; H, 3.16; N, 7.38; S, 17.13.

*(Z)-2-[5-(1H-Indol-3-ylmethylene)-4-oxo-2-thioxothiazolidin-3-yl]-pentanedioic acid* (**5j**). Yield 99%; m.p. 253–255 °C. ^1^H-NMR (300 MHz, DMSO-*d*_6_, ppm) δ 12.21 (s, 1H, NH), 8.04 (s, 1H, CH=), 7.86 (d, *J* = 6.8 Hz, 1H, Ar), 7.74 (d, *J* = 3.0 Hz, 1H, Ar), 7.53–7.47 (m, 1H, Ar), 7.27–7.15 (m, 2H, Ar), 5.59 (dd, *J* = 9.3, 5.1 Hz, 1H, NCH), 2.56–2.45 (m, 2H, CH_2_), 2.30–2.23 (m, 2H, CH_2_). ^13^C-NMR (101 MHz, DMSO-d_6_, ppm) δ 192.67, 173.56, 169.18, 166.27, 137.07, 134.22, 127.30, 126.14, 123.52, 121.98, 118.70, 113.18, 111.05, 110.14, 56.57, 33.50, 30.25, 23.09. MS (ESI): *m*/*z* = 391.0 [M + H]^+^. Anal. Calcd. for C_17_H_14_N_2_O_5_S_2_ (%): C, 52.30; H, 3.61; N, 7.17; S, 16.42 Found (%): 52.21; H, 3.75; N, 7.12; S, 16.51.

*(Z-)2-[5-(1-Methyl-1H-indol-3-ylmethylene)-4-oxo-2-thioxothiazolidin-3-yl]-pentanedioic acid* (**5k**). Yield 76%; m.p. 256–258 °C. ^1^H-NMR (300 MHz, DMSO-*d*_6_, ppm) δ 8.00 (s, 1H, CH=), 7.90–7.83 (m, 2H, Ar), 7.49 (d, *J* = 7.9 Hz, 1H Ar), 7.35–7.20 (m, 2H Ar), 5.59 (dd, *J* = 9.2, 5.0 Hz, 1H, NCH), 2.56–2.45 (m, 2H, CH_2_), 2.31–2.22(m, 2H, CH_2_). ^13^C-NMR (101 MHz, DMSO-d_6_, ppm) δ 192.60, 173.49, 169.11, 166.21, 137.02, 134.16, 127.24, 126.09, 123.47, 121.92, 118.65, 113.13, 111.00, 110.09, 56.51, 33.45, 30.20, 23.04. MS (ESI): *m*/*z* = 405.0 [M + H]^+^. Anal. Calcd. for C_18_H_16_N_2_O_5_S_2_ (%): C, 53.45; H, 3.99; N, 6.93; S, 15.85 Found (%): 53.52; H, 4.05; N, 6.99; S, 15.74.(Spectra see in supplementary)

### 3.3. Biological Evaluation

#### 3.3.1. Antibacterial Activity

The following Gram-negative bacteria: *Escherichia coli* (ATCC 35210), *Enterobacter cloacae (clinical isolate), Salmonella typhimurium* (ATCC 13311), as well as Gram-positive bacteria*: Listeria monocytogenes* (NCTC 7973), *Bacillus cereus* (clinical isolate), and *Staphylococcus aureus* (ATCC 6538) were used. The organisms were obtained from the Mycological Laboratory, Department of Plant Physiology, Institute for Biological Research “Siniša Stankovic”, Belgrade, Serbia.

The minimum inhibitory (MIC) and minimum bactericidal (MBC) concentrations were determined by the modified microdilution method as previously reported [36,37,38].

Resistant strains used in microdilution assay were isolates of *S. aureus* (strain isolated from cow), *E. coli* (strain isolated form pig) and *P. aeruginosa* (strain isolated from cat) obtained as described in Kartsev et al. [39].

#### 3.3.2. Inhibition of Biofilm Formation

Method was performed as described [40] with some modifications. Briefly, *P. aeruginosa* resistant strain was incubated with MIC and subMIC of tested compounds in Triptic soy broth enriched with 2% glucose at 37 °C for 24 h. After 24 h, each well was washed twice with sterile PBS (Phosphate buffered saline, pH 7.4) and fixed with methanol for 10 min. Methanol was then removed and the plate was air dried. Biofilm was stained with 0.1% crystal violet (Bio-Merieux, Craponne, France) for 30 min. Wells were washed with water, air dried and 100 μL of 96% ethanol (Zorka, Serbia) was added. The absorbance was read at 620 nm on a Multiskan™ FC Microplate Photometer, Thermo Scientific™ (Waltham, MA, USA). The percentage of inhibition of biofilm formation was calculated by the formula:

[(A_620_ control − A_620_ sample)/A_620_ control] × 100.

#### 3.3.3. Antifungal Activity

For the antifungal bioassays, six fungi were used: *Aspergillus niger* (ATCC 6275), *Aspergillus fumigatus* (ATCC 1022), *Aspergillus versicolor* (ATCC 11730), *Penicillium funiculosum* (ATCC 36839), *Trichoderma viride* (IAM 5061), *Penicillium verrucosum var. cyclopium* (food isolate). The organisms were obtained from the Mycological Laboratory, Department of Plant Physiology, Institute for Biological Research ‘‘Siniša Stankovic,’’ Belgrade, Serbia. All experiments were performed in duplicate and repeated three times [35,39].

### 3.4. Docking Studies

Τhe program AutoDock 4.2^®^ software (version 4.2.6, San Diego, California, CA, USA) was used for the docking stimulation. The free energy of binding (ΔG) of *E. coli* DNA GyrB, Thymidylate kinase, *E. coli* MurA, *E. coli* primase, *E. coli* MurB, DNA topoIV and CYP51 of *C*. *albicans* in complex with the inhibitors were generated using this molecular docking program. The X-ray crystal structures data of all the enzymes used were obtained from the Protein Data Bank (PDB ID: 1KZN, AQGG, 1DDE, JV4T, 2Q85, 1S16 and 5V5Z respectively). All procedures were performed according to our previous paper [38].

## 4. Conclusions

The range of twenty new 5-(1*H*-Indol-3-ylmethylene)-4-oxo-2-thioxothiazolidin-3-yl) alkancarboxylic acid derivatives were synthesized and evaluated for their antimicrobial activity exhibiting a remarkable inhibition of the growth of a wide spectrum of Gram-positive and Gram-negative bacteria and fungi. All compounds displayed better antibacterial activity than ampicillin against all bacteria tested, while eighteen out of twenty showed better activity than streptomycin against *S. aureus, En. cloacae, P. aeruginosa*, *L. monocytogenes,* and *E. coli.* The best antibacterial activity was achieved for compound **5b** (*Z*)-2-[5-(1*H*-Indol-3-ylmethylene)-4-oxo-2-thioxothiazolidin-3-yl] pentanoic acid. Three the most active compounds **4h**, **5b** and **5g** tested against three resistant strains: Methicillin resistant *S. aureus, MRSA, P. aeruginosa and E. coli* appeared to be more potent against MRSA than ampicillin, while streptomycin did not show any bactericidal activity. All three compounds displayed better activity also against resistant strains *P. aeruginosa* and *E. coli* than ampicillin. These compounds were tested also for their effect on biofilm formation. All compounds were able to inhibit biofilm formation 2 to 4 times more than both reference drugs. The most sensitive bacterium was found to be *P. aeruginosa*, while *M. flavus* was the most resistant.

The evaluation of antifungal activity revealed that all compounds appeared to be more potent than ketoconazole and bifonazole used as reference drugs. The most potent compound appeared to be **5f** (*Z*-)[5-(1-Methyl-1*H*-indol-3-ylmethylene)-4-oxo-2-thioxothiazolidin-3-yl]-phenylacetic acid.

The most sensitive fungal to compounds tested was *T. viride*, while *A fumigatus* was found to be the most resistant one. It should be mentioned that the growth of both Gram-positive and Gram-negative bacteria as well as fungi showed different sensitivity towards compounds tested.

Docking analysis to different antibacterial targets (MurB, Gyrase, Thymidylate kinase) demonstrated that *E. coli* Mur B inhibition, probably, is involved in antibacterial mechanism of action of compounds tested. On the other hand, docking analysis to 14α-lanosterol demethylase (CYP51) and tetrahydrofolate reductase of *Candida albicans* specified a probable implication of CYP51 reductase at the antifungal activity of the compounds.

## Figures and Tables

**Table 1:** Antibacterial activity of compounds (μM × 10^−2^).

R.br		*B.c*	*M.f*	*S.a*	*L.m*	*En.cl*	*P.a*	*S.t*	*E. coli*
**4a**	MIC	4.72	28.30	6.92	9.43	4.72	4.72	4.72	28.30
	MBC	9.43	37.72	9.43	18.86	9.43	9.43	9.43	37.72
**4b**	MIC	4.51	13.54	2.26	9.03	2.26	2.26	18.05	7.47
	MBC	9.03	18.05	4.51	18.05	4.51	4.51	36.10	18.05
**4c**	MIC	8.61	12.91	8.61	8.61	4.31	4.31	4.31	12.91
	MBC	17.22	17.22	17.22	17.22	8.61	8.61	8.61	17.22
**4d**	MIC	8.66‘	4.33	8.66	8.66‘	4.33	4.33	8.66‘	8.66‘
	MBC	17.32	8.66‘	17.32	17.32	8.66	8.66‘	17.32	17.32
**4e**	MIC	8.28	4.14	4.14	8.28	8.28	4.14	8.28	12.42
	MBC	16.57	8.28	8.28	16.57	16.57	8.28	16.57	16.57
**4f**	MIC	4.16	8.32	4.16	16.64	4.16	4.16	16.64	8.32
	MBC	8.32	16.64	8.32	33.2	8.32	8.32	33.29	16.64
**4g**	MIC	7.97	3.98	3.98	11.95	3.98	3.98	14.07	1.59
	MBC	15.94	7.97	7.97	15.94	7.97	7.97	15.94	1.99
**4h**	MIC	3.98	1.99	1.99	3.98	3.98	3.98	3.98	3.98
	MBC	7.97	3.98	3.98	7.97	7.97	7.97	7.97	7.97
**4i**	MIC	4.00	5.87	2.94	16.02	5.87	5.87	16.02	5.87
	MBC	8.01	8.01	4.00	32.04	8.01	8.01	32.04	8.01
**5a**	MIC	6.63	18.07	6.63	9.04	3.31	4.52	9.04	18.07
	MBC	9.04	36.14	9.04	18.07	4.52	9.04	18.07	36.14
**5b**	MIC	1.11	12.50	0.56	2.22	1.67	2.22	2.22	4.17
	MBC	2.08	16.67	2.08	4.17	2.08	8.33	4.17	8.33
**5c**	MIC	7.64	3.82	7.64	7.64	3.82	7.64	7.64	3.82
	MBC	15.29	7.64	15.29	15.29	7.64	15.29	15.29	7.64
**5d**	MIC	3.69	3.69	3.69	7.38	3.69	3.69	7.38	3.69
	MBC	7.38	7.38	7.38	14.76	7.38	7.38	14.76	7.38
**5e**	MIC	7.10	5.21	7.10	7.10	3.55	3.55	3.55	7.10
	MBC	14.20	7.10	14.20	14.20	7.10	7.10	7.10	14.20
**5f**	MIC	11.02	11.02	5.39	7.34	5.39	5.39	14.69	11.02
	MBC	14.69	14.69	7.34	14.69	7.34	7.34	29.38	14.69
**5g**	MIC	1.96	11.03	1.96	1.96	3.68	1.96	1.96	11.03
	MBC	3.68	14.72	3.68	3.68	7.36	3.68	3.68	14.72
**5h**	MIC	7.10	7.10	3.55	7.10	7.10	7.10	7.10	7.10
	MBC	14.20	14.20	7.10	14.20	14.20	14.20	14.20	14.20
**5i**	MIC	7.97	11.96	3.99	15.94	7.97	7.97	15.94	11.96
	MBC	15.94	15.94	7.97	31.88	15.94	15.94	31.88	15.94
**5j**	MIC	15.37	23.05	7.68	30.74	7.68	11.53	15.37	30.74
	MBC	30.74	30.74	15.37	61.48	15.37	15.37	30.74	61.48
**5k**	MIC	3.71	7.42	3.71	7.42	7.42	5.44	7.42	11.25
	MBC	7.42	14.88	7.42	14.88	14.88	7.42	14.88	14.88
**Amp.**	MIC	24.80	24.80	24.80	37.20	24.80	74.40	24.80	37.20
	MBC	37.20	37.20	37.20	74.40	37.20	124.0	49.20	49.20
**Strept.**	MIC	4.30	8.60	17.20	25.80	4.30	17.20	17.20	17.20
	MBC	8.60	17.20	34.40	51.60	8.60	34.40	34.40	34.40

B.c.—*Bacillus cereus*, M.f.—M. flavus, S.a.—*Staphylococcus aureus*, l.m.—*Listeria monocytogenes*, E.c.—*Escherichia coli*, En.c.—*Enterobacter cloacae*, P.a.—*Pseudomonas aeruginosa*, S.t.—*Salmonella typhimurium*. Relative standard deviations were all < 2.0. Amp.: Ampicillin, Strept.: Streptomycin.

**Table 2:** Antibacterial activity against resistant strains and effect on biofilm formation(mg/mL).

Compounds	Resistant Strains				Biofilm Formation	
		*MRSA*	*P.a.*	*E.c.*	MIC	0.5MIC
**4h**	MIC	0.5	0.12	0.5	17.14	NE
	MBC	1.0	0.25	1.0		
**5b**	MIC	0.25	0.12	0.25	37.93	22.97
	MBC	0.5	0.25	0.5		
**5g**	MIC	0.5	0.12	0.5	30.59	11.02
	MBC	1.0	0.25	1.0		
Streptomycin	MIC	0.1	0.05	0.1	71.94	55.42
	MBC	/	0.1	0.2		
Ampicilline	MIC	/	0.2	0.2	67.36	30.35
	MBC	/	/	/		

*MRSA*—methicillin resistant *S. aureus*.

**Table 3:** Antifungal activity of compounds (μM × 10^−2^).

R.br		*A.f*	*A.v*	*A.o*	*A.n*	*T.v*	*P.o*	*P.f*	*Pvc*
**4a**	MIC	18.87	18.87	9.43	18.87	6.92	6.92	9.43	18.87
	MFC	44.03	37.74	18.87	37.74	9.43	18.87	18.87	37.74
**4b**	MIC	3.31	4.51	3.31	4.51	3.31	4.51	4.51	4.51
	MFC	4.51	9.03	4.51	9.03	4.51	9.03	9.03	9.03
**4c**	MIC	8.61	4.31	3.16	4.31	2.30	3.16	6.31	4.31
	MFC	17.22	8.61	4.31	8.61	4.31	4.31	8.61	8.61
**4d**	MIC	2.31	4.33	2.89	4.33	2.31	4.33	4.33	2.89
	MFC	4.33	8.66	4.33	8.66	4.33	8.66	8.66	4.33
**4e**	MIC	4.14	4.14	6.07	3.04	8.28	6.07	8.28	9.14
	MFC	8.28	8.28	8.28	4.14	16.57	8.28	16.57	17.08
**4f**	MIC	8.32	4.16	6.10	4.16	8.32	8.32	8.32	10.12
	MFC	16.64	8.32	8.32	8.32	16.64	16.64	16.64	19.76
**4g**	MIC	3.98	3.98	2.12	3.98	2.12	3.98	3.98	3.98
	MFC	7.97	7.97	3.98	7.97	3.98	7.97	7.97	7.97
**4h**	MIC	1.99	3.98	2.92	3.98	2.92	3.98	5.84	5.84
	MFC	3.98	7.97	3.98	7.97	3.98	7.97	7.97	7.97
**4i**	MIC	32.04	16.02	4.00	32.04	4.00	32.04	32.04	32.04
	MFC	64.09	32.04	8.01	64.09	8.01	64.09	64.09	64.09
**5a**	MIC	18.07	4.52	9.04	9.04	4.52	4.52	9.04	9.04
	MFC	36.14	9.04	18.07	18.07	9.04	9.04	18.07	18.07
**5b**	MIC	8.32	4.16	4.16	4.16	2.08	6.11	8.32	8.32
	MFC	16.67	8.32	8.32	8.32	4.16	8.32	16.67	16.67
**5c**	MIC	11.46	3.82	2.80	5.61	3.82	5.61	5.61	7.64
	MFC	15.29	7.64	3.82	7.64	7.64	7.64	7.64	15.29
**5d**	MIC	29.52	29.52	1.97	5.41	3.69	10.07	5.41	14.76
	MFC	59.04	59.04	3.69	7.38	7.38	14.76	7.38	29.52
**5e**	MIC	7.10	3.55	1.89	3.55	1.89	3.55	3.55	3.55
	MFC	14.20	7.10	3.55	7.10	3.55	7.10	7.10	7.10
**5f**	MIC	3.67	3.67	2.69	3.67	2.69	3.67	5.39	3.67
	MFC	7.34	7.34	3.67	7.34	3.67	3.67	3.67	3.67
**5g**	MIC	7.36	5.39	3.68	3.68	3.68	3.68	7.36	3.68
	MFC	14.72	7.36	7.36	7.36	7.36	7.36	14.72	7.36
**5h**	MIC	21.30	7.10	3.55	7.10	2.60	14.20	3.55	14.20
	MFC	28.40	14.20	7.10	14.20	3.55	28.40	7.10	28.40
**5i**	MIC	3.99	3.99	2.13	3.99	3.99	3.99	3.99	3.99
	MFC	7.97	7.97	3.99	7.97	7.97	7.97	7.97	7.97
**5j**	MIC	7.68	3.84	2.82	3.84	2.82	3.84	3.84	5.63
	MFC	15.37	7.68	3.84	7.68	3.84	7.68	7.68	7.68
**5k**	MIC	5.44	3.71	2.72	5.44	3.71	14.88	5.44	7.42
	MFC	7.42	7.42	3.71	7.42	7.42	29.67	7.42	14.88
Ketoconazole	MIC	38.0	285.0	38.0	38.00	475.0	38.00	380.0	37.60
	MFC	95.00	380.0	95.00	95.00	570.0	95.00	380.0	94.00
Bifonazole	MIC	48.00	48.0	48.00	48.00	64.00	64.00	48.00	32.20
	MFC	64.00	64.0	80.00	64.00	80.00	80.00	64.00	48.30

A.fum.—*A. fumigatus*, A.v.—*A. versicolor*, A.o.—*A. ochraceus*, A.n.—*A. niger*, T.v.—*T. viride*, P.f.—*P. funiculosum*, P.o.—*P. ochrochloron*, C.a.—*C. albicans*, P.v.c.—*P. cyclpoium* var verucosum. Relative standard deviations were all < 2.20.

**Table 4:** Molecular docking binding affinities on antibacterial targets.

Est. Binding Energy (kcal/mol)						
Comp.	Gyrase 1KZN	Thymidylate Kinase 4QGG	*E. coli* MurB 2Q85	*E. coli* MurB	1-H *E. coli* MurB	Residues *E. coli* MurB
**4a**	−1.28	-	−6.25	−23.74	1	Arg158
**4b**	−6.22	−2.69	−9.84	−30.42	2	Ser228
**4c**	−4.36	-	−7.15	−26.71	2	Arg158, Arg213
**4d**	−4.19	-	−8.10	−28.22	2	Arg158, Arg213
**4e**	−5.10	−2.41	−8.17	−28.36	2	Ser228, Arg213
**4f**	−4.07	-	−7.11	−26.55	2	Arg158, Arg213
**4g**	−6.25	−3.14	−10.08	−31.16	2	Gly122, Ser228
**4h**	−7.09	−4.66	−11.25	−33.49	3	Arg158, Ser228, Asn232
**4i**	−5.28	-	−7.70	−27.11	2	Tyr189, Ser228
**5a**	−3.65	−3.27	−6.88	−24.79	2	Arg158, Tyr189
**5b**	−7.15	−4.19	−12.33	−36.27	3	Ser228, Ala226
**5c**	−5.13	−2.26	−8.25	−28.41	2	Ser228, Arg213
**5d**	−6.92	−3.27	−10.51	−31.44	2	Gly122, Ser228
**5e**	−6.20	-	−9.82	−30.71	2	Ser228, Ala226
**5f**	−4.32	-	−7.14	−26.58	2	Arg158, Arg213
**5g**	−7.00	−4.11	−11.28	−33.42	3	Arg158, Ser228, Asn232
**5h**	−5.87	−3.15	−8.75	−28.98	2	Gly122, Ser228
**5i**	-	-	−5.73	−20.75	-	-
**5j**	−2.55	-	−5.77	−20.86	1	Arg213
**5k**	−5.84	−3.11	−8.67	−28.33	2	Gly122, Ser228

**Table 5:** Molecular docking binding affinities for antifungal targets.

No	Est. Binding Energy (kcal/mol) CYP51 of *C. albicans* PDB ID: 5V5Z	Binding Affinity Score CYP51 of *C. albicans* PDB ID: 5V5Z	I-H	Residues CYP51 of *C. albicans* PDB ID: 5V5Z
**17 4a**	−3.15	−15.18	-	-
**4 4b**	−8.14	−27.22	1	Tyr132
**13 4c**	−7.15	−26.02	1	Tyr132
**5 4d**	−10.89	−31.08	-	HEM601 (ionizable)
**14 4e**	−4.18	−15.21	-	
**2 4i**	−3.15	−13.57	-	-
**12 4g**	−9.66	−29.47	1	Tyr132
**15 4h**	−10.14	−30.25	-	HEM601 (ionizable)
**2 4i**	−3.15	−13.57	-	-
**18 5a**	−5.12	−20.96	-	-
**19 5b**	−1.14	−6.29	-	-
**7 5c**	−5.16	−20.85	1	Tyr118
**11 5d**	−5.17	−21.30	-	-
**8 5f**	−11.13	−32.56	1	Tyr132 HEM601 (ionizable, pi)
**1 5h**	−6.68	−24.79	-	-
**3 5i**	−8.74	−27.58	1	Tyr64
**9 5j**	−8.14	−26.97	1	Tyr64
**10 5k**	−6.25	−23.88	1	Tyr118

## Supplementary Materials

The following are available online.

## References

[1] Nii-Trebi N.I. (2017). Emerging and neglected infectious diseases: Insights, advances, and challenges. Biomed. Res. Int..

[2] Livorsi D.J., Stenehjem E., Stephens D.S., Herwald H., Egesten A. (2011). Sepsis -Pro-Inflammatory and Anti-Inflammatory Responses.

[3] Shuvankar Mukherjee S. (2017). Emerging infectious diseases: Epidemiological perspective. Indian J. Dermatol..

[4] Holmes A., Moore L., Sundsfjord A., Steinbakk M., Regmi S., Karkey A., Guerin P., Piddock L. (2016). Understanding the mechanisms and drivers of antimicrobial resistance. Lancet.

[5] Winter S.E., Lopez C.A., Bäumler A.J. (2013). The dynamics of gut-associated microbial communities during inflammation. EMBO Rep..

[6] Payne D.J., Gwynn M.N., Holmes D.J., Pompliano D.L. (2007). Drugs for bad bugs: Confronting the challenges of antibacterial. Nat. Rev. Drug Discov..

[7] He X.Y., Lu L., Qiu J., Zou P., Yu F., Jiang X.K., Li L., Jiang S., Liu S., Xie L. (2013). Small molecule fusion inhibitors: Design, synthesis and biological evaluation of (*Z*)-3-(5-(3-benzyl-4-oxo-2-thioxothiazolidinylidene)methyl)-*N*-(3-carboxy-4-hydroxy)phe-nyl-2,5-dimethylpyrroles and related derivatives targeting HIV-1gp41. Bioorg. Med. Chem..

[8] Yingchoncharoen P., Kalinowski D.S., Richardson D.R. (2016). Lipid-baseddrug delivery systems in cancer therapy: What is available andwhat is yet to come. Pharmacol. Rev..

[9] Nitsche C., Schreier V.N., Behnam M.A.M. (2013). Thiazolidinone–peptide hybrids as dengue virus protease inhibi-tors with antiviral activity in cell culture. J. Med. Chem..

[10] Bari S.B., Firake S.D. (2016). Exploring anti-inflammatory Potential of thiazolidinone derivatives of benzenesulfonamide via synthesis, molecular docking and biological evaluation. Anti-Inflamm. Anti-Allergy Agents Med. Chem..

[11] Khaled R.A., Mohamed A.A., Heba A.H., Shahinda S.R. (2016). Design, synthesis and biological screening of new 4-thiazolidinone derivatives with promising COX-2 selectivity, anti-inflammatory activity and gastric safety profile. Bioorg. Chem..

[12] Yasmin S., Capone F., Laghezza A., Dal Piaz F., Loiodice F., Vijayan V., Devadasan V., Mondal S., Atlı O., Baysal M. (2017). Novel benzylidene thiazolidinedione derivatives as partial PPARγ agonists and their antidiabetic effects on type 2 diabetes. Sci. Rep..

[13] Djukic M., Fesatidou M., Xenikakis I., Geronikaki A., Angelova V., Savic V., Pasic M., Krilovic B., Djukic D., Gobeljic B. (2018). In vitro antioxidant activity of thiazolidinone derivatives of 1,3-thiazole and 1,3,4-thiadiazole. Chem. Biol. Interact..

[14] Shafii N., Khoobi M., Amini M. (2015). Synthesis and biologicalevaluation of 5-benzylidenerhodanine-3-acetic acid derivatives asAChE and 15-LOX inhibitors. J. Enzym. Inhib. Med. Chem..

[15] Kratky M., Stepankova S., Vorcakova K., Vinšová J. (2016). Synthesis and invitro evaluation of novel rhodanine derivatives as potential cho-linesterase inhibitors. Bioorg. Chem..

[16] El-Sayed S., Metwally K., El-Shanawani A.A., Abdel-Aziz L.M., El-Rashedy A.A., Soliman M.E.S., Quattrini L., Coviello V., la Motta C. (2017). Quinazolinone-based rhodanine-3-acetic acids as potent aldose reductase inhibitors: Synthesis, functional evaluation and molecular modeling study. Bioorg. Med. Chem. Lett..

[17] Sim M.M., Ng S.B., Buss A.D. (2002). Benzylidene rhodanines as novel inhibitors of UDP-N-acetylmuramate/l-alanine ligase. Bioorg. Med. Chem. Lett..

[18] Miao J., Zheng C.-J., Sun L.-P., Song M.-X., Xu L.-L., Piao H.-R. (2013). Synthesis and potential anti-bacterial activity of new rhodanine-3-acetic acid derivatives. Med. Chem. Res..

[19] Gupta A., Singh R., Sonar P.K., Saraf S.K. (2016). Novel 4-thiazolidinone deriva-tives as anti-infective agents: Synthesis, characterization, andantimicrobial evaluation. Biochem. Res. Int..

[20] Tejchman W., Korona-Glowniak I., Malm A., Zylewski M., Suder P. (2017). Antibacterialproperties of 5-substituted derivatives of rhodanine-3-carbox-yalkyl acids. Med. Chem. Res..

[21] Song M.X., Zheng C.J., Deng X.Q., Wei Z.-Y., Piao H.-R. (2014). The synthesis and anti-bacterial activities of N-carboxymethyl rhodanines. Med. Chem..

[22] Krátký M., Vinšová J., Stolaříková J. (2017). Antimicrobial activity of rhodanine-3-acetic acid derivatives. Bioorg. Med. Chem..

[23] Zheng C.J., Song M.X., Sun L.P., Wu Y., Hong L., Piao H.R. (2012). Synthesis and biological evalution of 5-aryloxypyrazole derivatives bearing a rhodanine-3-aromatic acid as potential antimicrobial agents. Bioorg. Med. Chem. Lett..

[24] Li W., Zhai X., Zhong Z., Li G., Pu Y., Gong P. (2011). Design, synthesis and evaluation of novel rhodanine-containing sorafenib analogs as potential antitumor agents. Arch. Pharm. (Weinheim).

[25] Lafayette E.A., de Almeida S.M.V., Santos R.V.C., de Oliveira J.F., da Cruz Amorim C.A., da Silva R.M.F., da Rocha Pitta M.G., da Rocha Pitta I., de Moura R.O., de Carvalho Junior L.B. (2017). Synthesis of novel indole derivatives as promising DNA-binding agents and evaluation of antitumor and antitopoisomerase I activities. Eur. J. Med. Chem..

[26] Villain-Guillot P., Gualtieri M., Bastide L., Roquet F., Martinez J., Amblard M., Pugniere M., Leonetti J.P. (2007). Structure-activity relationships of phenyl-furanyl-rhodanines as inhibitors of RNA polymerase with antibacterial activity on biofilms. J. Med. Chem..

[27] Song M.X., Li S.H., Peng J.Y., Guo T.T., Xu W.H., Xiong S.F., Deng X.Q. (2017). Synthesis and bioactivity evaluation of *N*-arylsulfonylindole analogs bearing a rhodanine moiety as antibacterial agents. Molecules.

[28] Johnson S.L., Chen L., Harbach R., Sabet M., Savinov A., Cotton N.J., Strongin A., Guiney D., Pellecchia M. (2008). Rhodanine derivatives as selective protease inhibitors against bacterial toxins. Chem. Biol. Drug Des..

[29] Bataille C.J., Brennan M.B., Byrne S., Davies S.G., Durbin M., Fedorov O., Huber K., Jones A., Knapp S., Liu G. (2017). Thiazolidine derivatives as potent and selective inhibitors of the PIM kinase family. Bioorg. Med. Chem..

[30] Pinson J.A., Schmidt-Kittler O., Zhu J., Jennings I.G., Kinzler K.W., Vogelstein B., Chalmers D.K., Thompson P.E. (2011). Thiazolidinedione-based PI3Kα inhibitors: An Analysis of biochemical and virtual screening methods. Chem. Med. Chem..

[31] Song H., Lee Y.S., Roh E.J., Seo J.H., Oh K.S., Lee B.H., Han H., Shin K.J. (2012). Discovery of potent and selective rhodanine type IKKβ inhibitors by hit-to-lead strategy. Bioorg. Med. Chem. Lett..

[32] Sukanta K., Edward B.R. (2012). Microwave-assisted synthesis of novel bis(2-thioxothiazolidin-4-one) derivatives as potential GSK-3 inhibitors. Tetrahedron Lett..

[33] Parrino B., Diana P., Cirrincione G., Casciofero S. (2018). Bacterial biofilm inhibition in the development of effective anti-virulence strategy. Open Med. Chem. J..

[34] Kostić M., Smiljković M., Petrović J., Glamočilija J., Barros L., Ferreira I.C.F.R., Ćirić A., Soković M. (2017). Chemical, nutritive composition and a wide range of bioactive properties of honey mushroom Armillaria mellea (Vahl: Fr.) Kummer. Food Funct..

[35] Clinical and Laboratory Standards Institute (2009). Methods for Dilution Antimicrobial Susceptibility Tests for Bacteria that Grow Aerobically. Approved Standard.

[36] Fesatidou M., Zagaliotis P., Camoutsis C., Petrou A., Eleftheriou P., Tratrat C., Haroun M., Geronikaki A., Ciric A., Sokovic M. (2018). 5-Adamantan thiadiazole- based thiazolidinones as antimicrobial agents. Design, synthesis, molecular docking and evaluation. Bioorg. Med. Chem..

[37] Kartsev V., Geronikak A., Petrou A., Lichitsky B., Kostic M., Smiljkovic M., Soković M., Sirakanyan S. (2019). Griseofulvin derivatives: Synthesis, molecular docking and biological evaluation. Curr. Top. Med. Chem..

[38] Espinel-Ingroff A. (2001). Comparison of the E-test with the NCCLS M38-P method for antifungal susceptibility testing of common and emerging pathogenic filamentous fungi. J. Clin. Microb..

[39] Hanel H., Raether W. (1988). A More Sophisticated Method of Determining the Fungicidal Effect of Water-Insoluble Preparations with a Cell Harvester, Using Miconazole as an Example. MYCOSES.

[40] Drenkard E., Ausubel F.M. (2002). Pseudomonas biofilm formationand antibiotic resistance are linked to phenotypic variation. Nature.

