# Supplementary material for: 5-(1H-Indol-3-ylmethylene)-4-oxo-2-thioxothiazolidin-3-yl)alkancarboxylic Acids as Antimicrobial Agents: Synthesis, Biological Evaluation, and Molecular Docking Studies
Source: Molecules. 2020 Apr 23;25(8):1964. doi: 10.3390/molecules25081964 (PMC7221690; doi:10.3390/molecules25081964)
Supplement: Supplementary file 1 [file molecules-25-01964-s001.pdf]

5-(1*H*-Indole-3-ylmethylen)-4-oxo-2-thioxothiazolidin -3-yl)alkancarboxylic acids as antimicrobial agents. Synthesis, biological evaluation and molecular docking studies.

Horishny V.Ya.[1,] Kartsev V.G.[2]., Matiychuk V.S.[3], Geronikaki A[4]., Petrou [4]., Glamoclija J.[5], Ciric A.[5], Sokovic M.[5]

[1] *Danylo Halytsky Lviv National Medical University, 79010, Ukraine, Lviv,.*

Pekarska 69

[2] *InterBioScreen, Moscow, Russia*

43Aristotle University, School of Pharmacy, Thessaloniki, Greece

[3] *Ivan Franko National University of Lviv, 79005, Ukraine, Lviv,.*

Kyryla i Mefodia 6.

[4]*Aristotle University of Thessaloniki, School of Pharmacy, Thessaloniki, 54124, Greece.*

[4].*Mycological Laboratory , Institute of Biological Research Sinisa Stankovic, Belgrade University, Serbia*

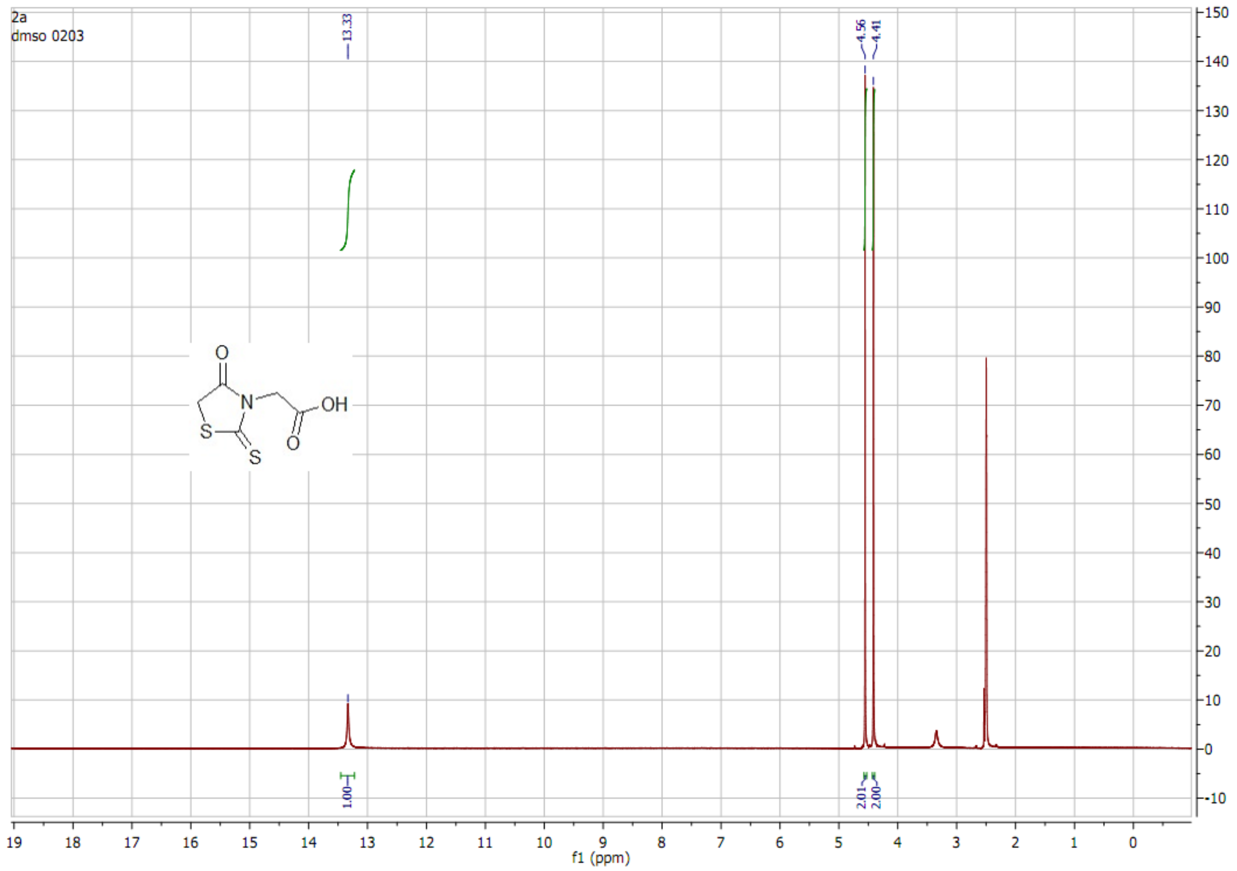

Figure S1. ^{1}HNMR spectrum (400 MHz, DMSO-d$_6$) of compound **2a**.

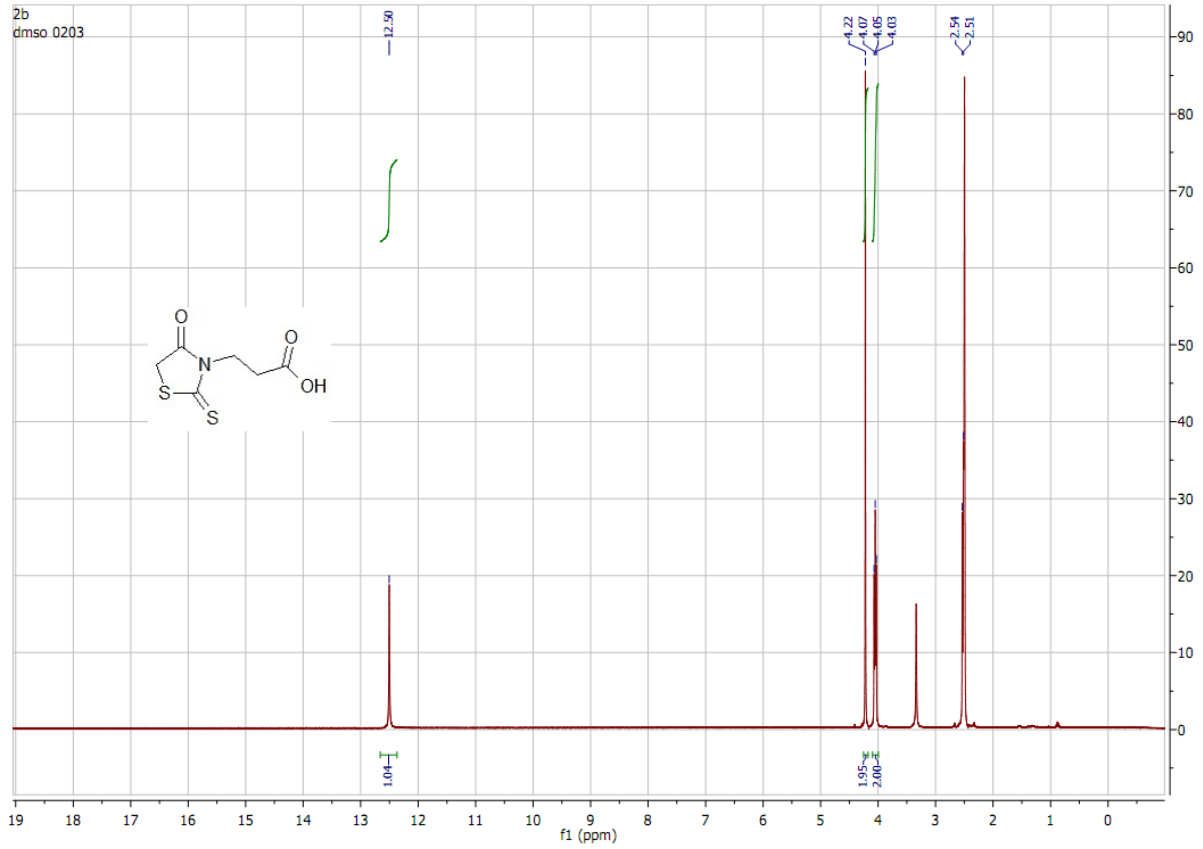

Figure S2. ^{1}HNMR spectrum (400 MHz, DMSO-d$_6$) of compound **2b**.

Figure S3. ^{1}HNMR spectrum (400 MHz, DMSO-d_6) of compound **2c**.

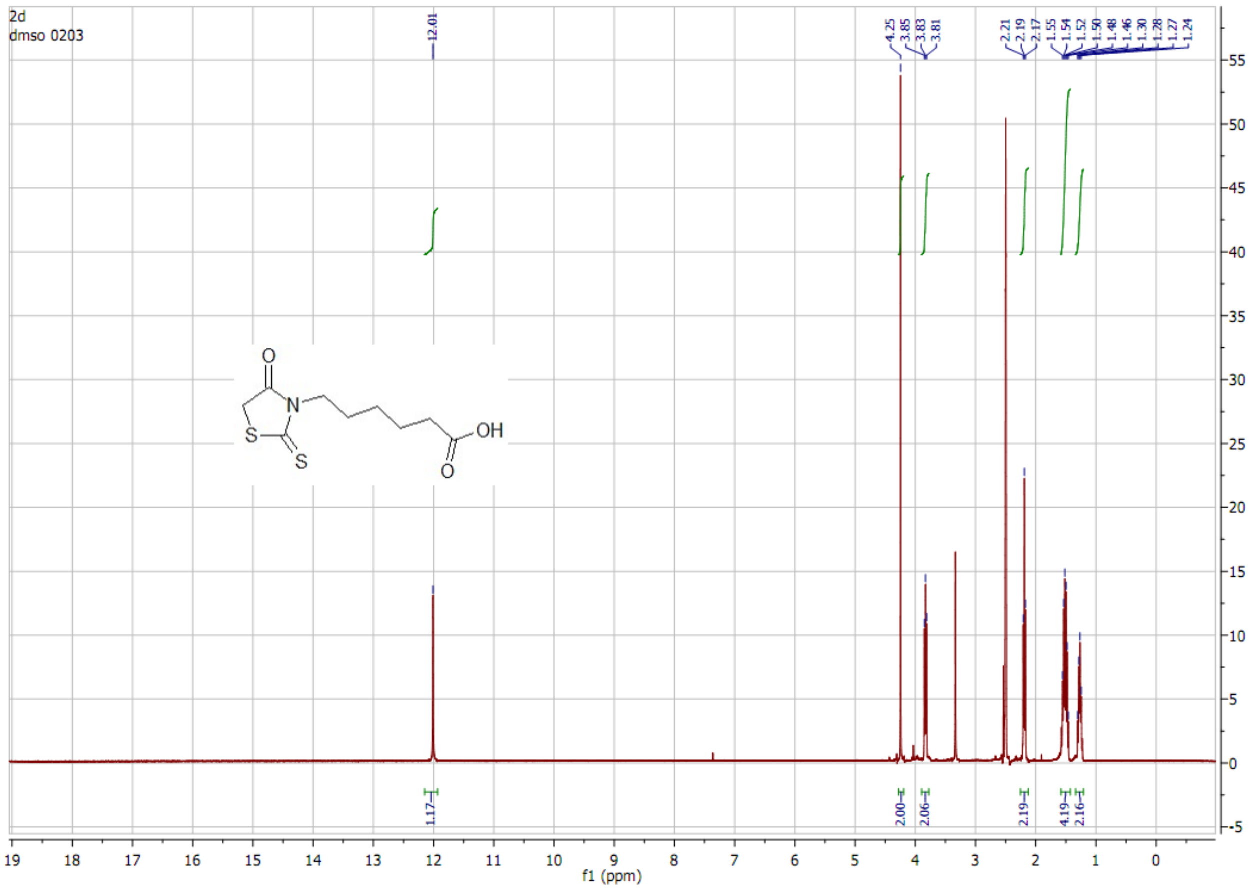

Figure S4. ^{1}HNMR spectrum (400 MHz, DMSO-d_6) of compound **2d**.

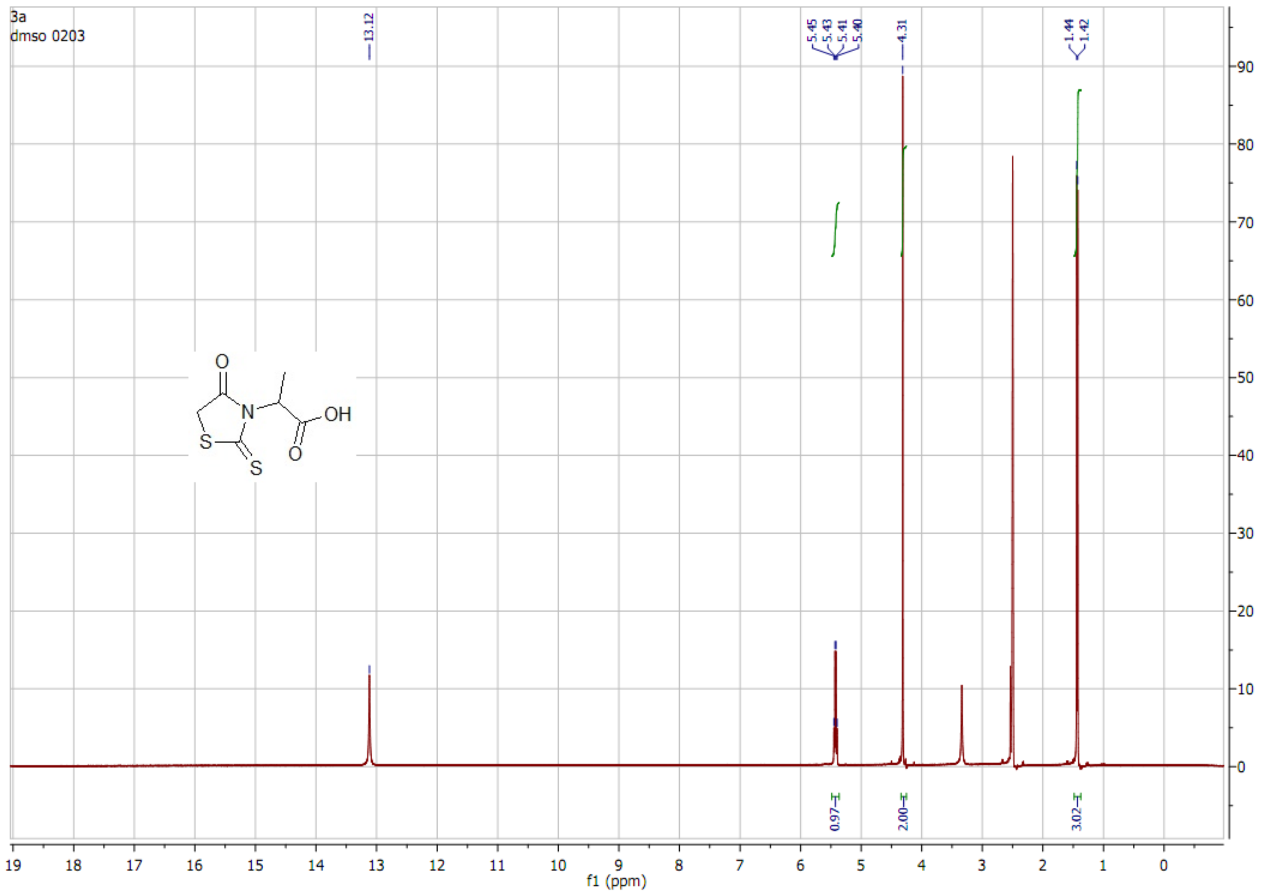

Figure S5. ^{1}HNMR spectrum (400 MHz, DMSO-d_6) of compound **3a**.

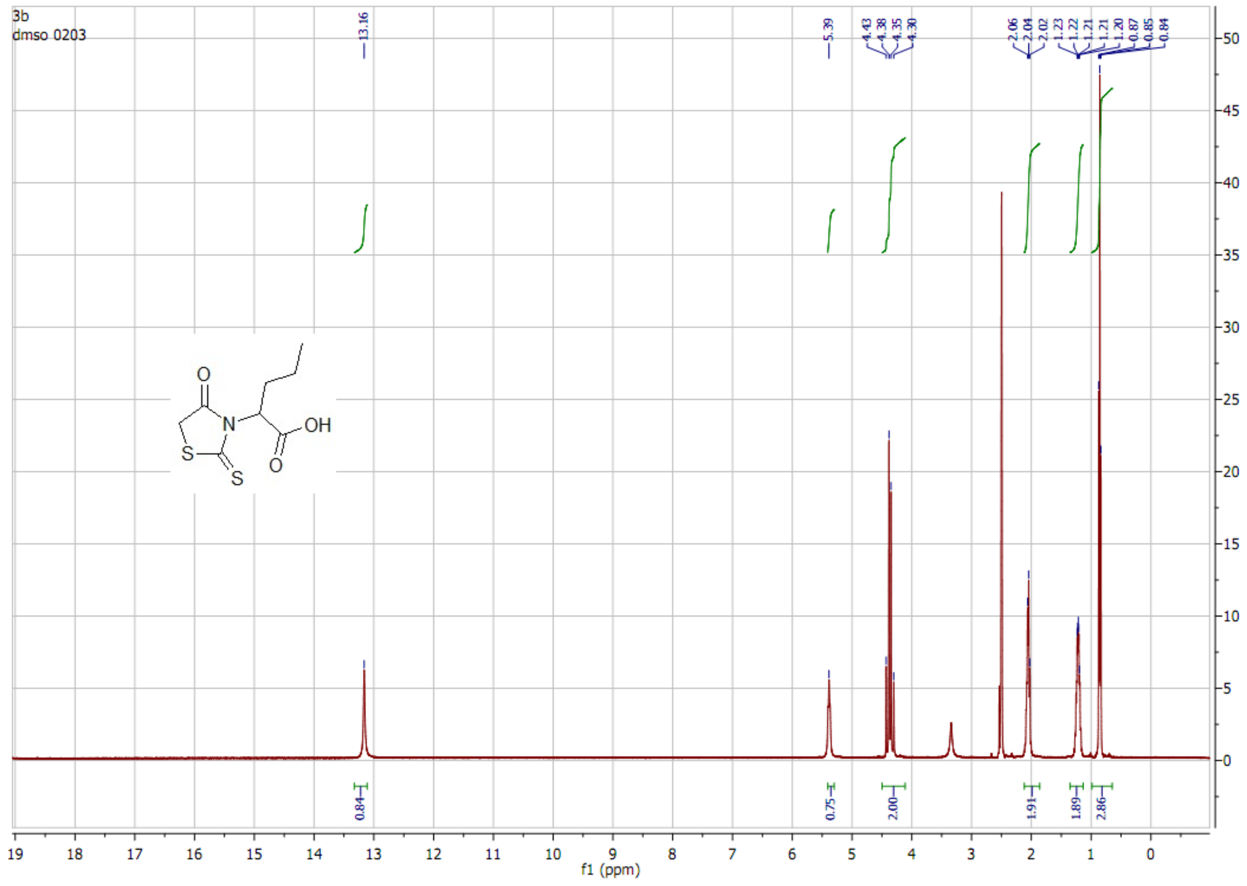

Figure S6. ^{1}HNMR spectrum (400 MHz, DMSO-d_6) of compound **3b**.

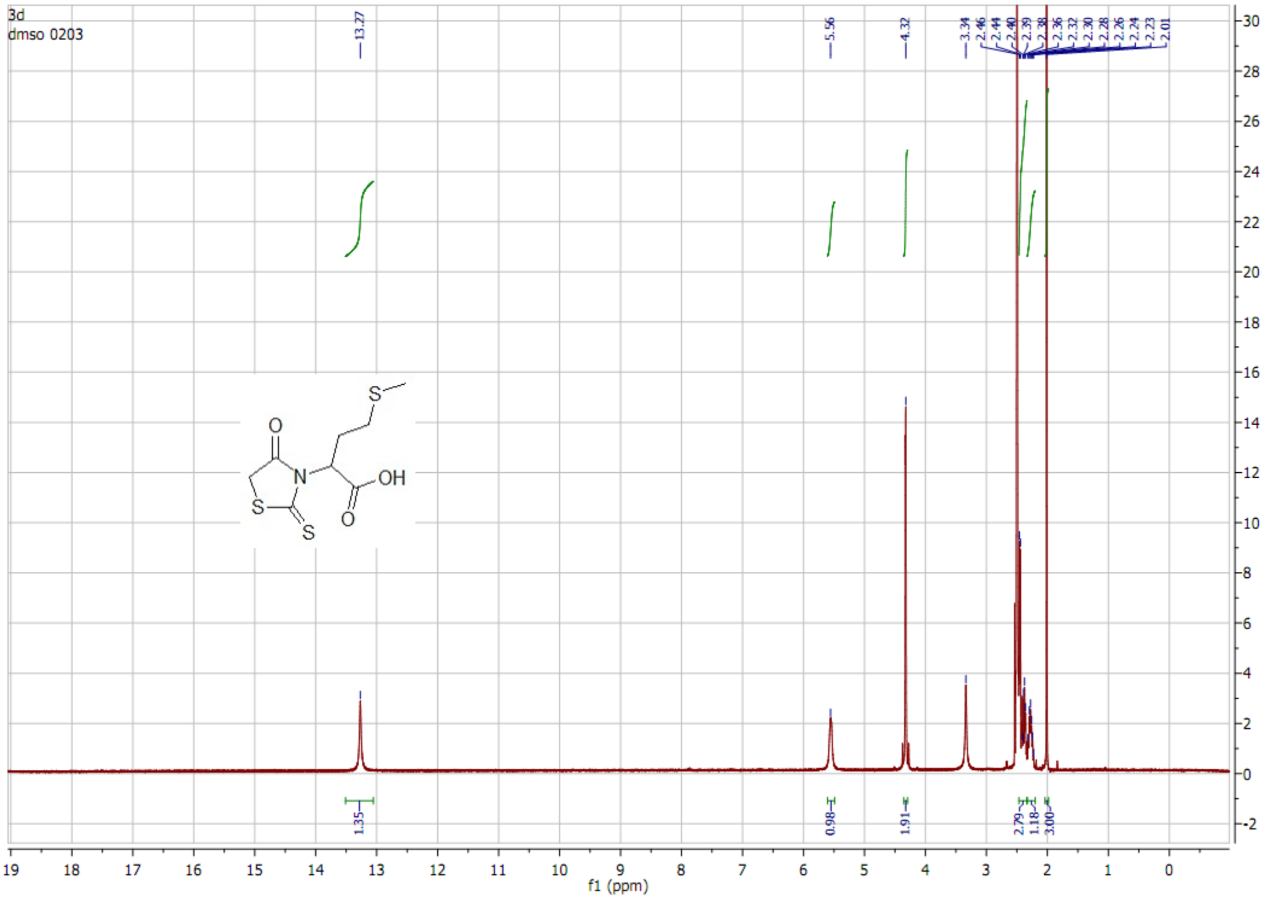

Figure S7. ^{1}HNMR spectrum (400 MHz, DMSO-d_6) of compound **3c**.

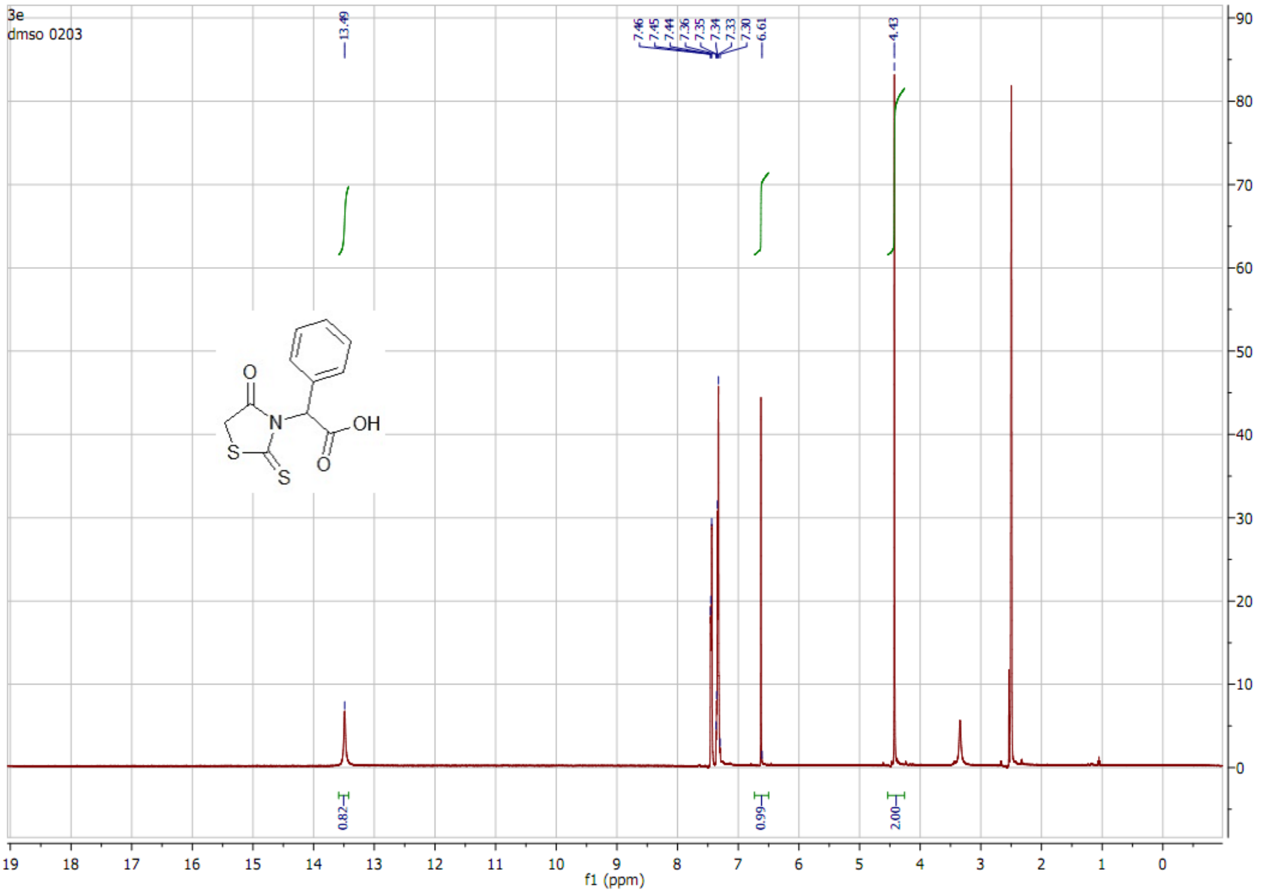

Figure S8. ^{1}HNMR spectrum (400 MHz, DMSO-d_6) of compound **3d**.

Figure S9. ^{1}HNMR spectrum (400 MHz, DMSO-d_6) of compound **3e**

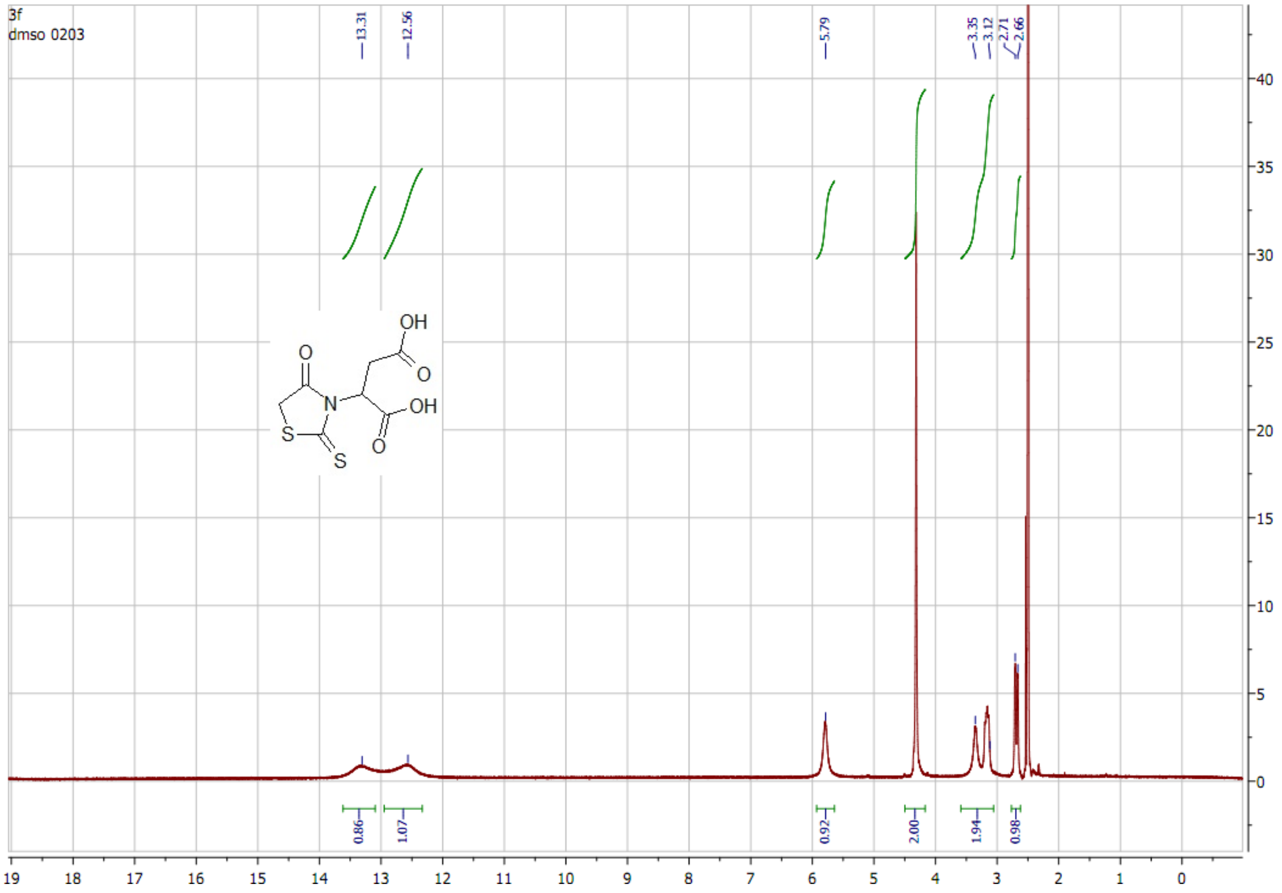

Figure S10. ^{1}HNMR spectrum (400 MHz, DMSO-d_6) of compound **3f**

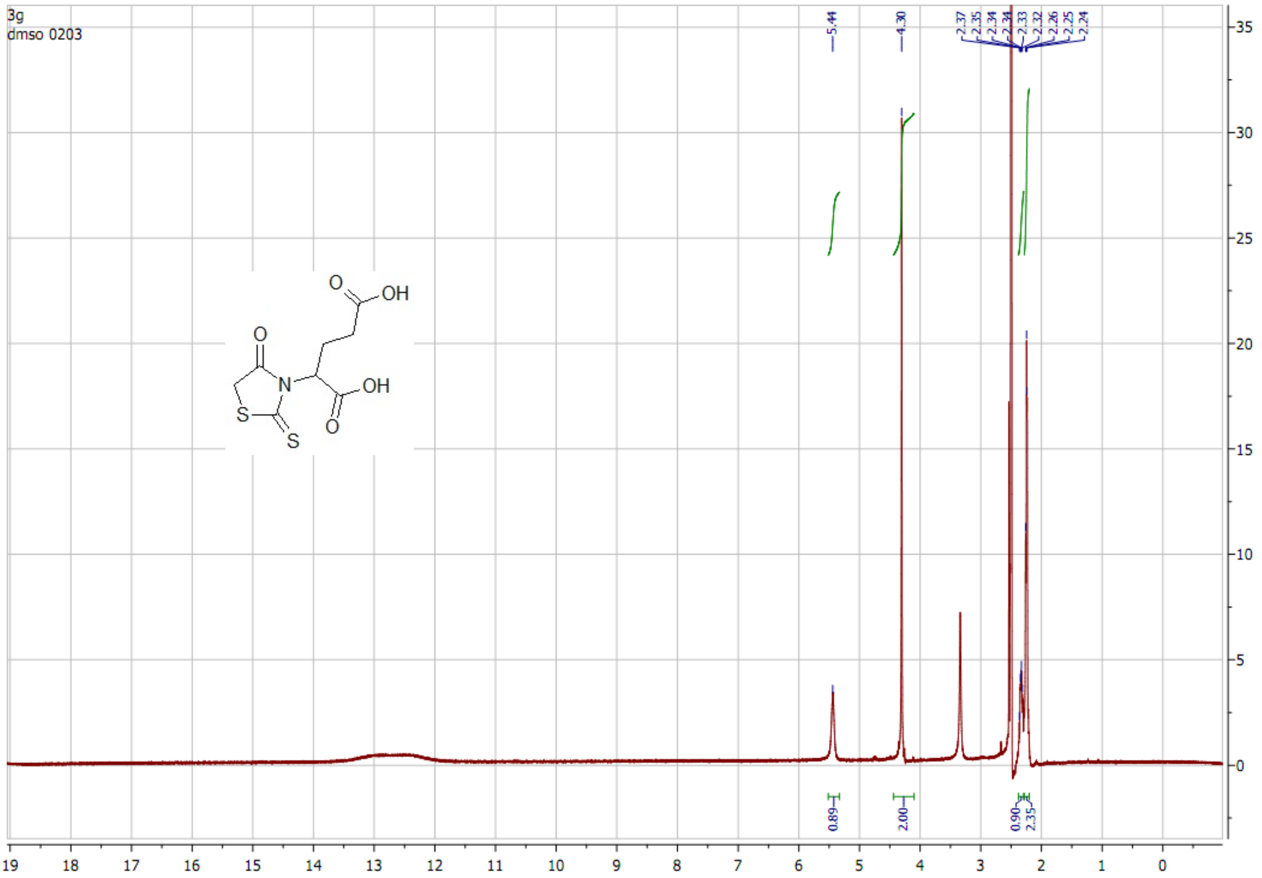

. **Figure S11**. ^{1}HNMR spectrum (400 MHz, DMSO-d_6) of compound **3g**

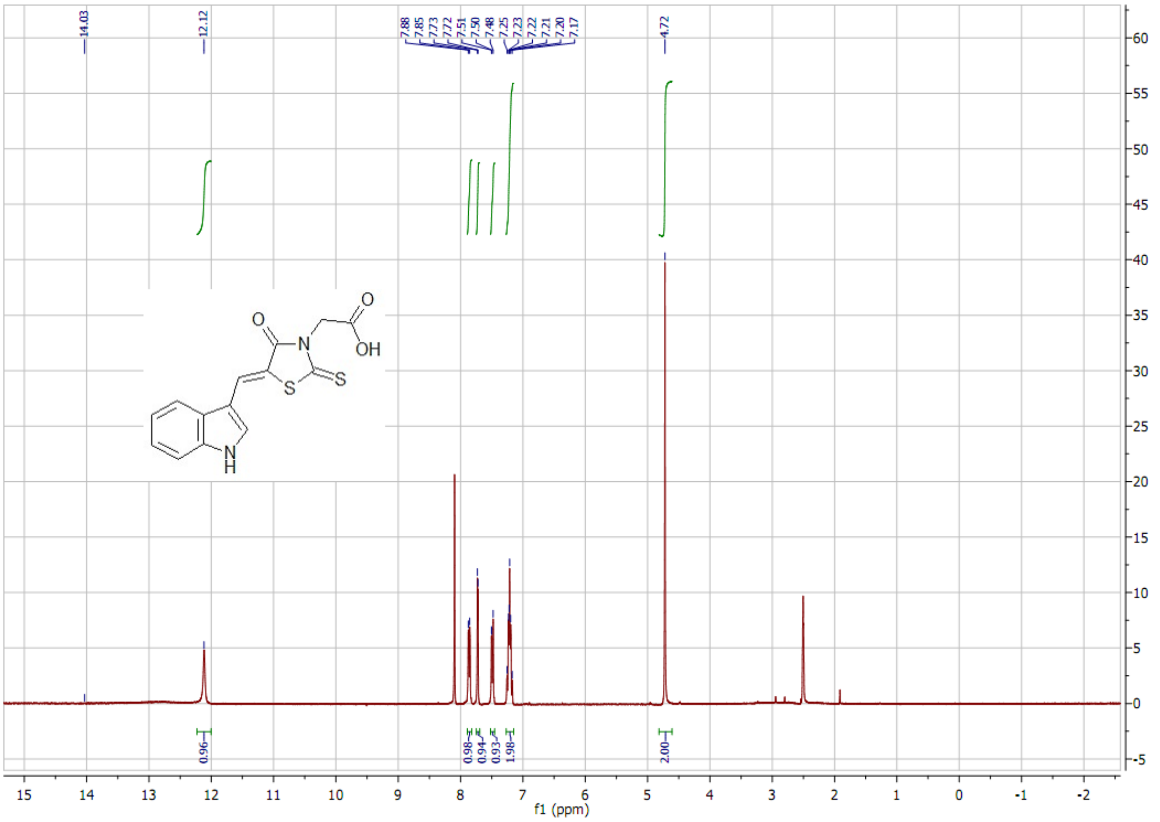

Figure S12. ^{1}HNMR spectrum (300 MHz, DMSO-d_6) of compound **4a**.

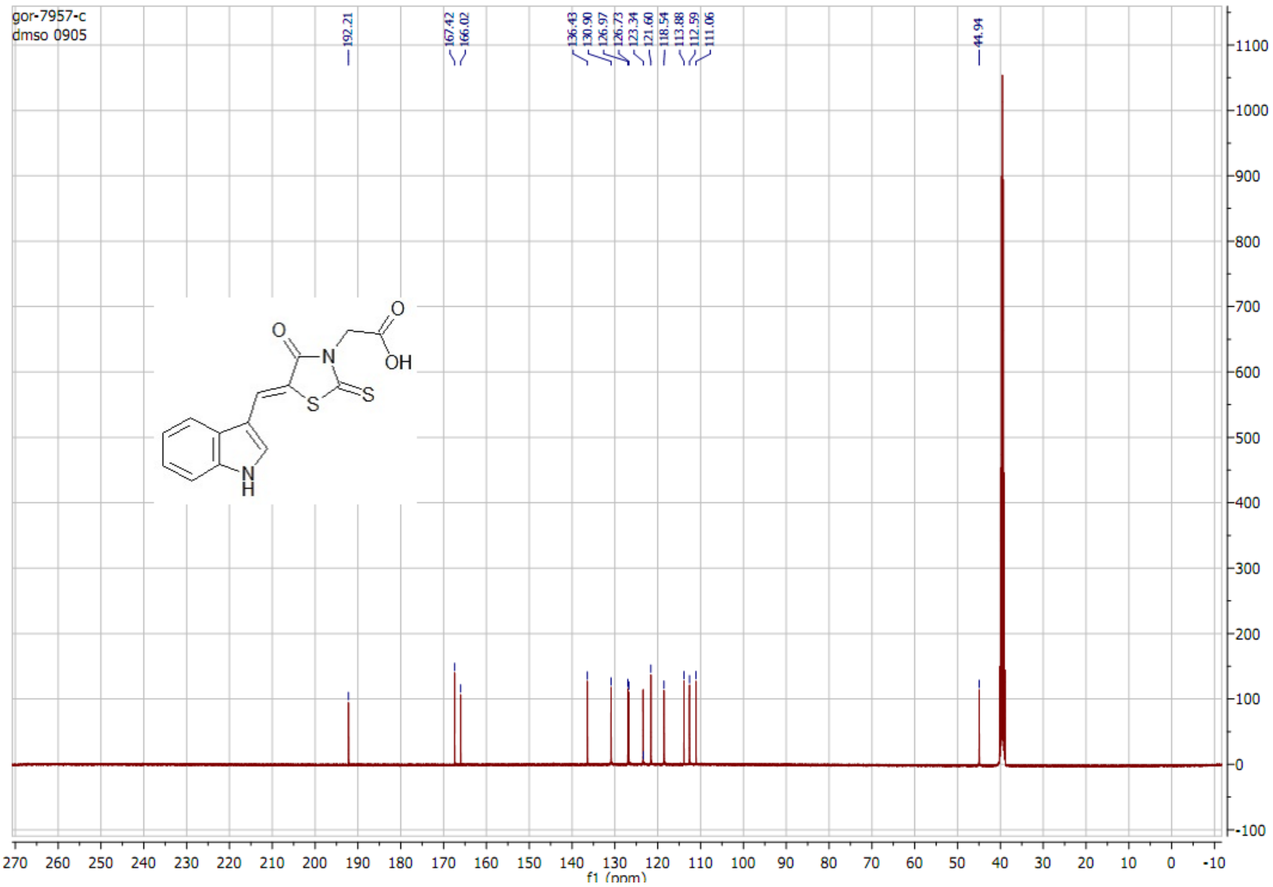

Figure S13. ^{13}C NMR spectrum (101 MHz, DMSO-d_6) of compound **4a**

Sample Name: [illegible]

-.o.-Syntez Purity Report -.o.-

System: Agilent 1200 LC/MSD SL
Separation column: Rapid Resolutionn HT Cartige 4.6x30mm, 1.8-Micron, Zorbx SB-C18

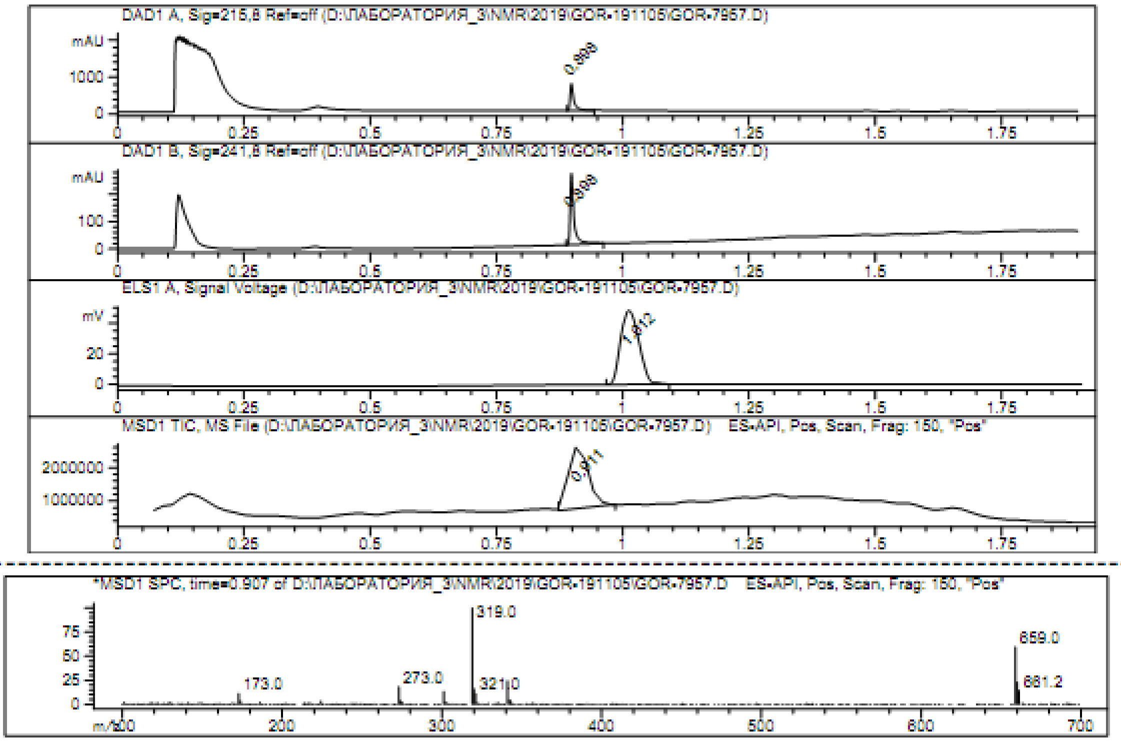

#	Signal	R.Time	Area %
1	DAD1 A, Sig=215,8 Ref=off	0.898	100.000

#	Signal	R.Time	Area %
1	DAD1 B, Sig=241,8 Ref=off	0.898	100.000

#	Signal	R.Time	Area %
1	ELS1 A, Signal Voltage	1.012	100.000

#	Signal	R.Time	Area %
1	MSD1 TIC, MS File	0.911	100.000

Inj. Data 05.11.2019

Figure S14. MS (ESI) spectrum of compound **4a.**

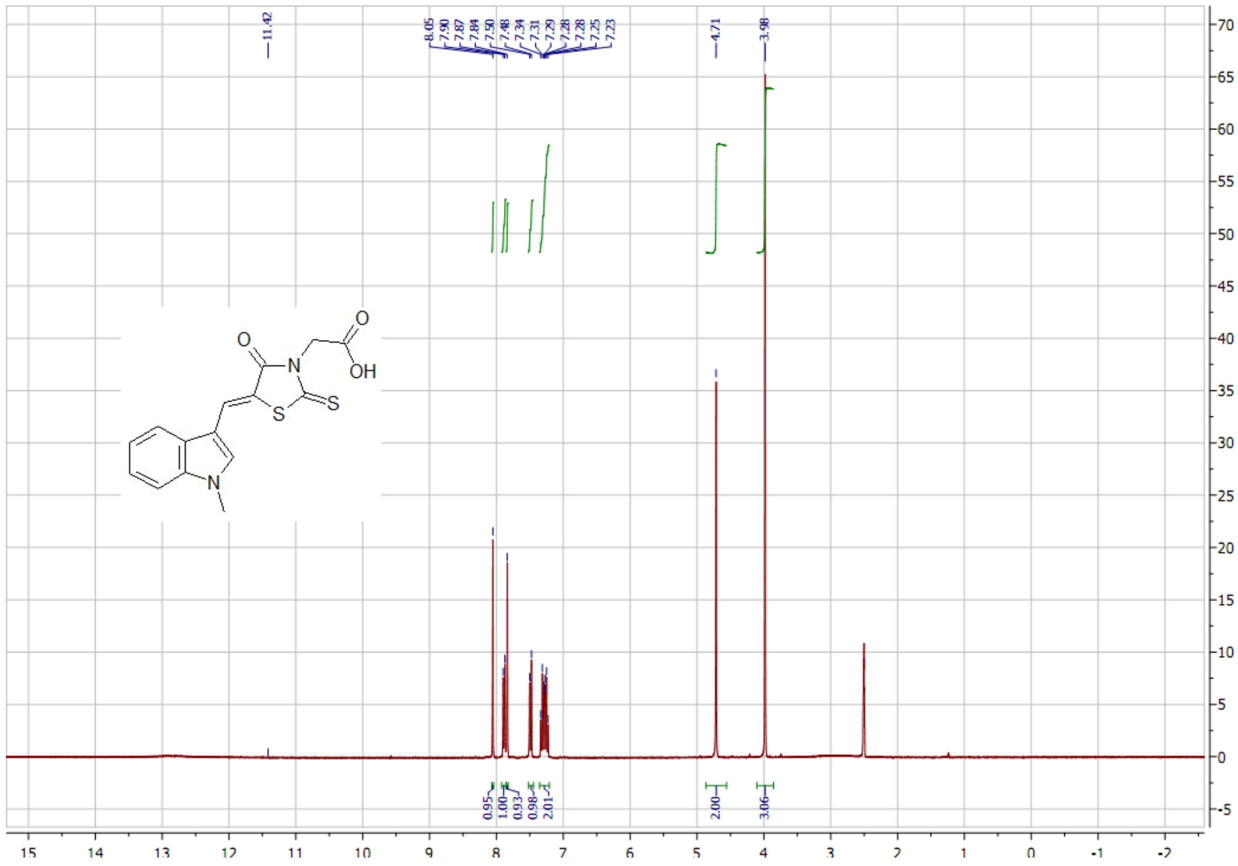

Figure S15. ^{1}HNMR spectrum (300 MHz, DMSO-d_6) of compound **4b**.

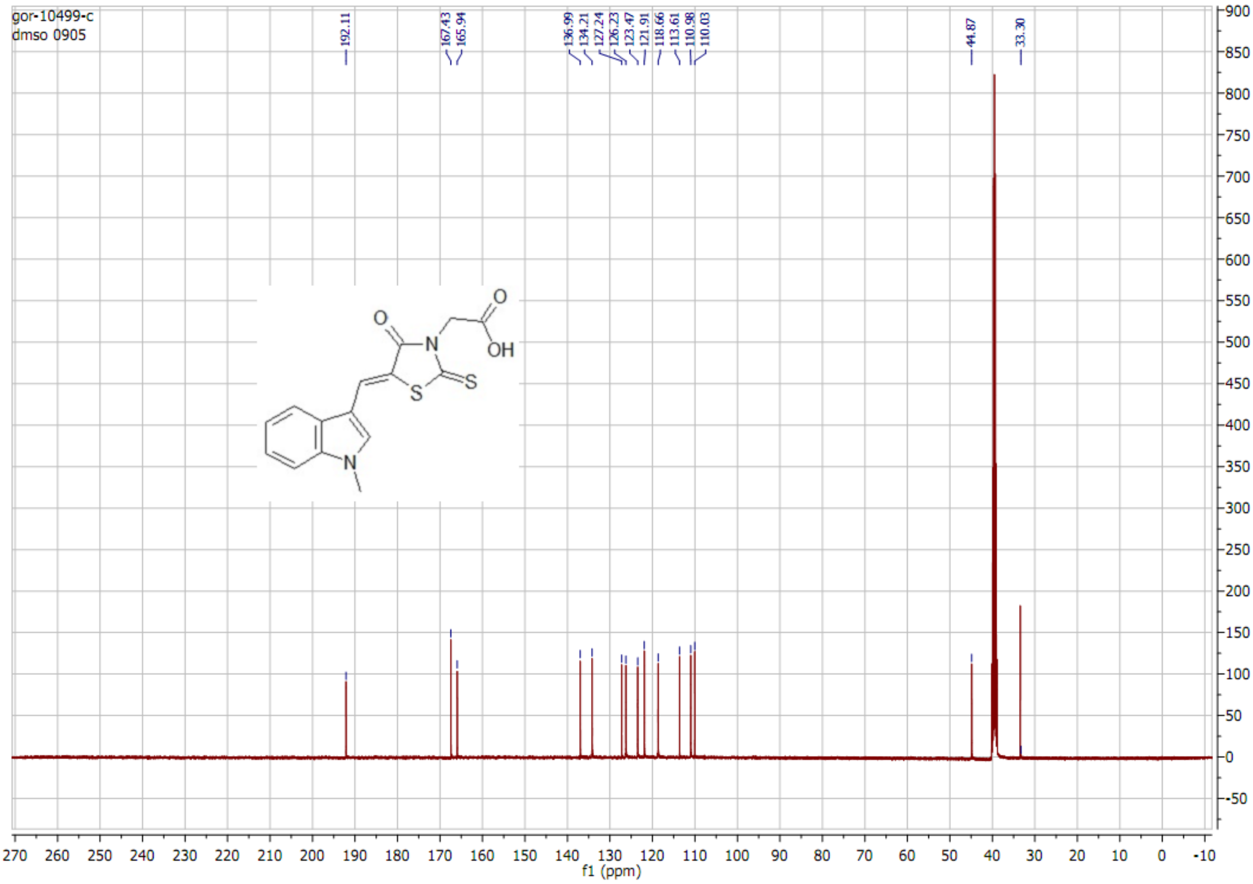

Figure S16. ^{13}C NMR spectrum (101 MHz, DMSO-d_6) of compound **4b**

-.o.-Syntez Purity Report -.o.-

System: Agilent 1200 LC/MSD SL
Separation column: Rapid Resolutionn HT Cartige 4.6x30mm, 1.8-Micron, Zorbx SB-C18

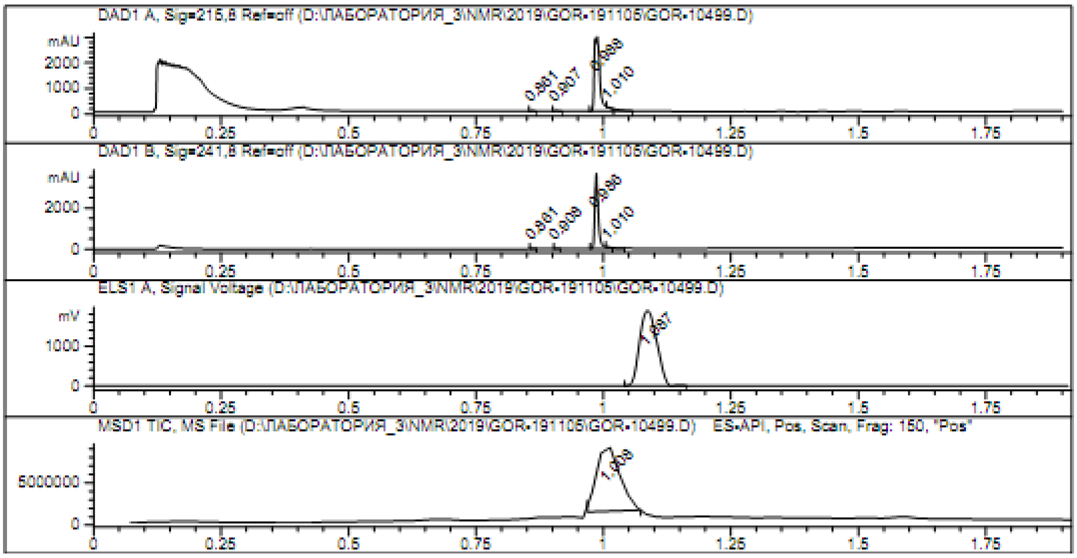

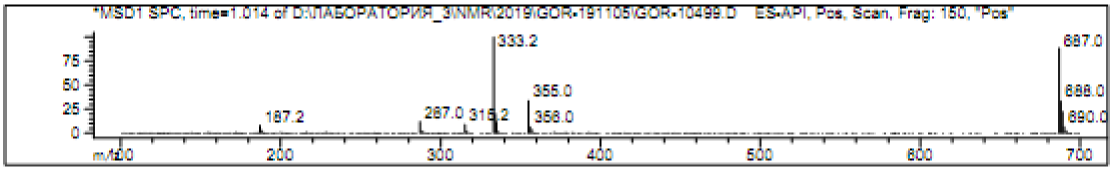

#	Signal	R.Time	Area %
1	DAD1 A, Sig=215,8 Ref=off	0.861	0.187
2		0.907	0.508
3		0.988	99.236
4		1.010	0.069

#	Signal	R.Time	Area %
1	DAD1 B, Sig=241,8 Ref=off	0.861	0.311
2		0.908	0.340
3		0.986	98.967
4		1.010	0.382

#	Signal	R.Time	Area %
1	ELS1 A, Signal Voltage	1.087	100.000

#	Signal	R.Time	Area %
1	MSD1 TIC, MS File	1.008	100.000

Inj. Data 06.11.2019

Figure S17. MS (ESI) spectrum of compound **4b.**

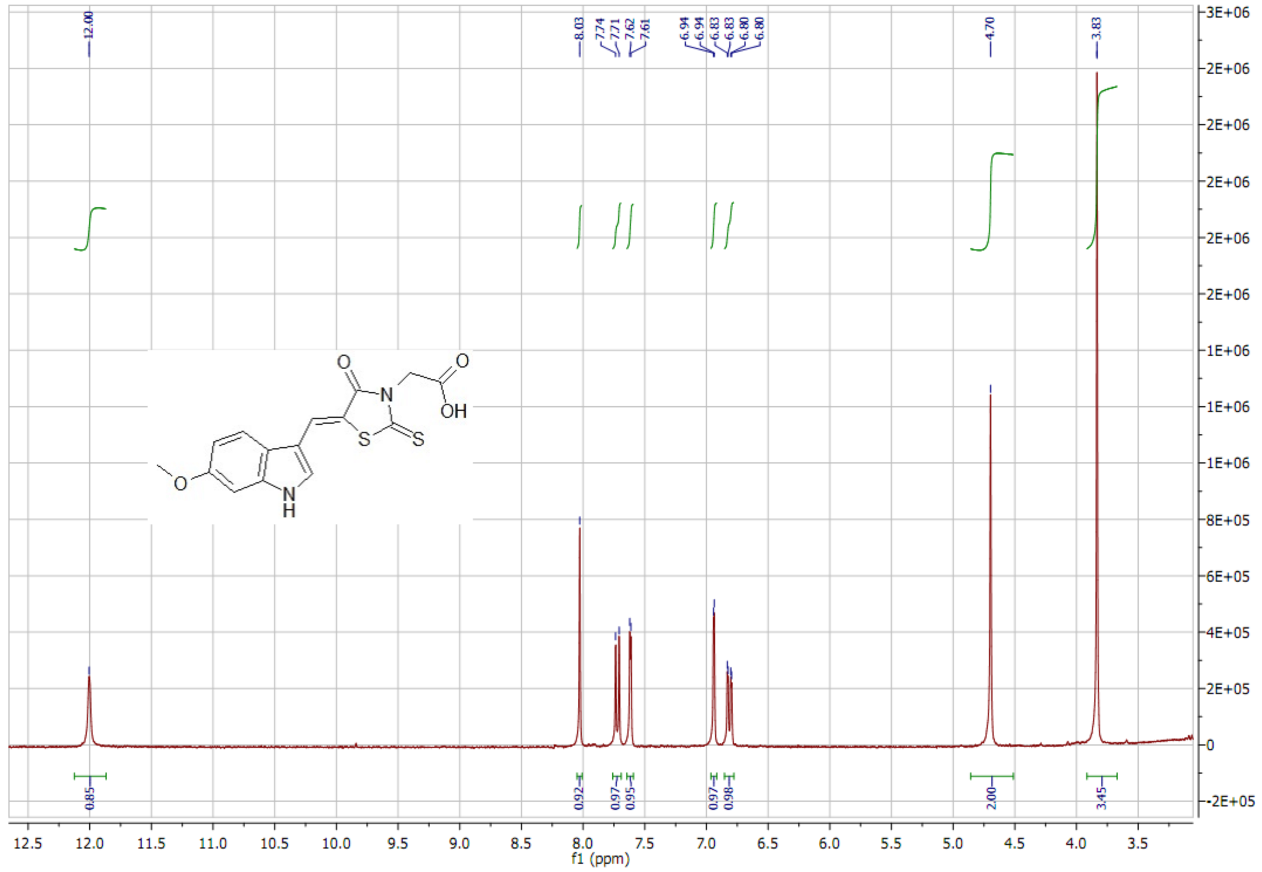

Figure S18. ^{1}HNMR spectrum (300 MHz, DMSO-d_6) of compound **4c**.

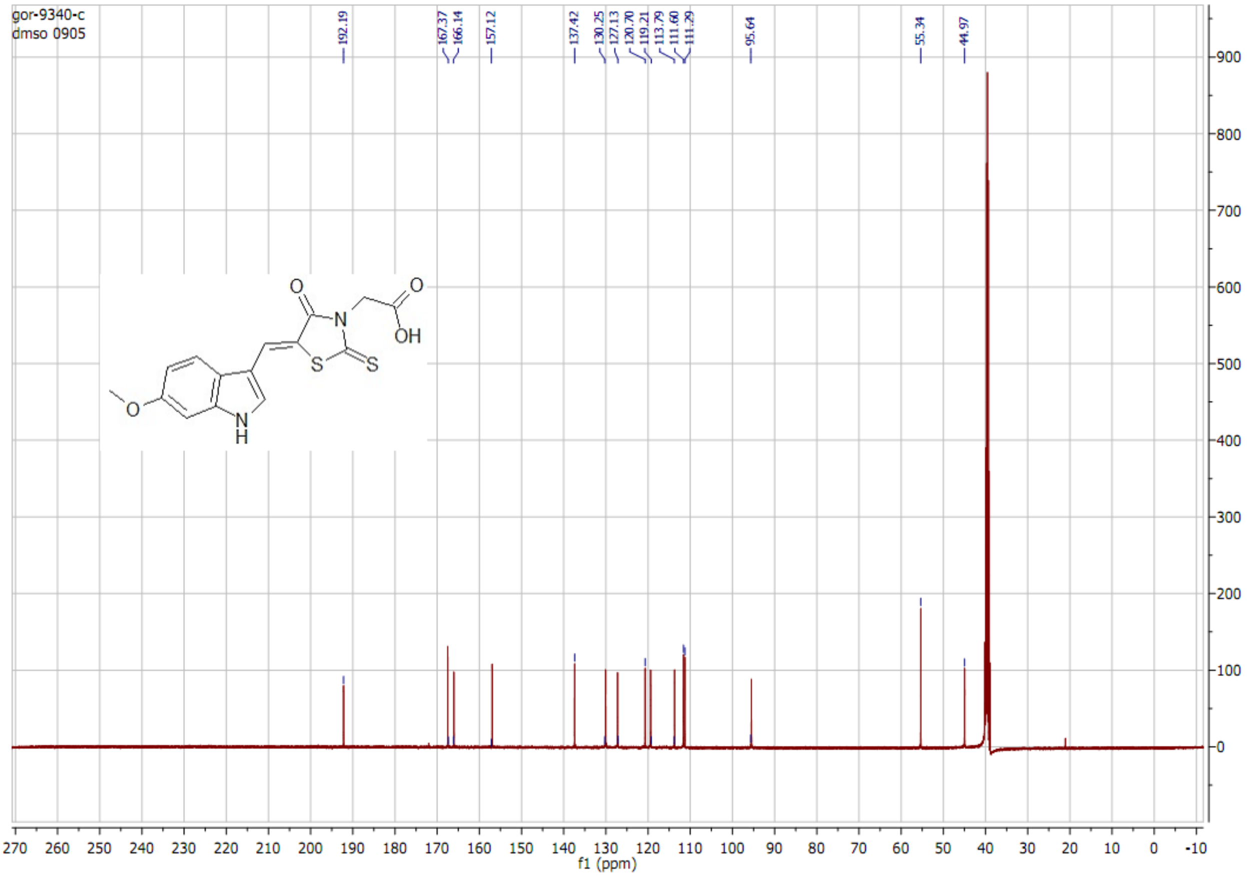

Figure S19. ^{13}C NMR spectrum (101 MHz, DMSO-d_6) of compound **4c**

-.o.-Syntez Purity Report -.o.-

System: Agilent 1200 LC/MSD SL
Separation column: Rapid Resolutionn HT Cartige 4.6x30mm, 1.8-Micron, Zorbx SB-C18

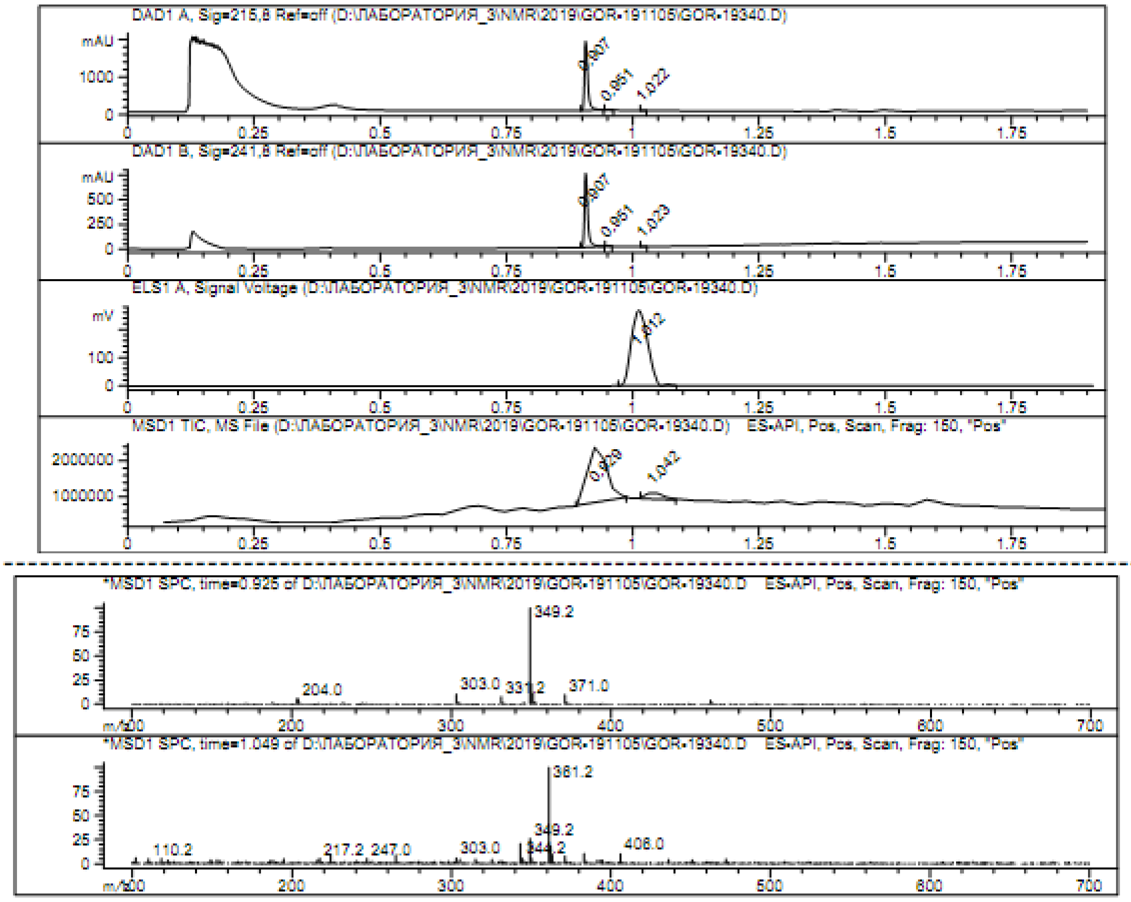

#	Signal	R.Time	Area %
1	DAD1 A, Sig=215,8 Ref=off	0.907	98.924
2		0.951	0.692
3		1.022	0.384

#	Signal	R.Time	Area %
1	DAD1 B, Sig=241,8 Ref=off	0.907	99.157
2		0.951	0.412
3		1.023	0.431

#	Signal	R.Time	Area %
1	ELS1 A, Signal Voltage	1.012	100.000

#	Signal	R.Time	Area %
1	MSD1 TIC, MS File	0.929	91.946
2		1.042	8.054

Inj. Date 06.11.2019

Figure S20. MS (ESI) spectrum of compound **4c.**

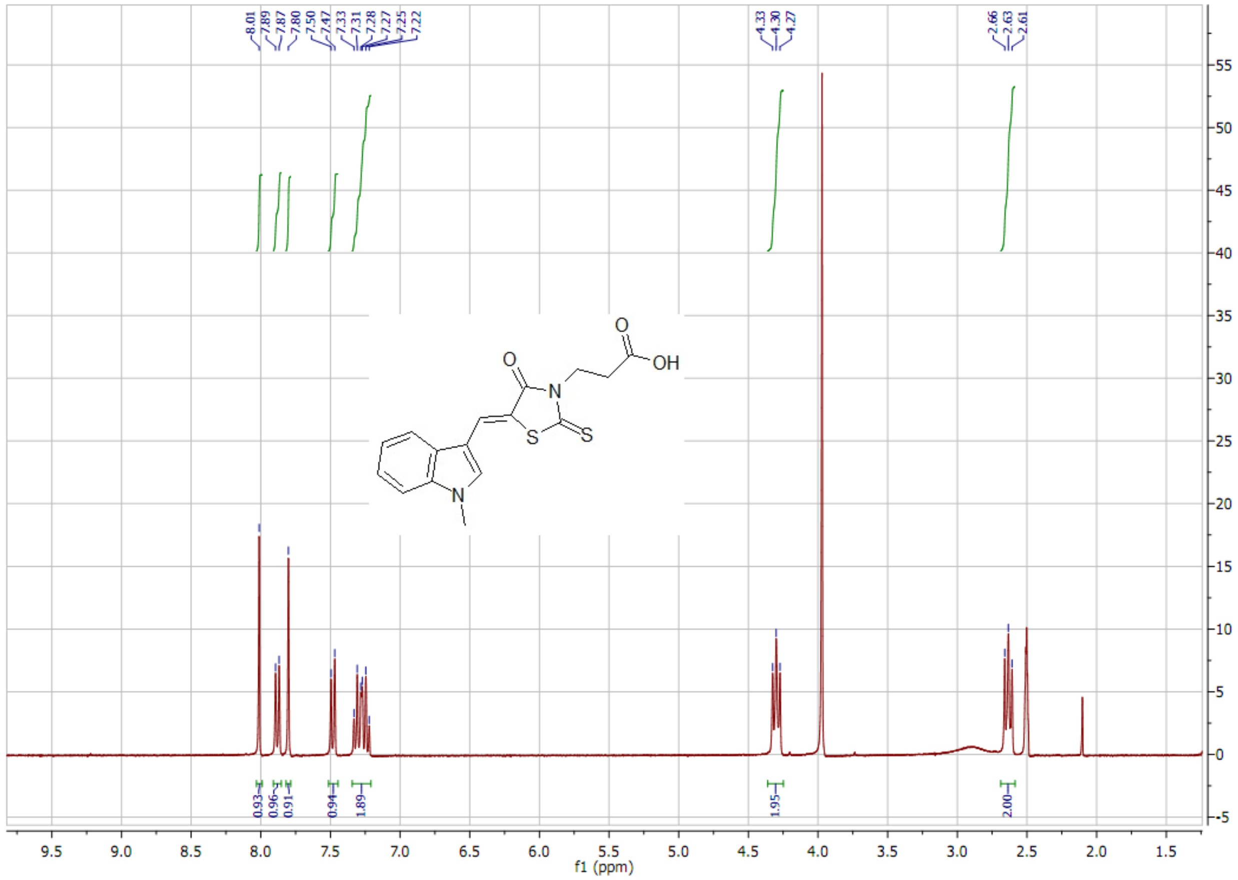

Figure S21. ^{1}HNMR spectrum (300 MHz, DMSO-d_6) of compound **4d**.

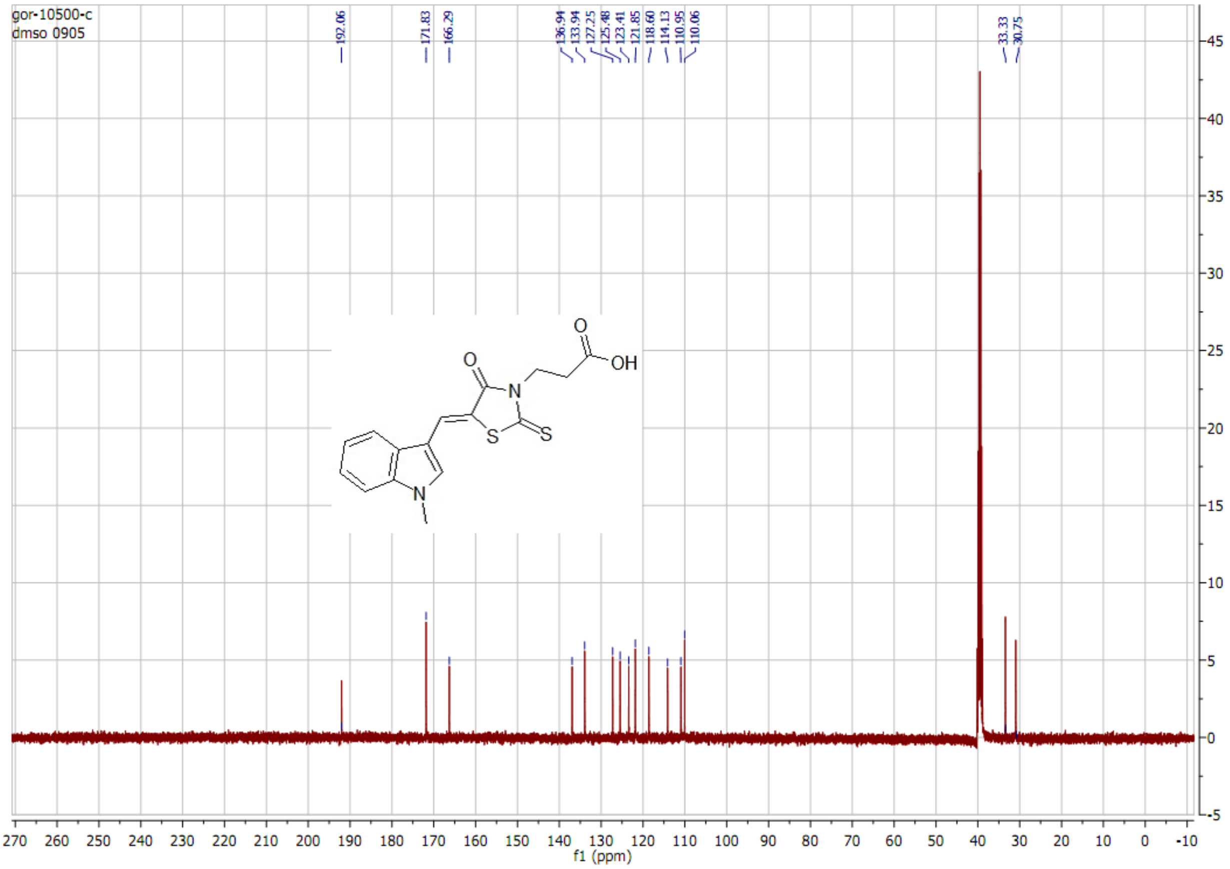

Figure S22. ^{13}C NMR spectrum (101 MHz, DMSO-d_6) of compound **4d**

Sample Name: GOR-10500

-.o.-Syntez Purity Report -.o.-

System: Agilent 1200 LC/MSD SL
Separation column: Rapid Resolutionn HT Cartige 4.6x30mm, 1.8-Micron, Zorbx SB-C18

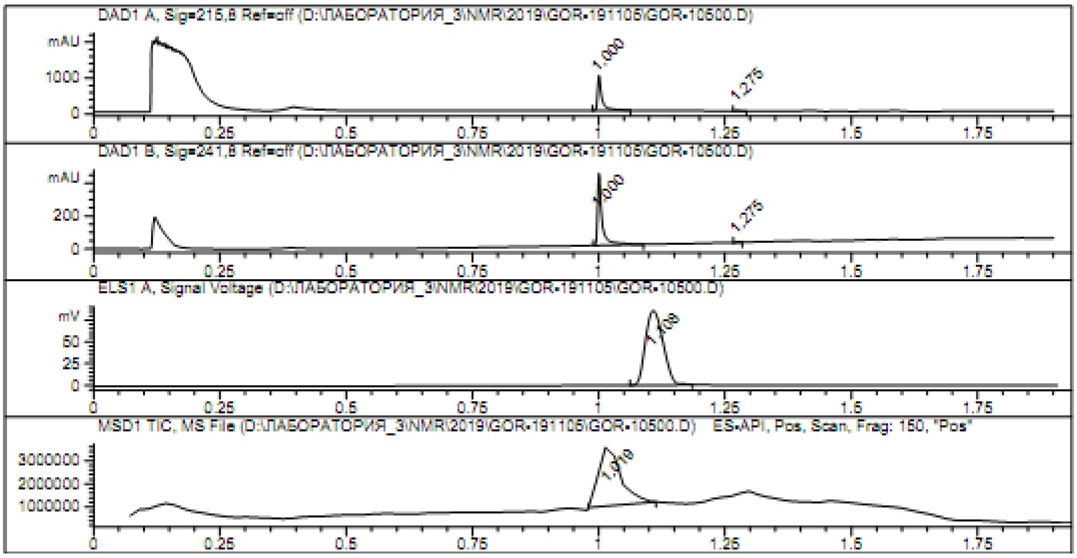

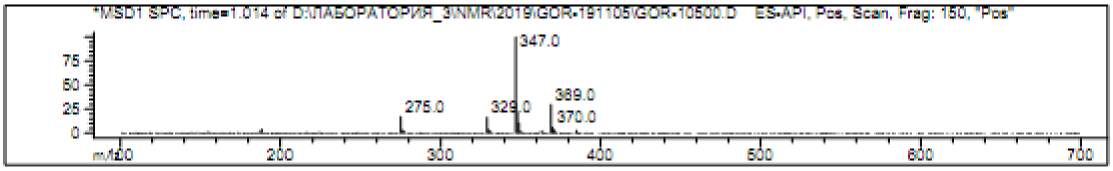

#	Signal	R.Time	Area %
1	DAD1 A, Sig=215,8 Ref=off	1.000	98.591
2		1.275	1.409

#	Signal	R.Time	Area %
1	DAD1 B, Sig=241,8 Ref=off	1.000	98.971
2		1.275	1.029

#	Signal	R.Time	Area %
1	ELS1 A, Signal Voltage	1.108	100.000

#	Signal	R.Time	Area %
1	MSD1 TIC, MS File	1.019	100.000

Inj. Data 05.11.2019

Figure S23. MS (ESI) spectrum of compound **4d.**

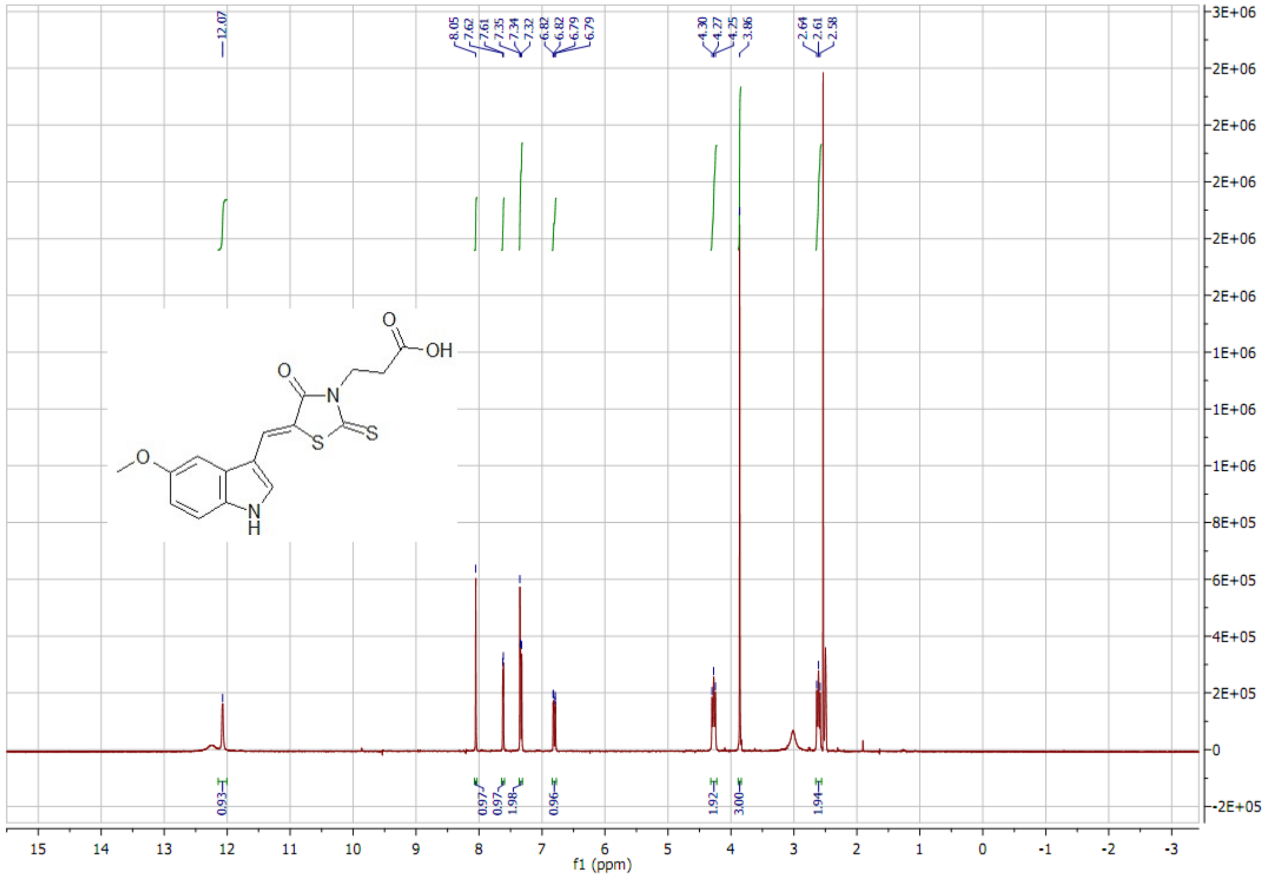

Figure S24. ^{1}HNMR spectrum (300 MHz, DMSO-d_6) of compound **4e**.

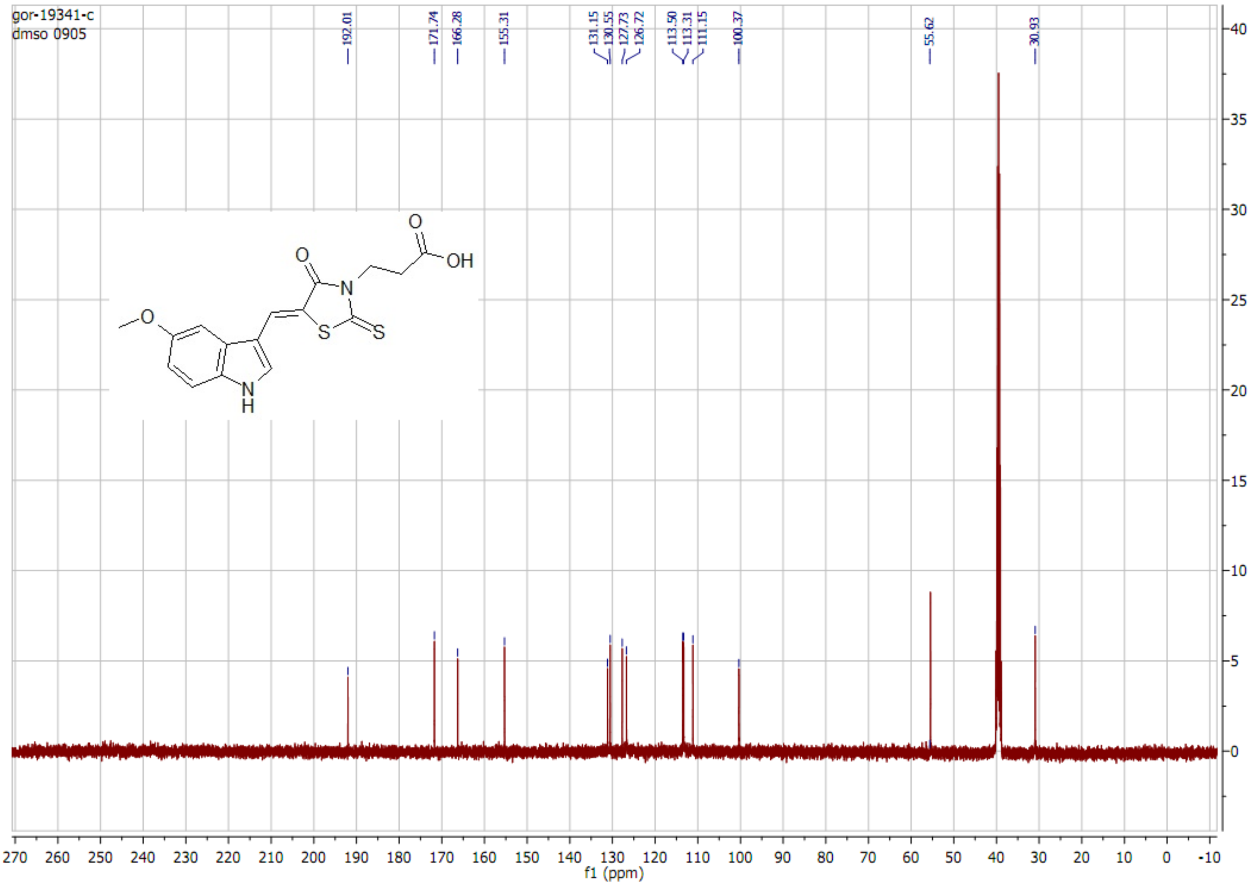

Figure S25. ^{13}C NMR spectrum (101 MHz, DMSO-d_6) of compound **4d**

Sample Name: GOR-19341

-.o.-Syntez Purity Report -.o.-

System: Agilent 1200 LC/MSD SL
Separation column: Rapid Resolutionn HT Cartige 4.6x30mm, 1.8-Micron, Zorbx SB-C18

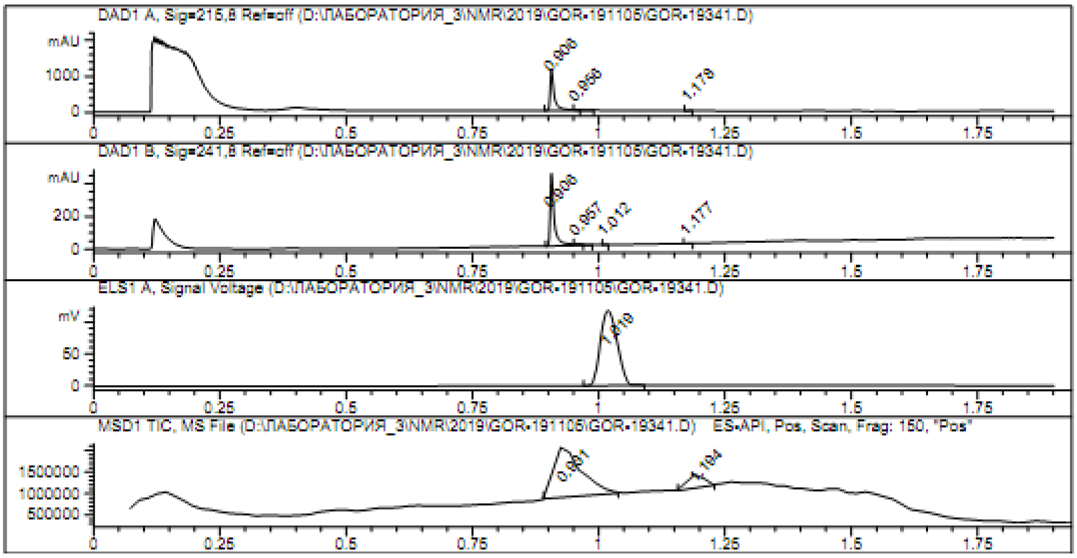

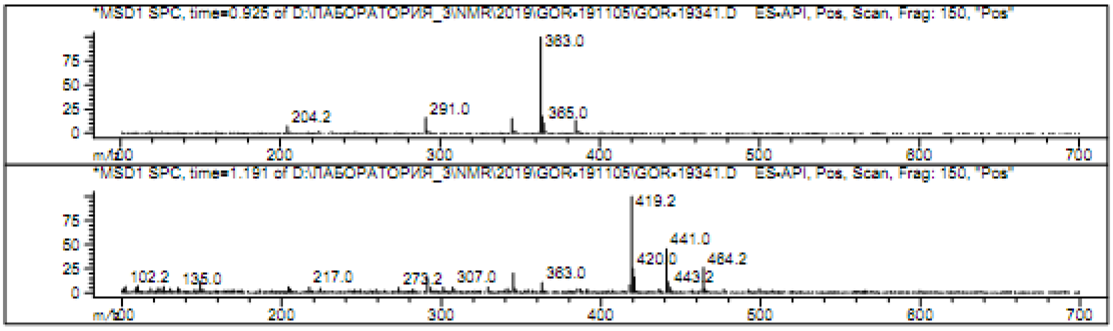

#	Signal	R.Time	Area %
1	DAD1 A, Sig=215,8 Ref=off	0.906	98.540
2		0.956	0.434
3		1.178	1.026

#	Signal	R.Time	Area %
1	DAD1 B, Sig=241,8 Ref=off	0.906	98.127
2		0.957	0.795
3		1.012	0.310
4		1.177	0.768

#	Signal	R.Time	Area %
1	ELS1 A, Signal Voltage	1.019	100.000

#	Signal	R.Time	Area %
1	MSD1 TIC, MS File	0.931	86.775
2		1.194	13.225

Inj. Data 05.11.2019

Figure S26. MS (ESI) spectrum of compound **4e.**

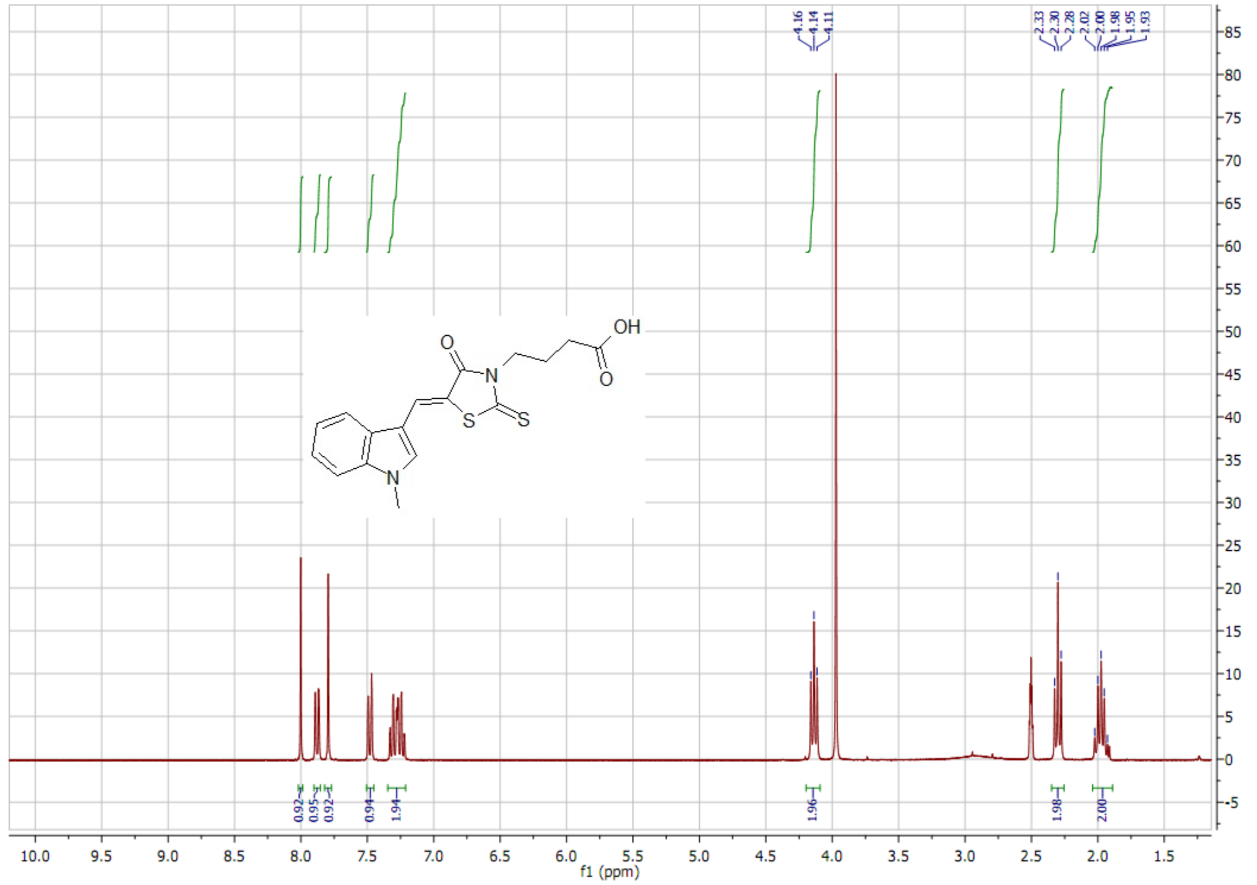

Figure S27. ^{1}HNMR spectrum (300 MHz, DMSO-d$_6$) of compound **4f**.

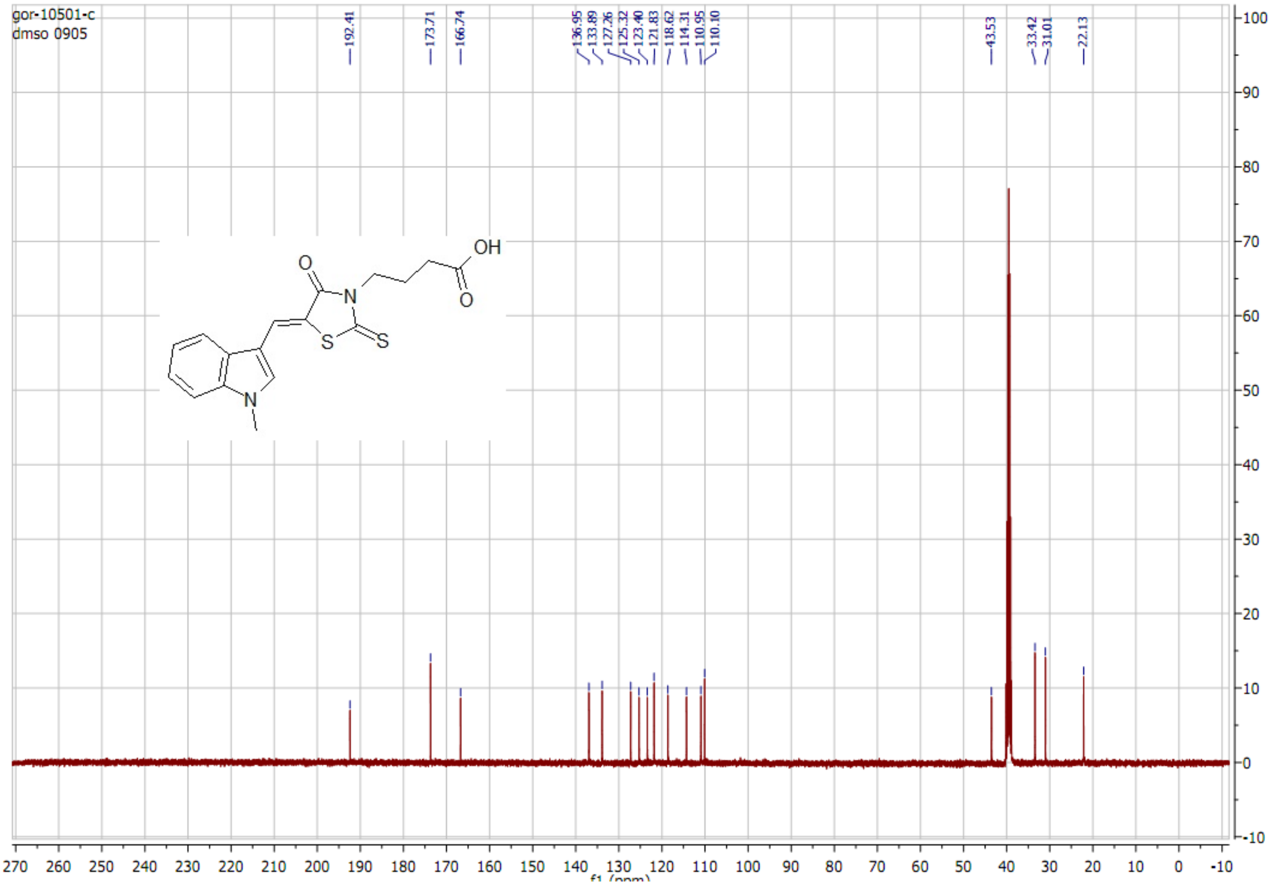

Figure S28. ^{13}C NMR spectrum (101 MHz, DMSO-d$_6$) of compound **4f**

Sample Name: GOR-10501

-.o.-Syntez Purity Report -.o.-

System: Agilent 1200 LC/MSD SL
Separation column: Rapid Resolutionn HT Cartige 4.6x30mm, 1.8-Micron, Zorbx SB-C18

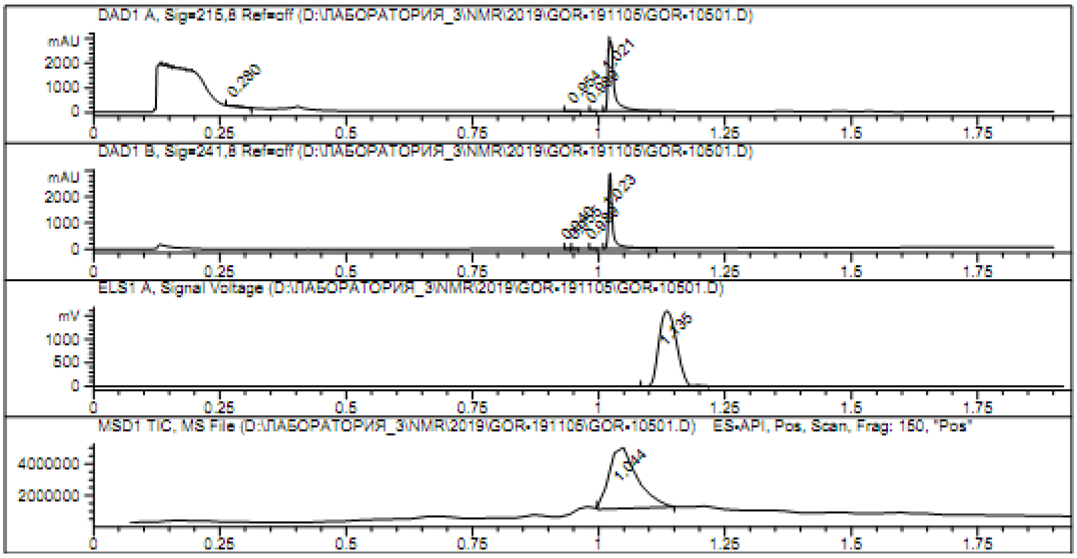

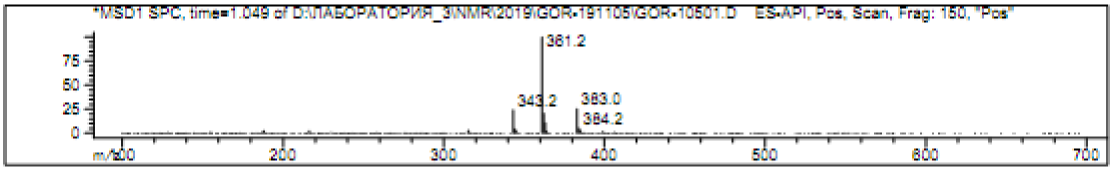

#	Signal	R.Time	Area %
1	DAD1 A, Sig=215,8 Ref=off	0.280	1.868
2		0.954	0.299
3		0.989	0.128
4		1.021	97.706

#	Signal	R.Time	Area %
1	DAD1 B, Sig=241,8 Ref=off	0.940	0.068
2		0.955	0.092
3		0.989	0.139
4		1.023	99.701

#	Signal	R.Time	Area %
1	ELS1 A, Signal Voltage	1.135	100.000

#	Signal	R.Time	Area %
1	MSD1 TIC, MS File	1.044	100.000

Inj. Data 06.11.2019

Figure S29. MS (ESI) spectrum of compound **4f.**

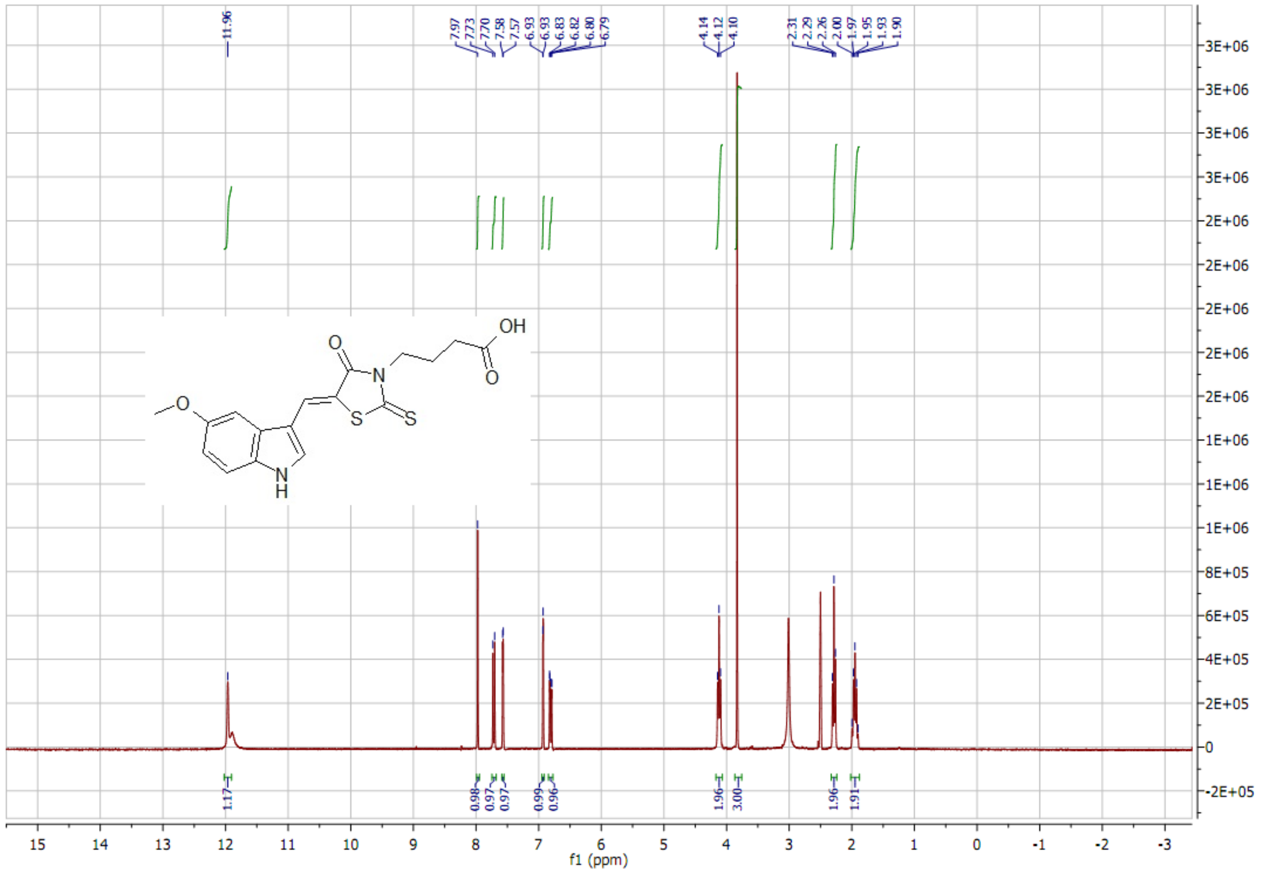

Figure S30. ^{1}HNMR spectrum (300 MHz, DMSO-d_6) of compound **4g**.

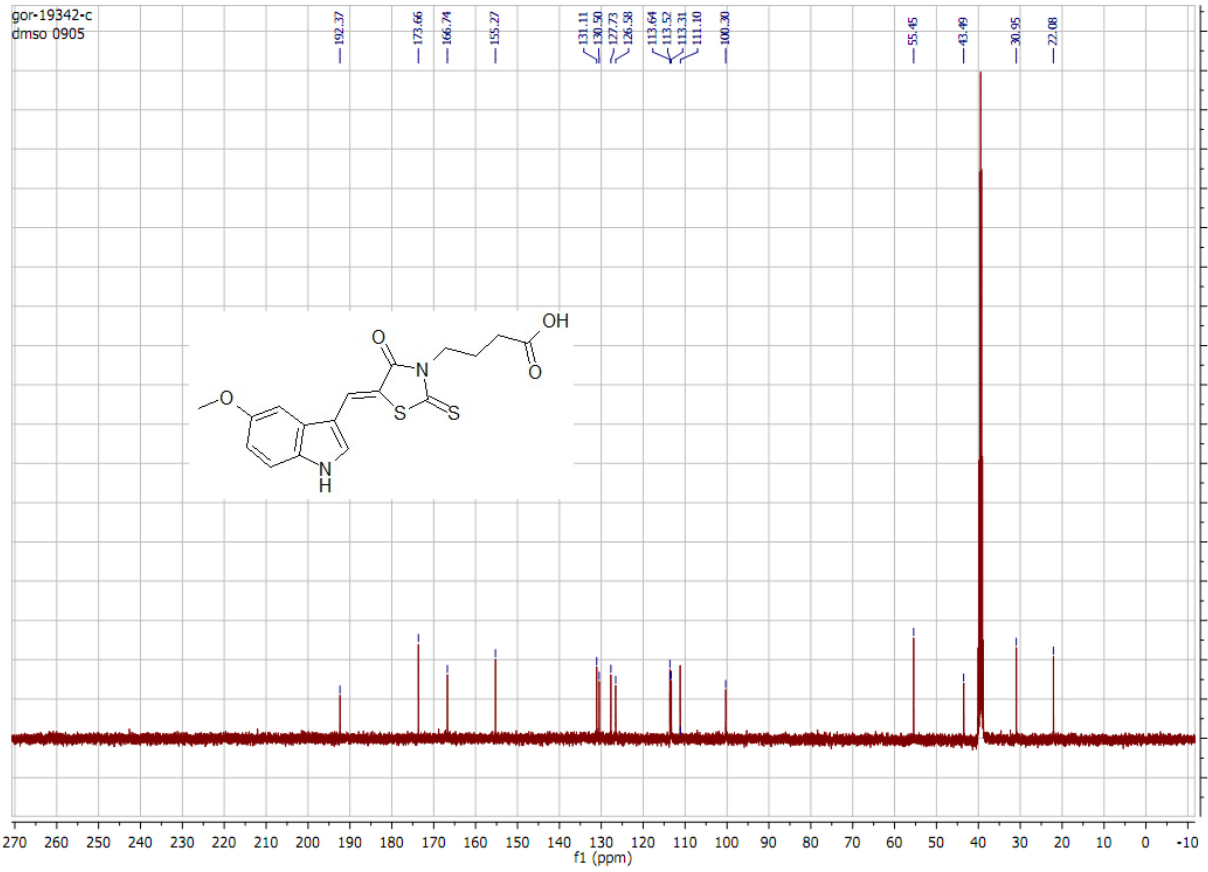

Figure S31. ^{13}C NMR spectrum (101 MHz, DMSO-d_6) of compound **4g**

Sample Name: GOR-19342

-.o.-Syntez Purity Report -.o.-

System: Agilent 1200 LC/MSD SL
Separation column: Rapid Resolutionn HT Cartige 4.6x30mm, 1.8-Micron, Zorbx SB-C18

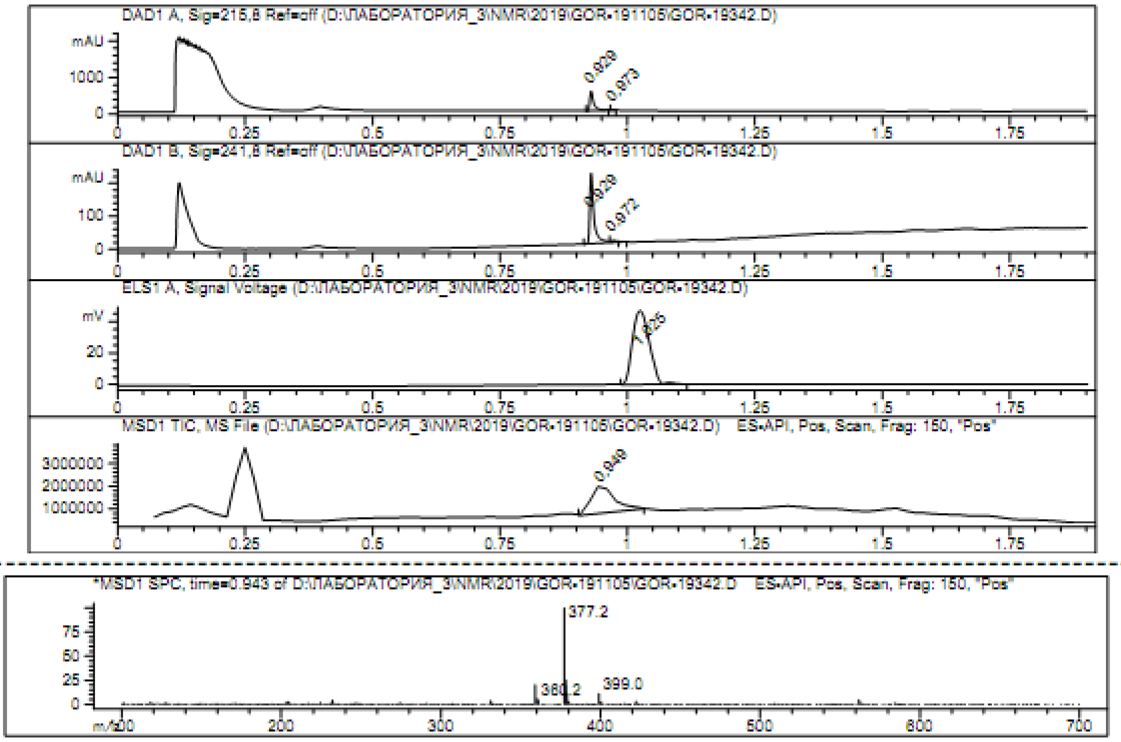

#	Signal	R.Time	Area %
1	DAD1 A, Sig=215,8 Ref=off	0.929	99.533
2		0.973	0.467

#	Signal	R.Time	Area %
1	DAD1 B, Sig=241,8 Ref=off	0.929	98.942
2		0.972	1.058

#	Signal	R.Time	Area %
1	ELS1 A, Signal Voltage	1.025	100.000

#	Signal	R.Time	Area %
1	MSD1 TIC, MS File	0.949	100.000

Inj. Data 05.11.2019

Figure S32. MS (ESI) spectrum of compound **4g.**

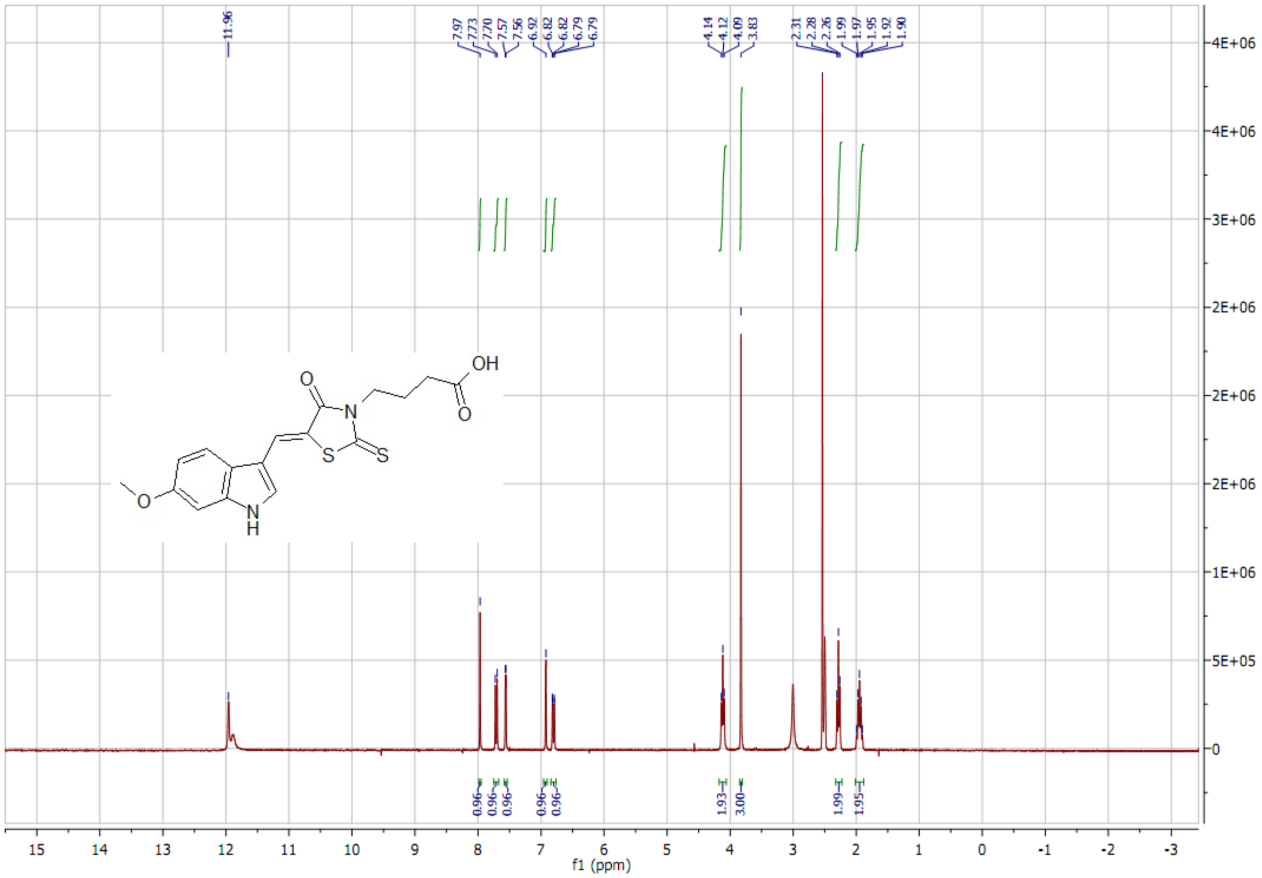

Figure S33. ^{1}HNMR spectrum (300 MHz, DMSO-d_6) of compound **4h**.

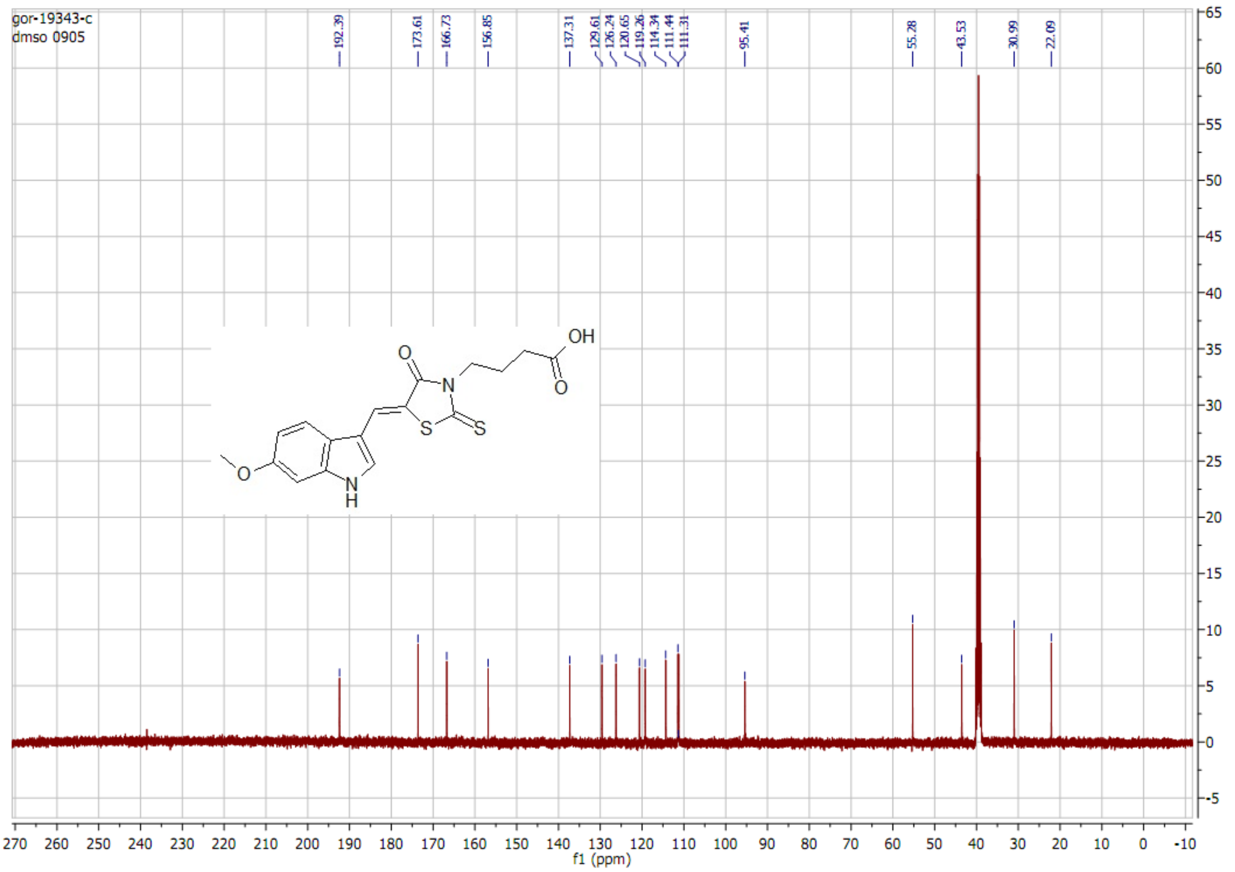

Figure S34. ^{13}C NMR spectrum (101 MHz, DMSO-d_6) of compound **4h**

Sample Name: GOR-19343

-.o.-Syntez Purity Report -.o.-

System: Agilent 1200 LC/MSD SL
Separation column: Rapid Resolutionn HT Cartige 4.6x30mm, 1.8-Micron, Zorbx SB-C18

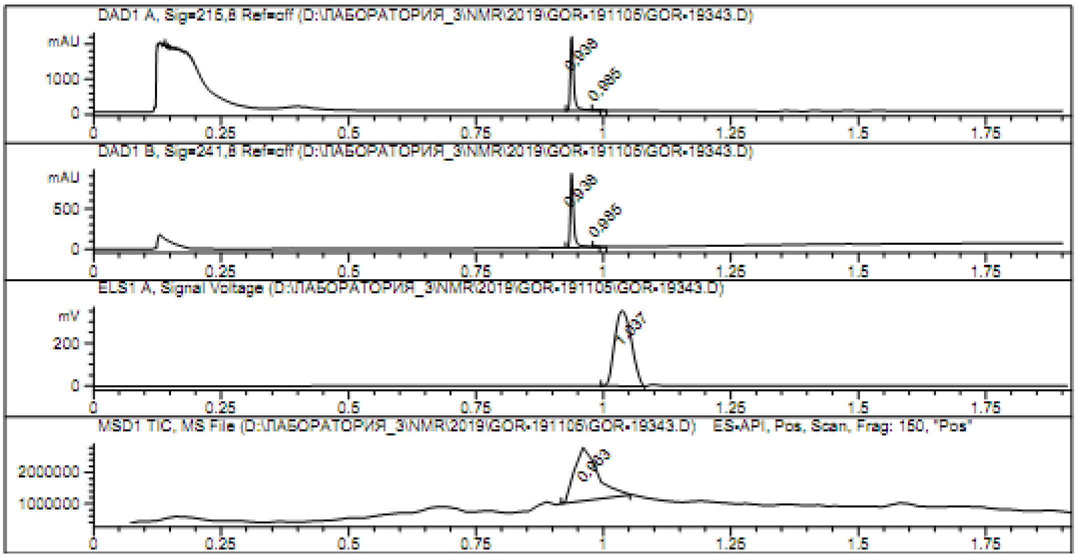

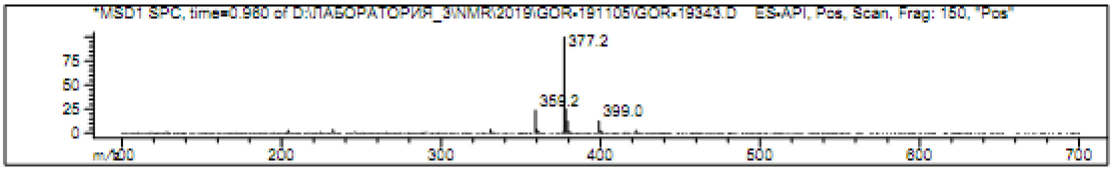

#	Signal	R.Time	Area %
1	DAD1 A, Sig=215,8 Ref=off	0.938	99.403
2		0.985	0.597

#	Signal	R.Time	Area %
1	DAD1 B, Sig=241,8 Ref=off	0.938	99.433
2		0.985	0.567

#	Signal	R.Time	Area %
1	ELS1 A, Signal Voltage	1.037	100.000

#	Signal	R.Time	Area %
1	MSD1 TIC, MS File	0.963	100.000

Inj. Date 06.11.2019

Figure S35. MS (ESI) spectrum of compound **4h.**

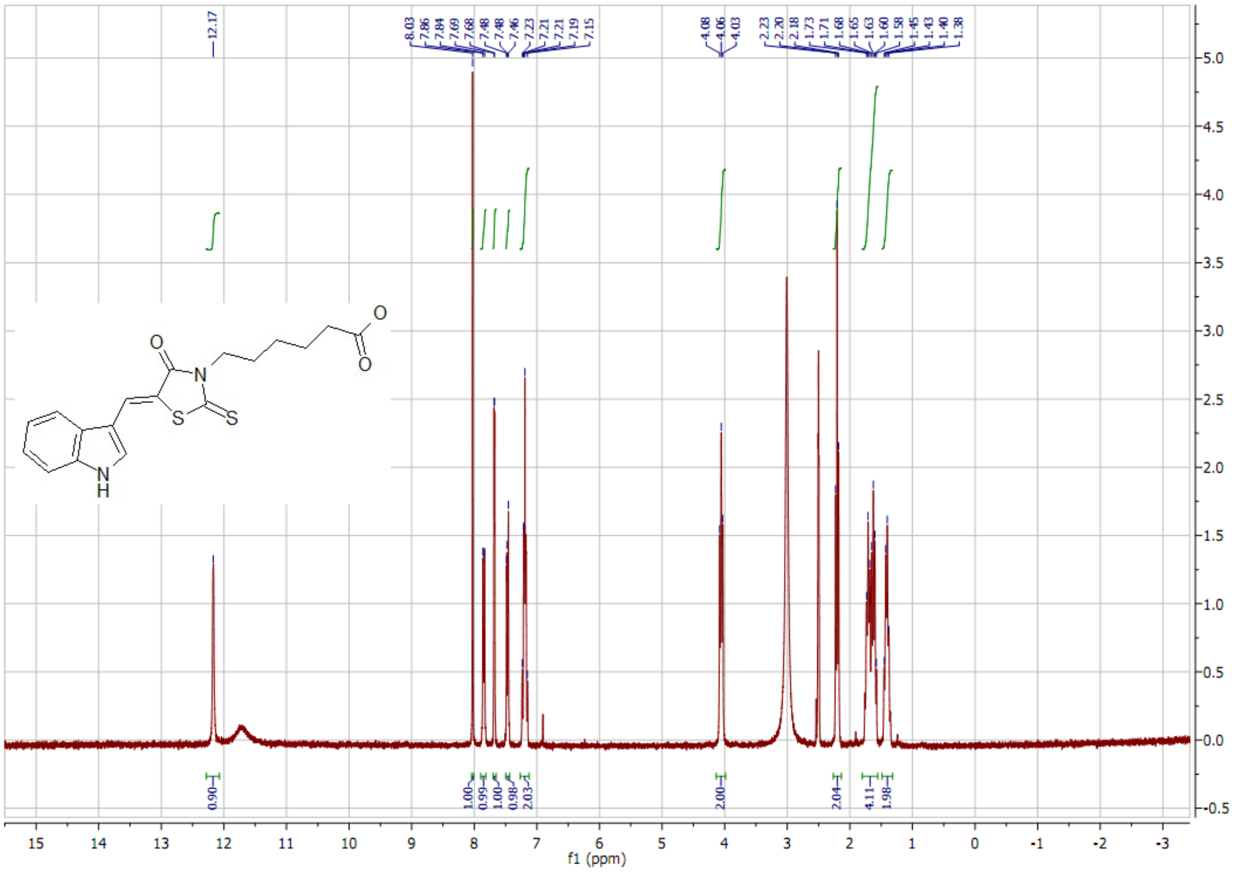

Figure S36. ^{1}HNMR spectrum (300 MHz, DMSO-d_6) of compound **4i**.

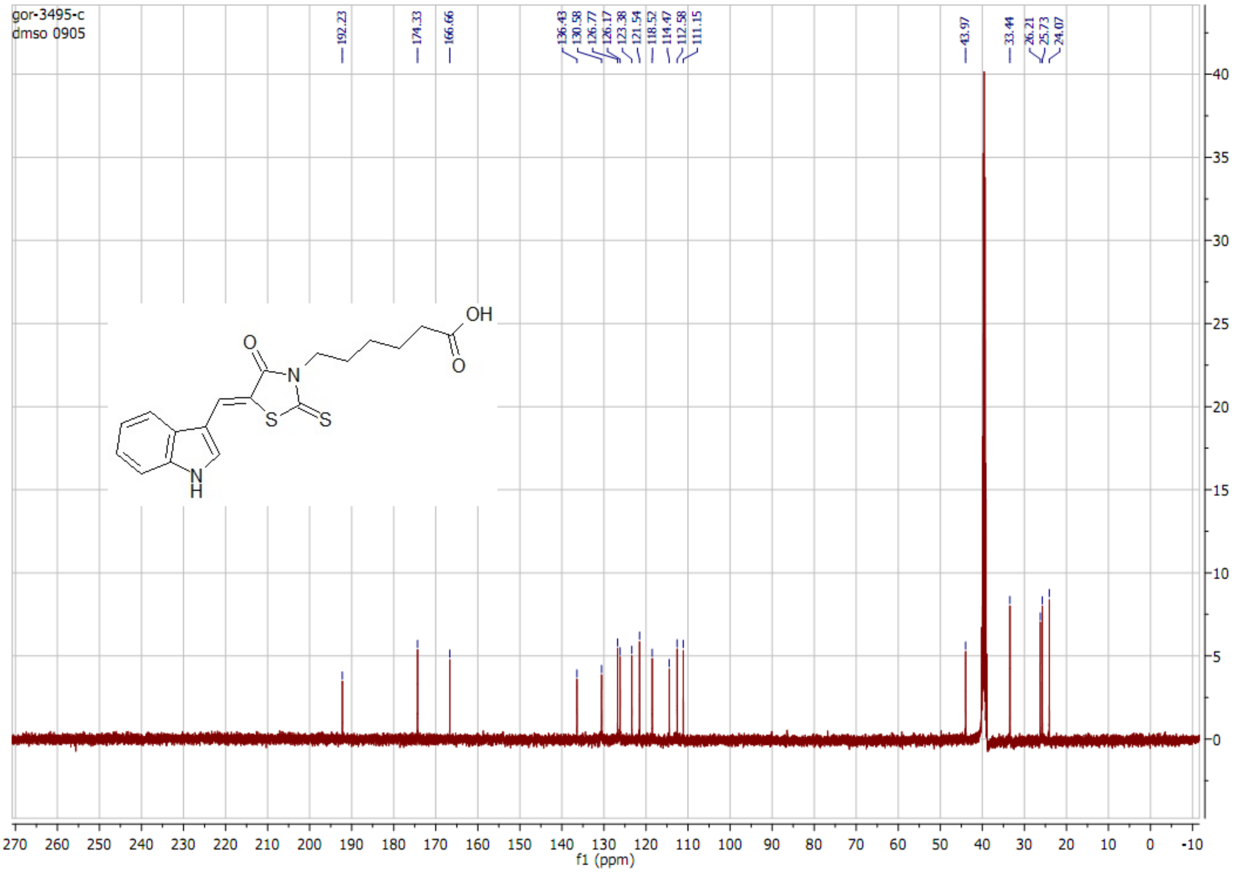

Figure S37. ^{13}C NMR spectrum (101 MHz, DMSO-d_6) of compound **4i**

Sample Name: GOR-3495

-.o.-Syntez Purity Report -.o.-

System: Agilent 1200 LC/MSD SL
Separation column: Rapid Resolutionn HT Cartige 4.6x30mm, 1.8-Micron, Zorbx SB-C18

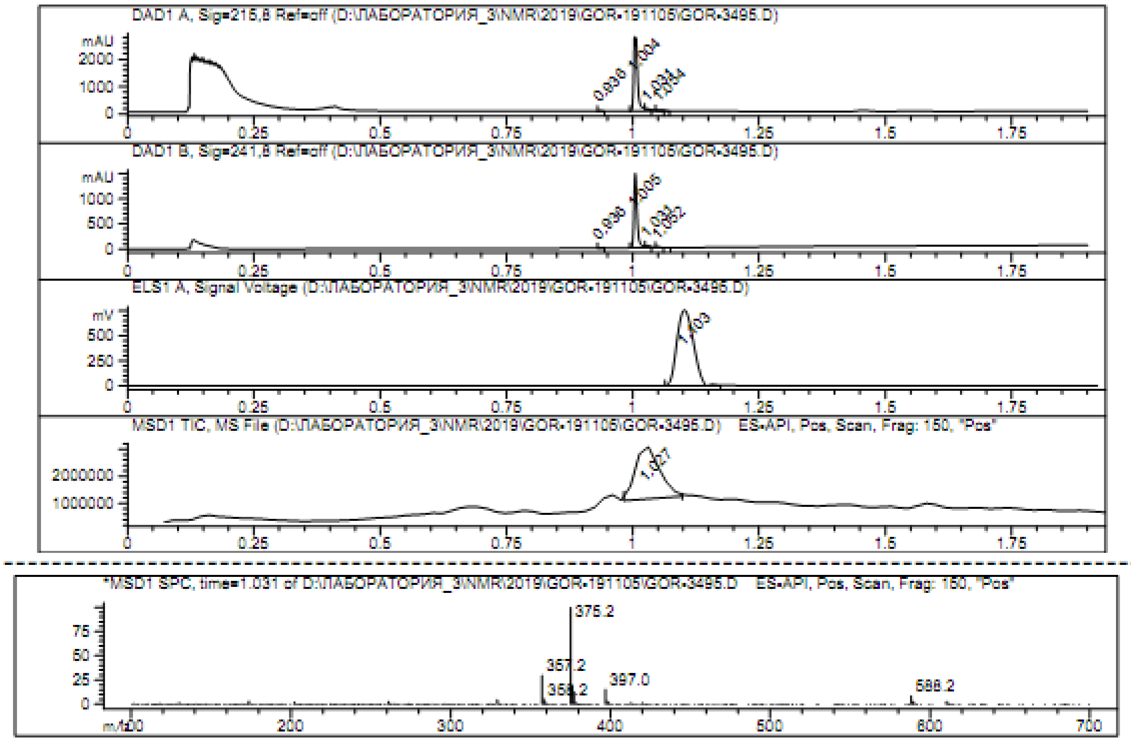

#	Signal	R.Time	Area %
1	DAD1 A, Sig=215,8 Ref=off	0.936	0.521
2		1.004	98.688
3		1.031	0.464
4		1.054	0.327

#	Signal	R.Time	Area %
1	DAD1 B, Sig=241,8 Ref=off	0.936	0.314
2		1.005	98.767
3		1.031	0.621
4		1.052	0.299

#	Signal	R.Time	Area %
1	ELS1 A, Signal Voltage	1.103	100.000

#	Signal	R.Time	Area %
1	MSD1 TIC, MS File	1.027	100.000

Inj. Date 06.11.2019

Figure S38. MS (ESI) spectrum of compound **4i.**

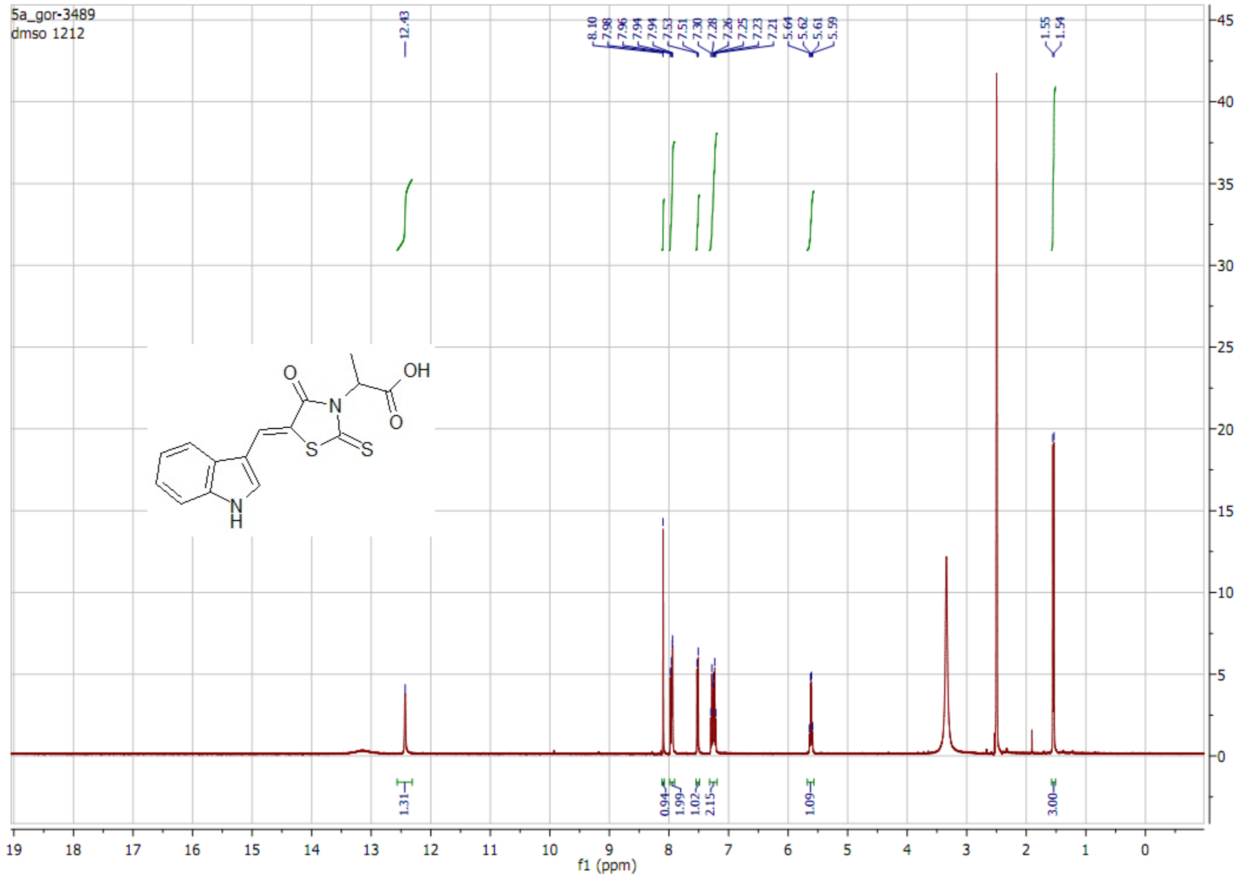

Figure S39. ^{1}HNMR spectrum (400 MHz, DMSO-d_6) of compound **5a**.

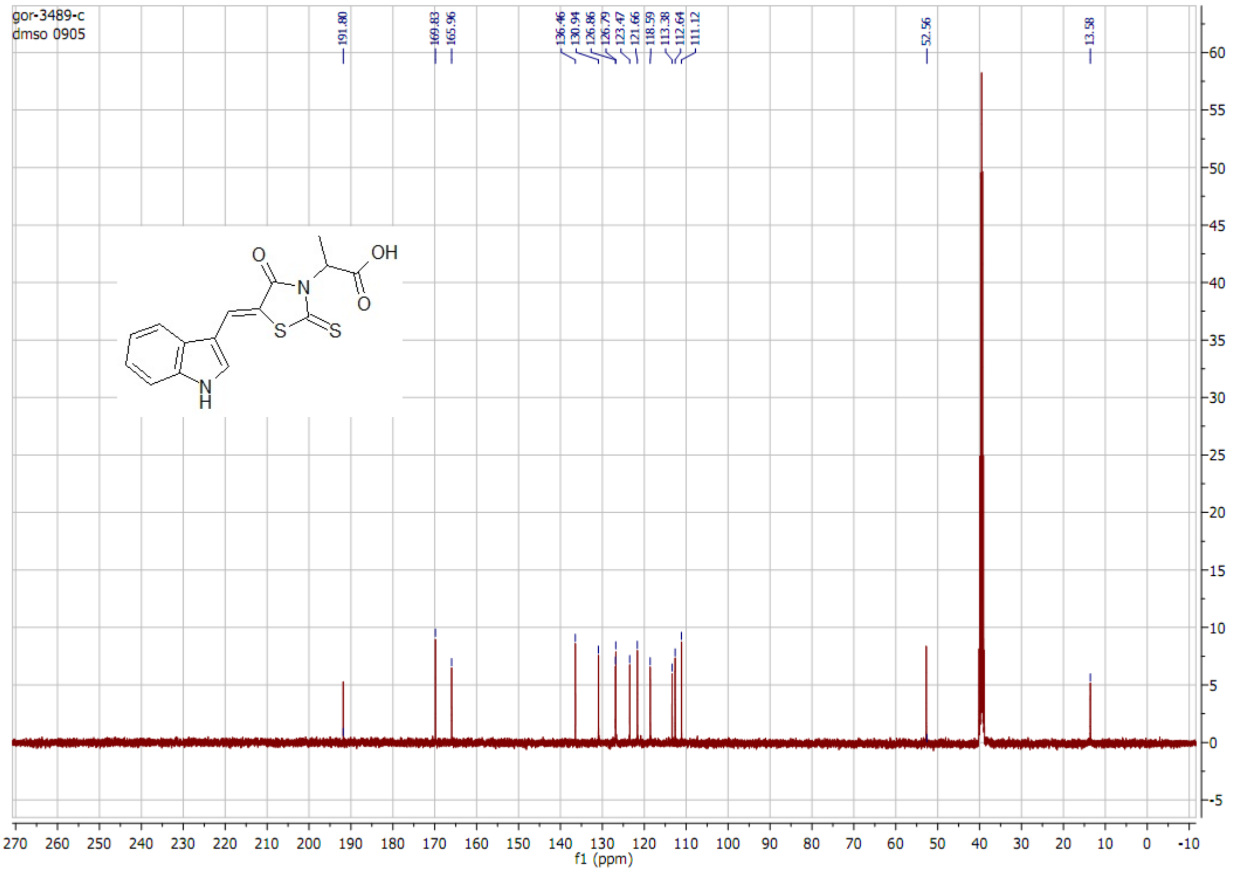

Figure S40. ^{13}C NMR spectrum (101 MHz, DMSO-d_6) of compound **5a.**

Sample Name: GOR-3489

-.o.-Syntez Purity Report -.o.-

System: Agilent 1200 LC/MSD SL
Separation column: Rapid Resolutionn HT Cartige 4.6x30mm, 1.8-Micron, Zorbx SB-C18

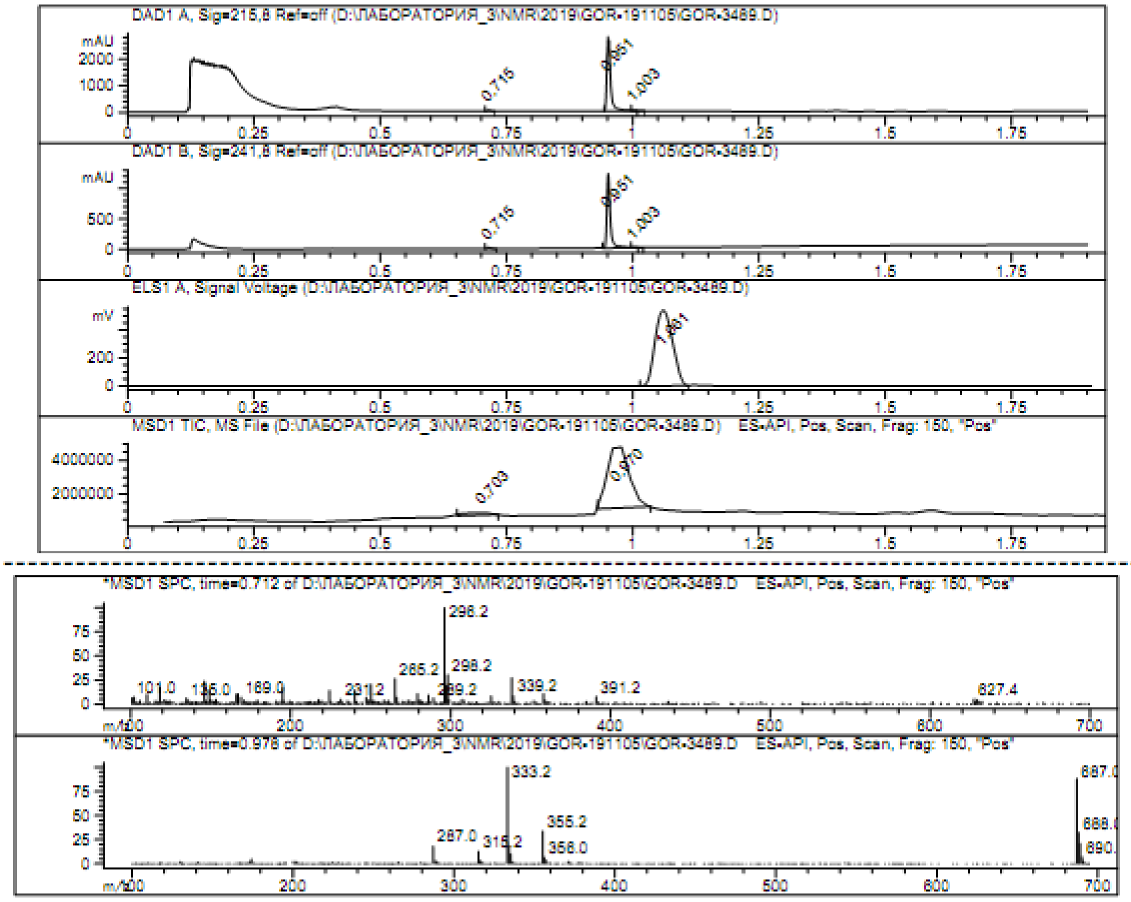

#	Signal	R.Time	Area %
1	DAD1 A, Sig=215,8 Ref=off	0.715	1.110
2		0.951	98.711
3		1.003	0.179

#	Signal	R.Time	Area %
1	DAD1 B, Sig=241,8 Ref=off	0.715	1.847
2		0.951	97.819
3		1.003	0.334

#	Signal	R.Time	Area %
1	ELS1 A, Signal Voltage	1.061	100.000

#	Signal	R.Time	Area %
1	MSD1 TIC, MS File	0.703	4.220
2		0.970	95.780

Inj. Date 06.11.2019

Figure S41. MS (ESI) spectrum of compound **5a.**

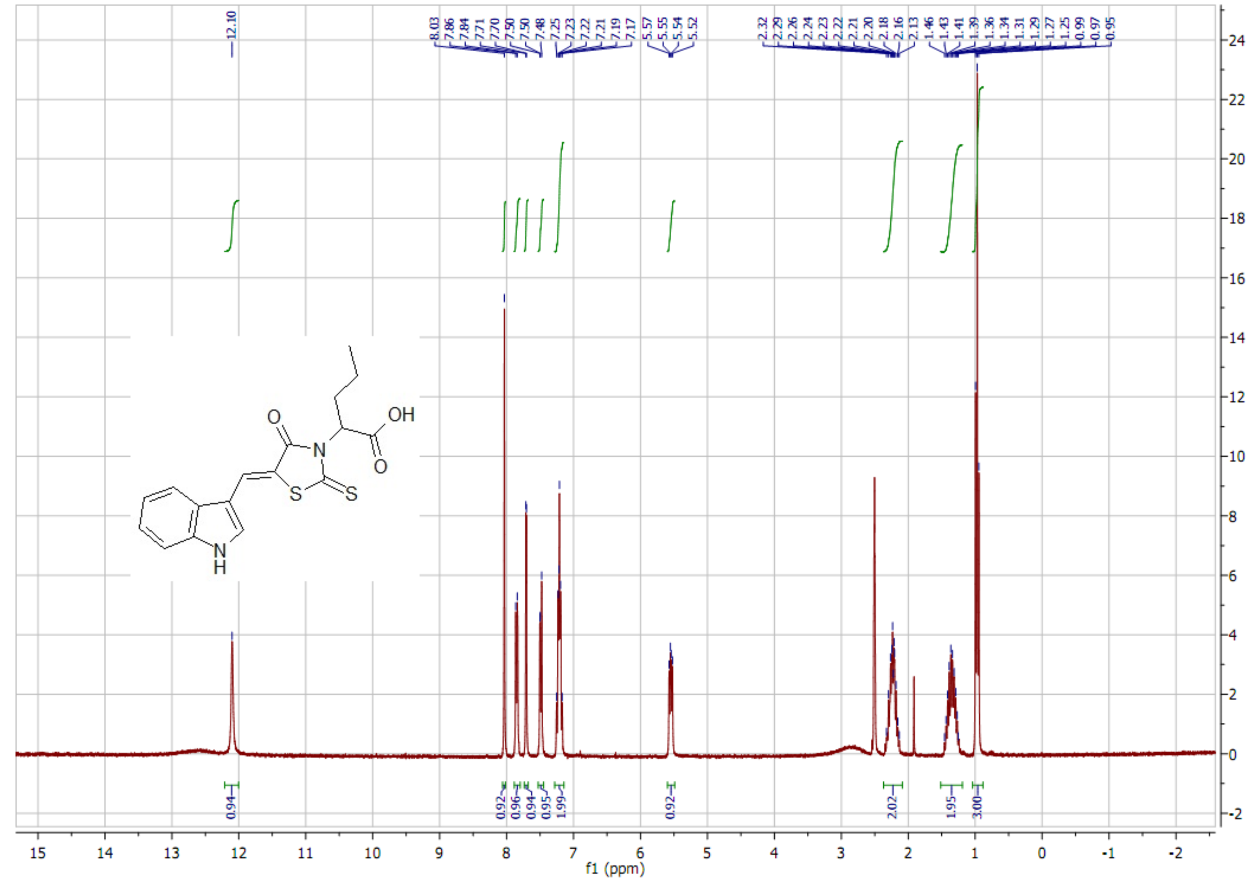

Figure S42. ^{1}HNMR spectrum (300 MHz, DMSO-d_6) of compound **5b**.

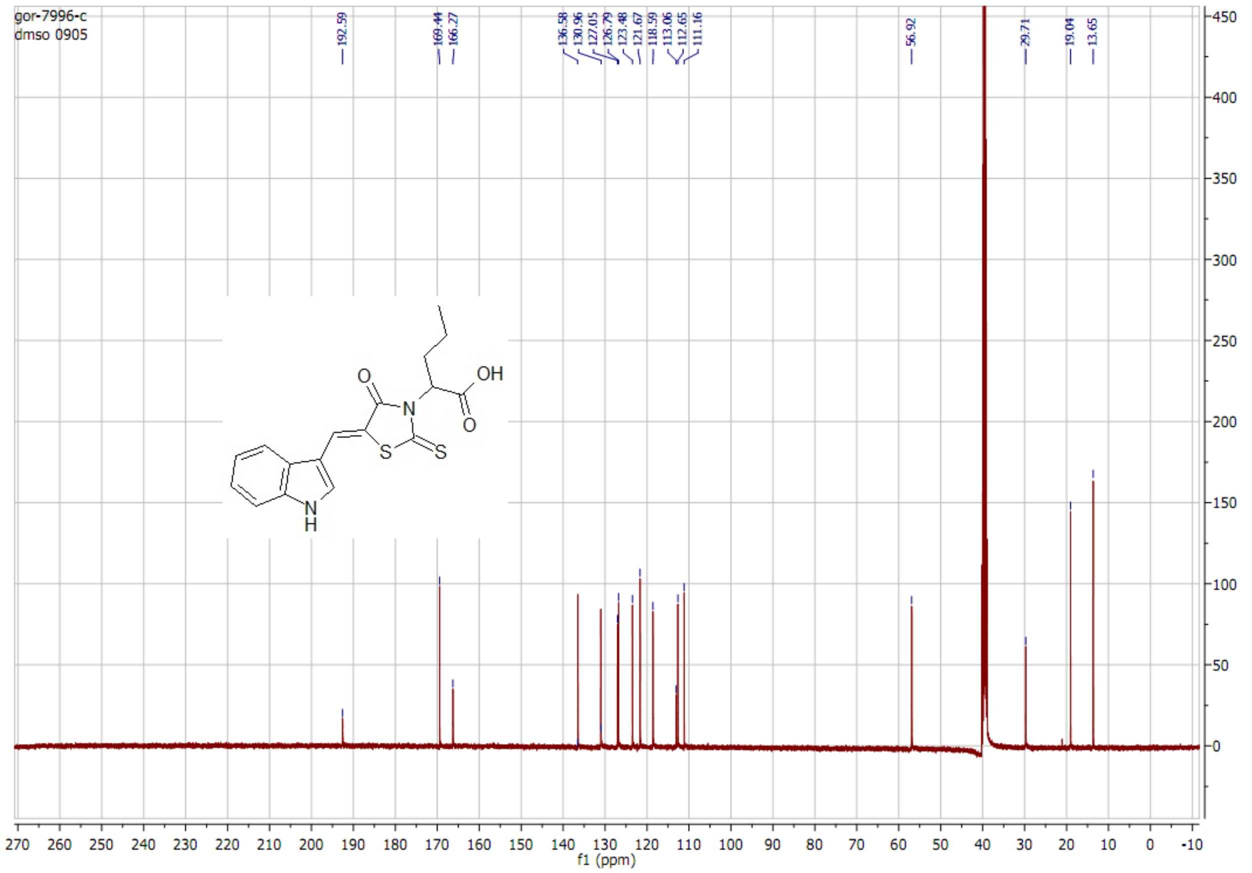

Figure S43. ^{13}C NMR spectrum (101 MHz, DMSO-d_6) of compound **5b.**

Sample Name: GOR-7996

-.o.-Syntez Purity Report -.o.-

System: Agilent 1200 LC/MSD SL
Separation column: Rapid Resolutionn HT Cartige 4.6x30mm, 1.8-Micron, Zorbx SB-C18

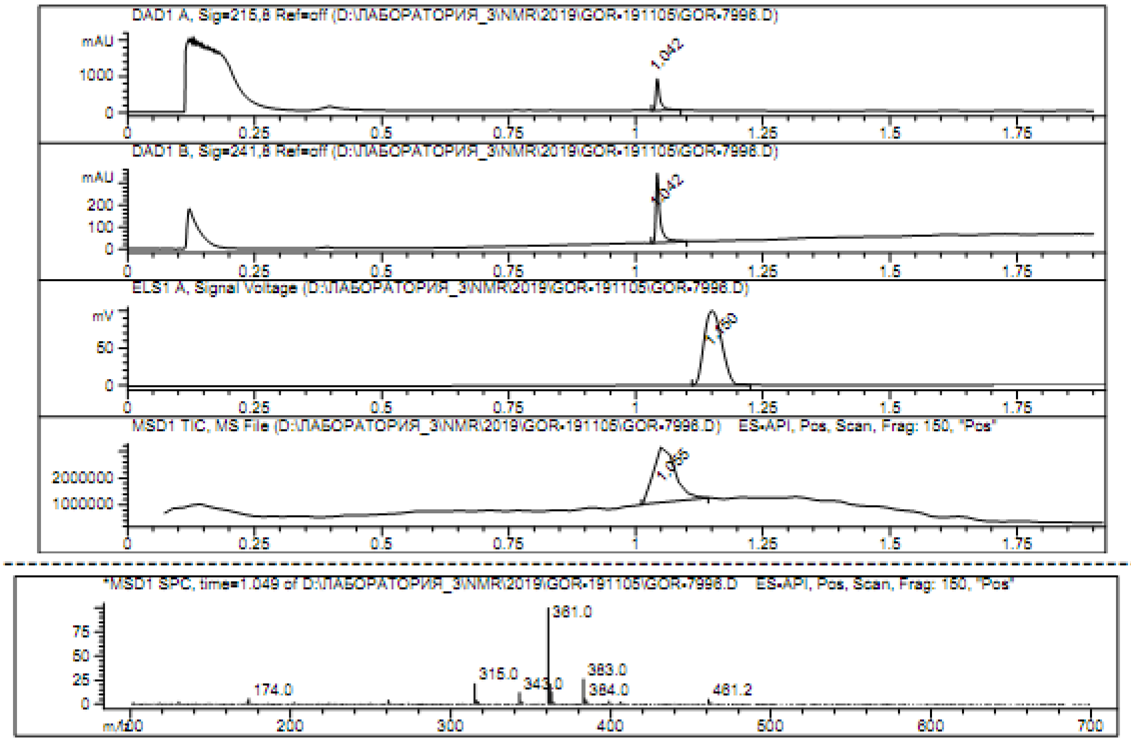

#	Signal	R.Time	Area %
1	DAD1 A, Sig=215,8 Ref=off	1.042	100.000

#	Signal	R.Time	Area %
1	DAD1 B, Sig=241,8 Ref=off	1.042	100.000

#	Signal	R.Time	Area %
1	ELS1 A, Signal Voltage	1.150	100.000

#	Signal	R.Time	Area %
1	MSD1 TIC, MS File	1.055	100.000

Inj. Date 05.11.2019

Figure S44. MS (ESI) spectrum of compound **5b.**

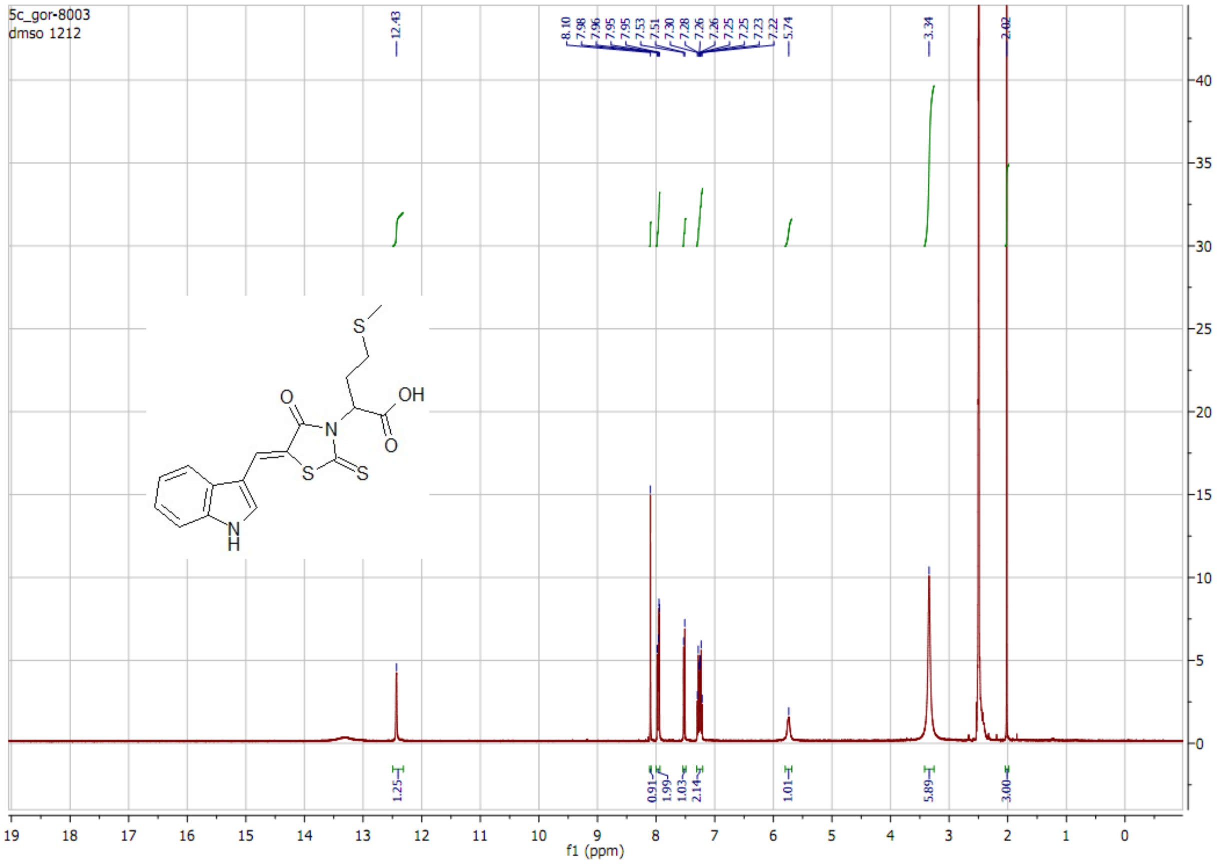

Figure S45. ^{1}HNMR spectrum (400 MHz, DMSO-d$_6$) of compound **5c**.

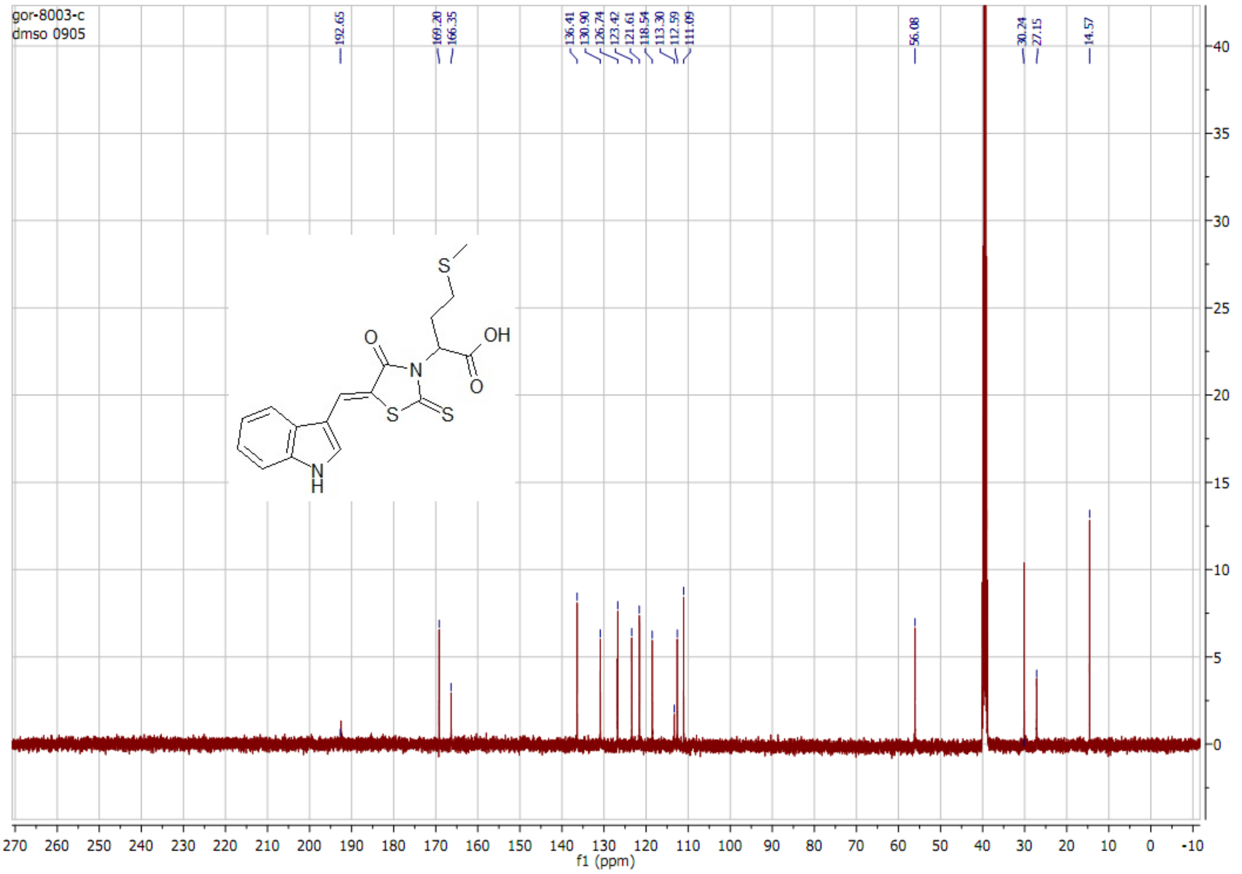

Figure S46. ^{13}C NMR spectrum (101 MHz, DMSO-d$_6$) of compound **5c.**

Sample Name: GOR-8003

-.o.-Syntez Purity Report -.o.-

System: Agilent 1200 LC/MSD SL
Separation column: Rapid Resolutionn HT Cartige 4.6x30mm, 1.8-Micron, Zorbx SB-C18

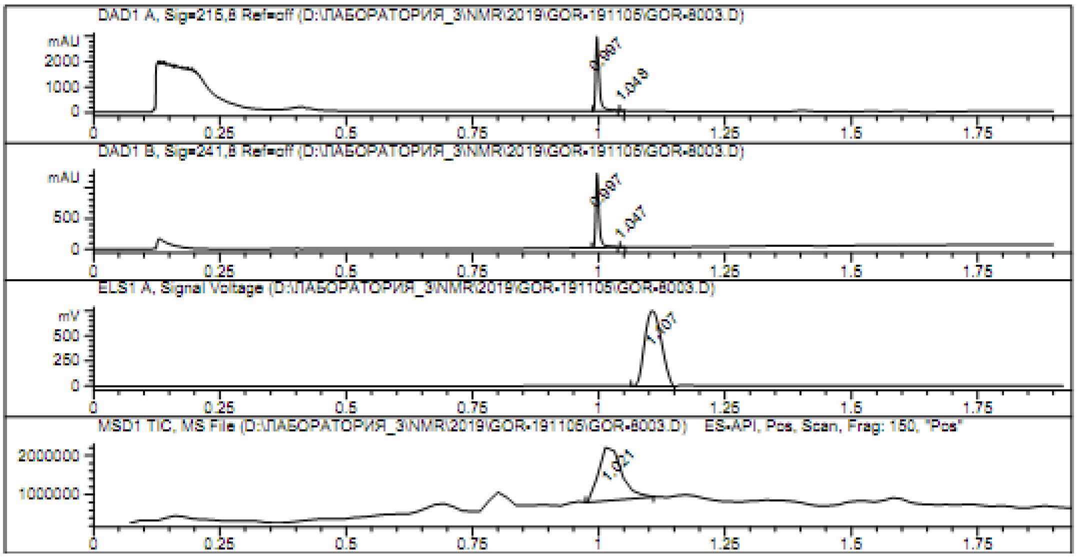

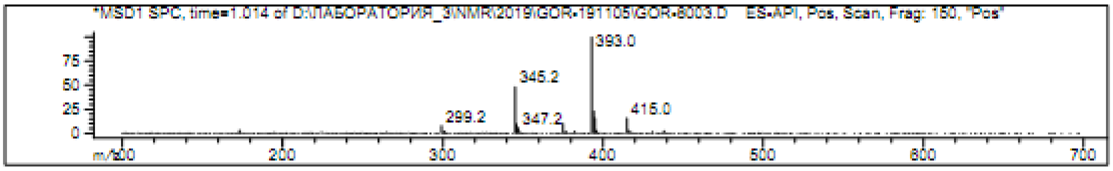

#	Signal	R.Time	Area %
1	DAD1 A, Sig=215,8 Ref=off	0.997	99.691
2		1.048	0.309

#	Signal	R.Time	Area %
1	DAD1 B, Sig=241,8 Ref=off	0.997	99.552
2		1.047	0.448

#	Signal	R.Time	Area %
1	ELS1 A, Signal Voltage	1.107	100.000

#	Signal	R.Time	Area %
1	MSD1 TIC, MS File	1.021	100.000

Inj. Data 06.11.2019

Figure S47. MS (ESI) spectrum of compound **5c.**

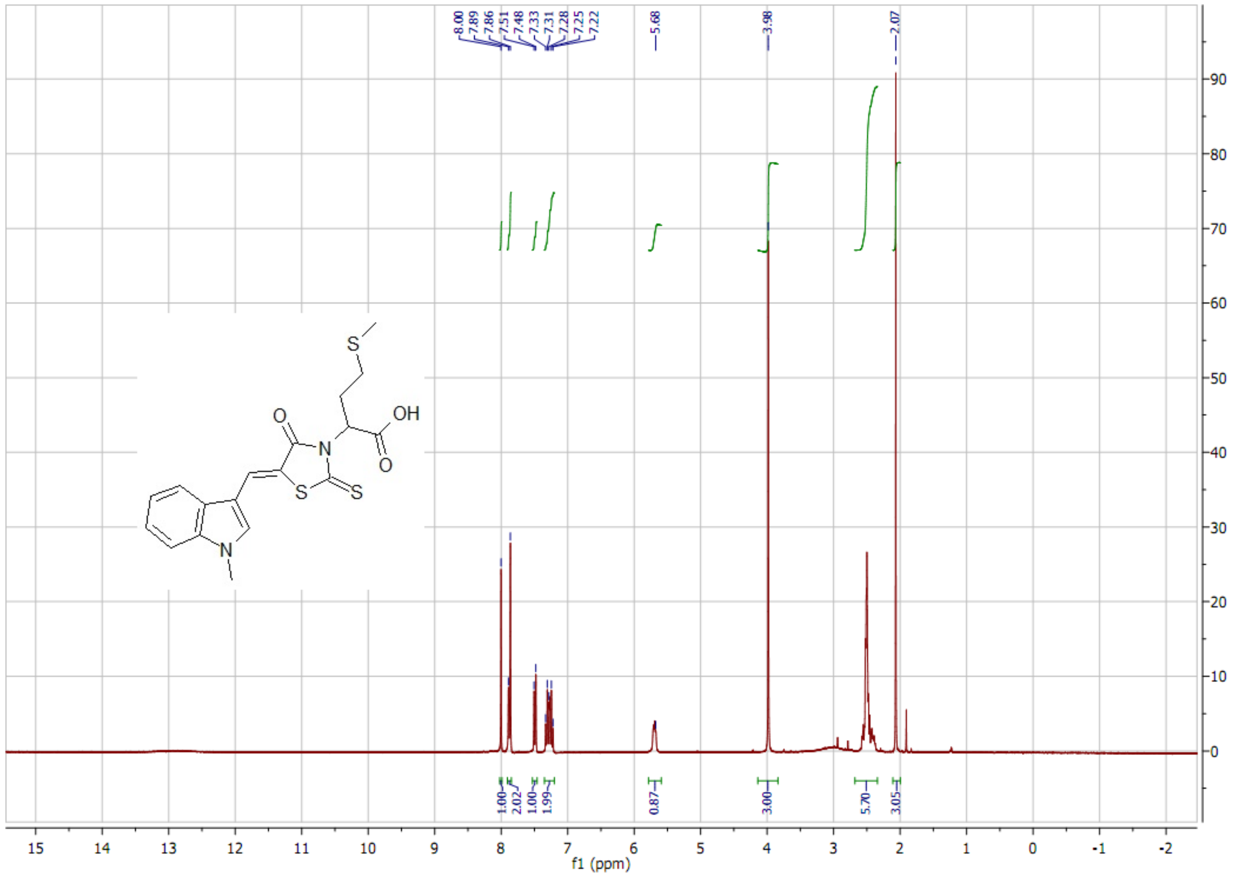

Figure S48. ^{1}HNMR spectrum (300 MHz, DMSO-d_6) of compound **5d**.

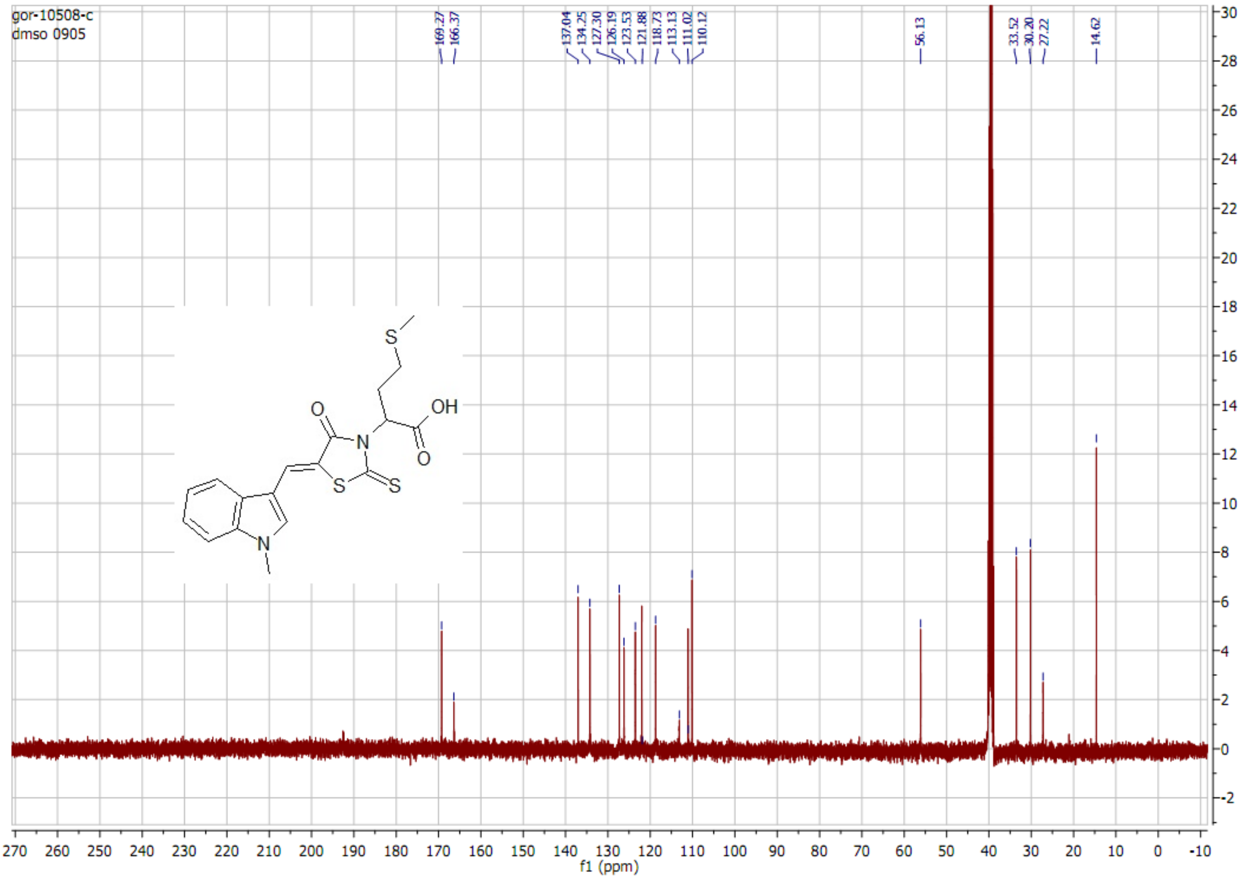

Figure S49. ^{13}C NMR spectrum (101 MHz, DMSO-d_6) of compound **5d.**

Sample Name: GOR-10508

-.o.-Syntez Purity Report -.o.-

System: Agilent 1200 LC/MSD SL
Separation column: Rapid Resolutionn HT Cartige 4.6x30mm, 1.8-Micron, Zorbx SB-C18

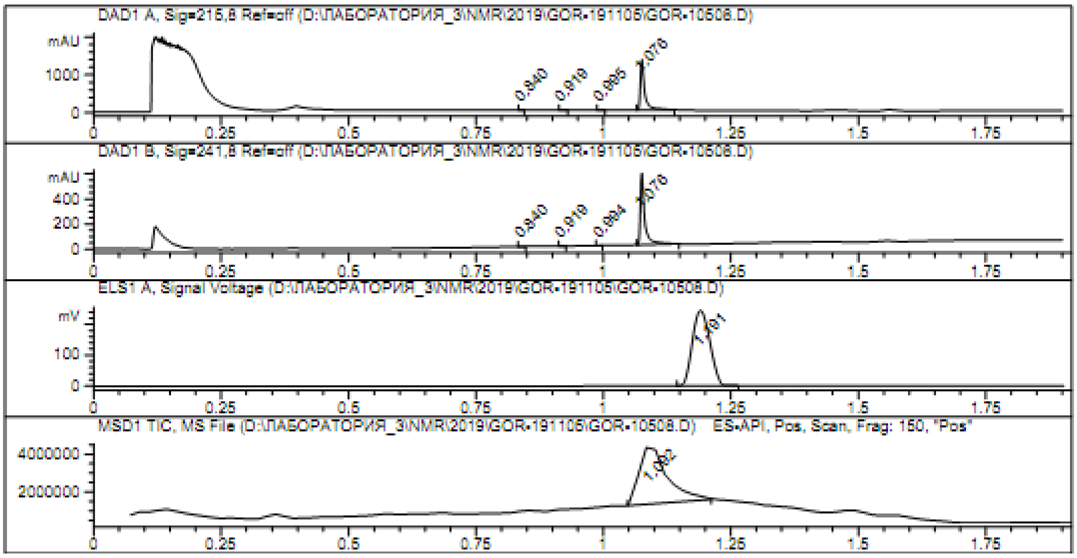

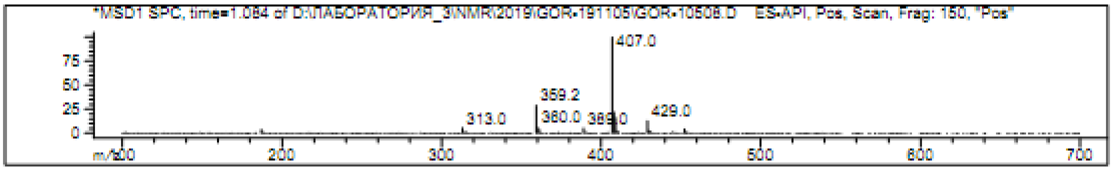

#	Signal	R.Time	Area %
1	DAD1 A, Sig=215,8 Ref=off	0.840	0.092
2		0.919	0.281
3		0.995	0.246
4		1.076	99.381

#	Signal	R.Time	Area %
1	DAD1 B, Sig=241,8 Ref=off	0.840	0.088
2		0.919	0.309
3		0.994	0.220
4		1.076	99.383

#	Signal	R.Time	Area %
1	ELS1 A, Signal Voltage	1.191	100.000

#	Signal	R.Time	Area %
1	MSD1 TIC, MS File	1.092	100.000

Inj. Data 05.11.2019

Figure S50. MS (ESI) spectrum of compound **5d.**

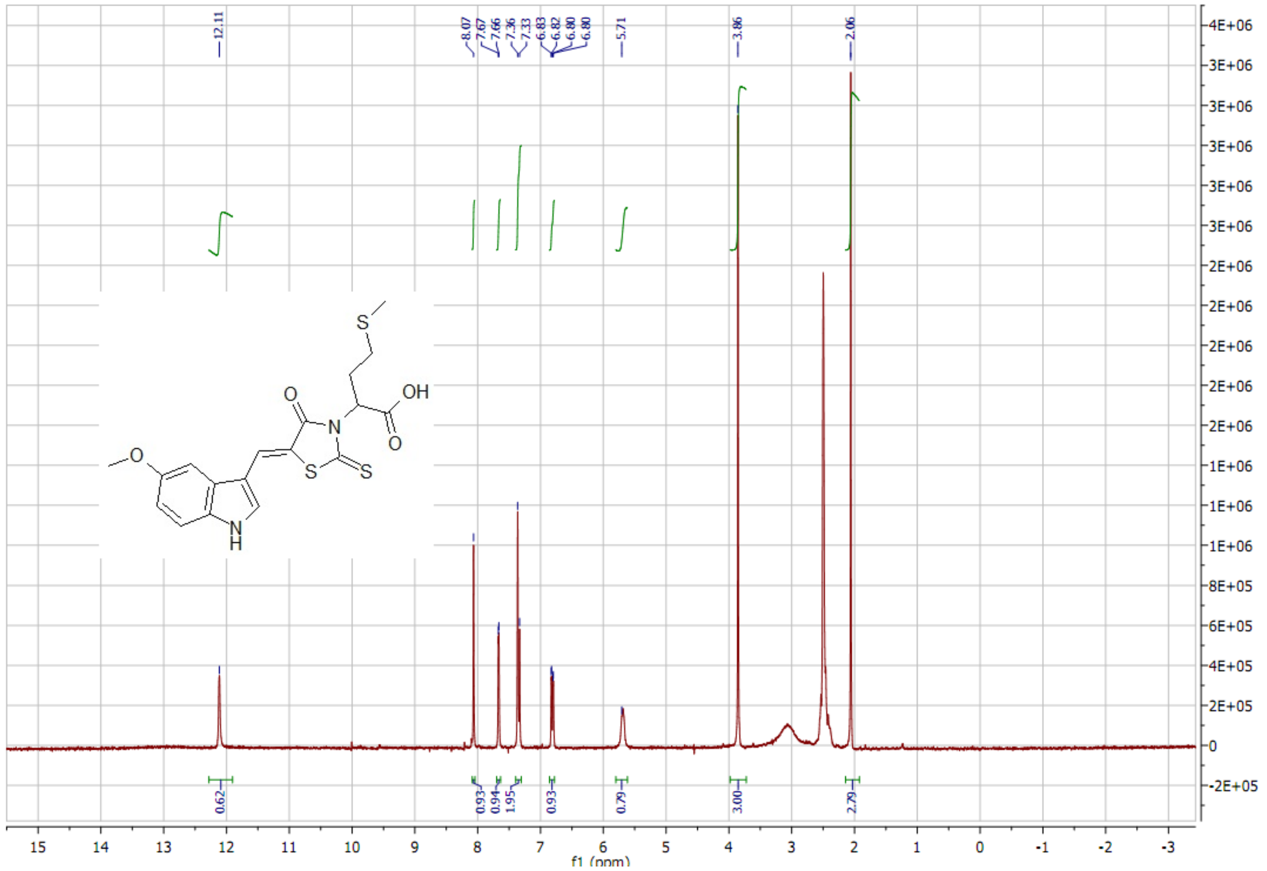

Figure S51. ^{1}HNMR spectrum (300 MHz, DMSO-d_6) of compound **5e**.

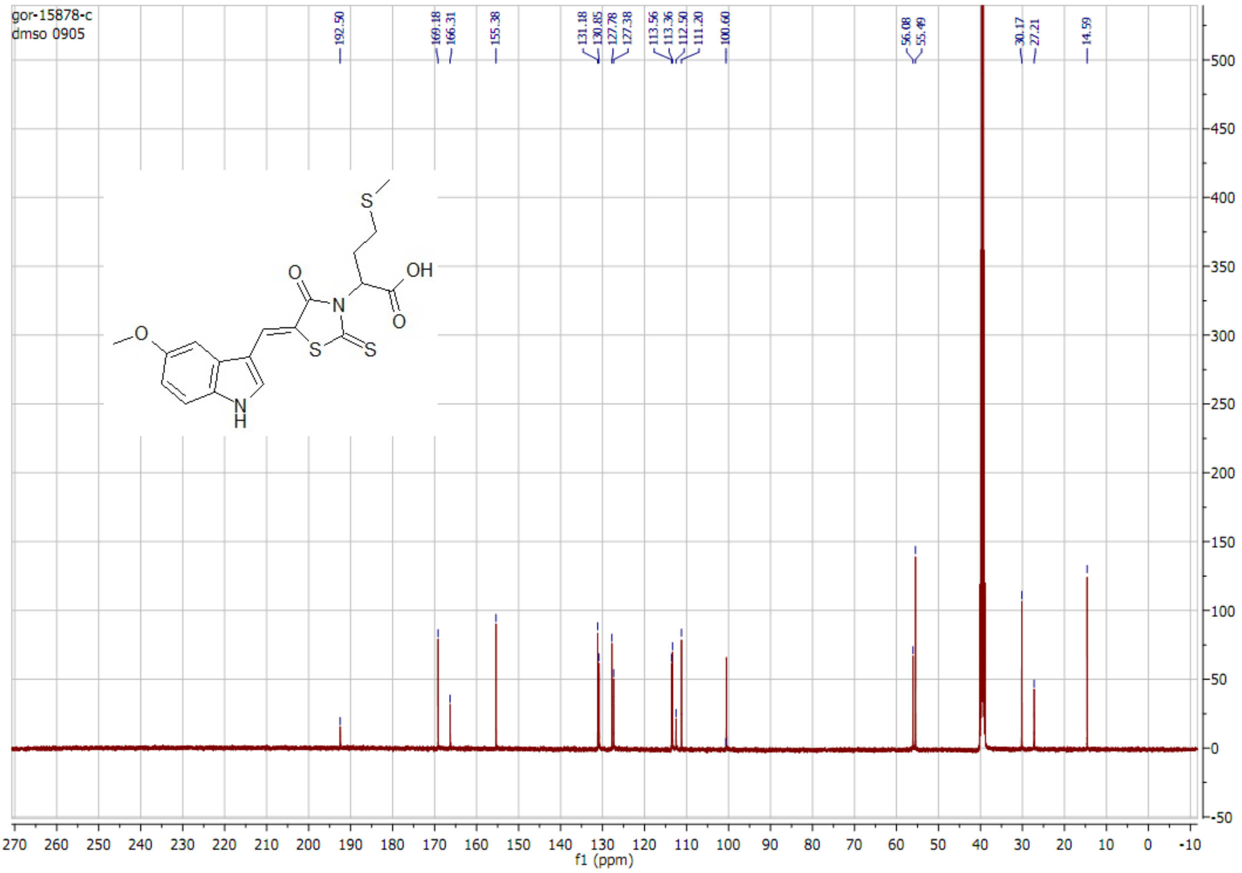

Figure S52. ^{13}C NMR spectrum (101 MHz, DMSO-d_6) of compound **5e.**

Sample Name: GOR-15878

-.o.-Syntez Purity Report -.o.-

System: Agilent 1200 LC/MSD SL
Separation column: Rapid Resolutionn HT Cartige 4.6x30mm, 1.8-Micron, Zorbx SB-C18

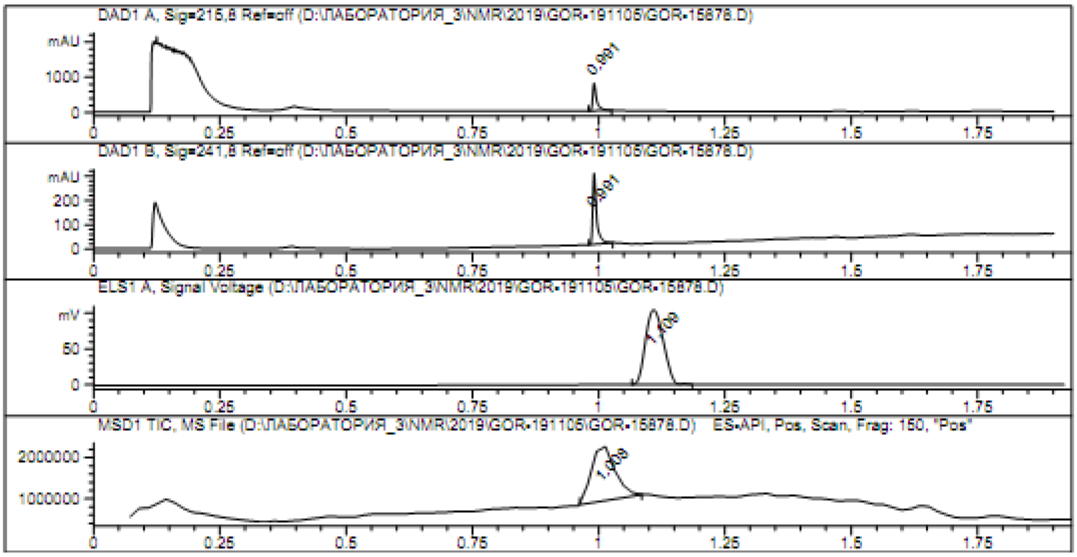

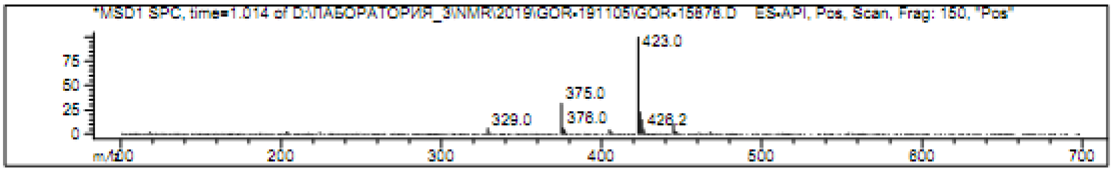

#	Signal	R.Time	Area %
1	DAD1 A, Sig=215,8 Ref=off	0.991	100.000

#	Signal	R.Time	Area %
1	DAD1 B, Sig=241,8 Ref=off	0.991	100.000

#	Signal	R.Time	Area %
1	ELS1 A, Signal Voltage	1.109	100.000

#	Signal	R.Time	Area %
1	MSD1 TIC, MS File	1.008	100.000

Inj. Data 05.11.2019

Figure S53. MS (ESI) spectrum of compound **5e.**

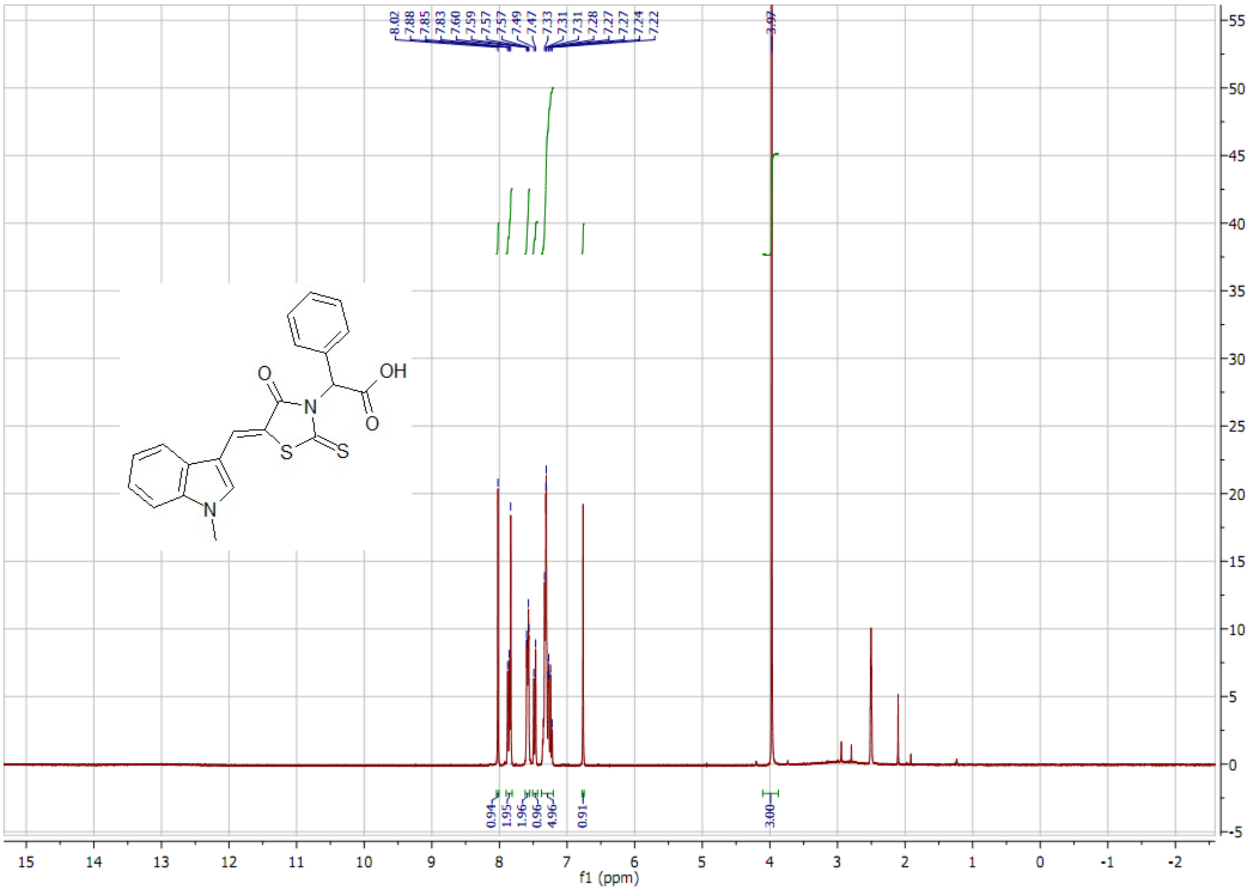

Figure S54. ^{1}HNMR spectrum (300 MHz, DMSO-d_6) of compound **5f**.

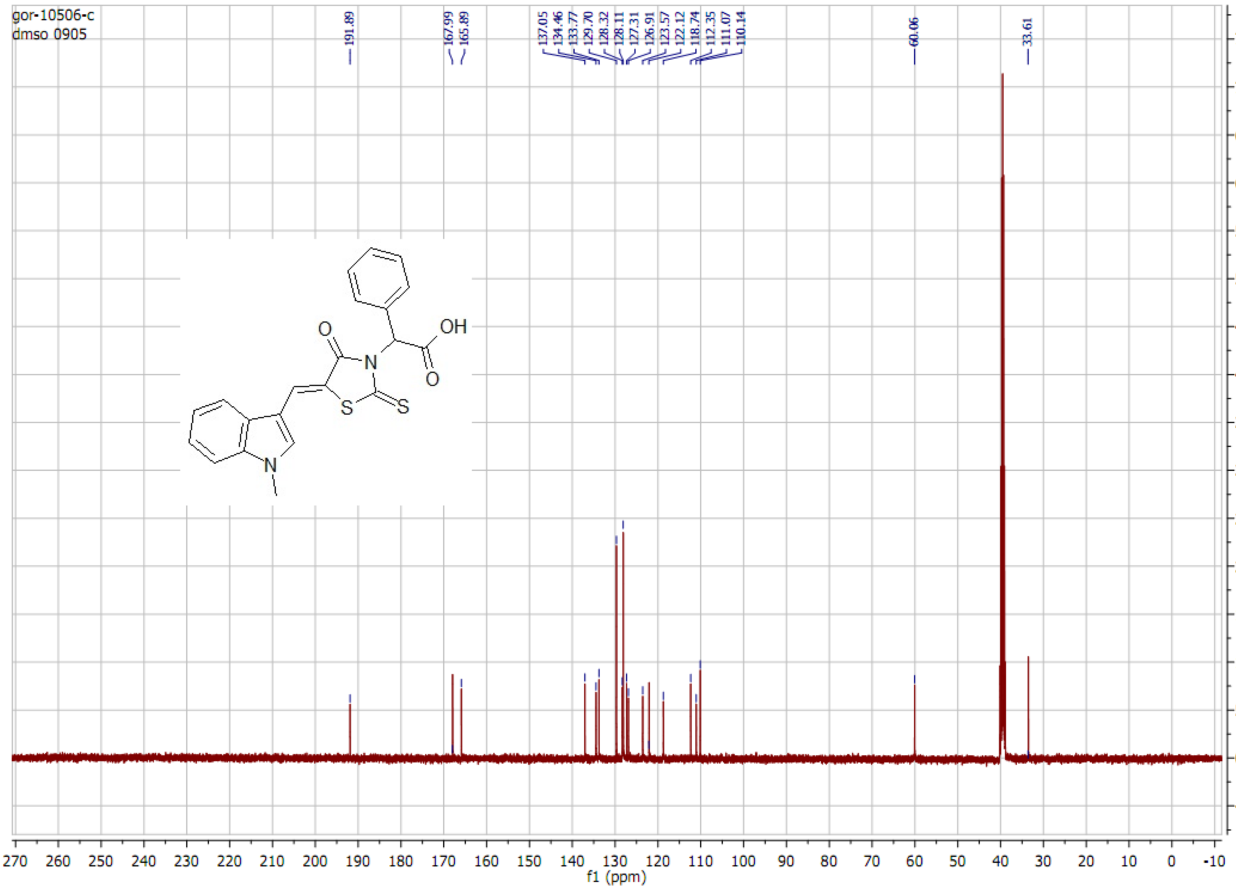

Figure S55. ^{13}C NMR spectrum (101 MHz, DMSO-d_6) of compound **5f.**

Sample Name: GOR-10506

-.o.-Syntez Purity Report -.o.-

System: Agilent 1200 LC/MSD SL
Separation column: Rapid Resolutionn HT Cartige 4.6x30mm, 1.8-Micron, Zorbx SB-C18

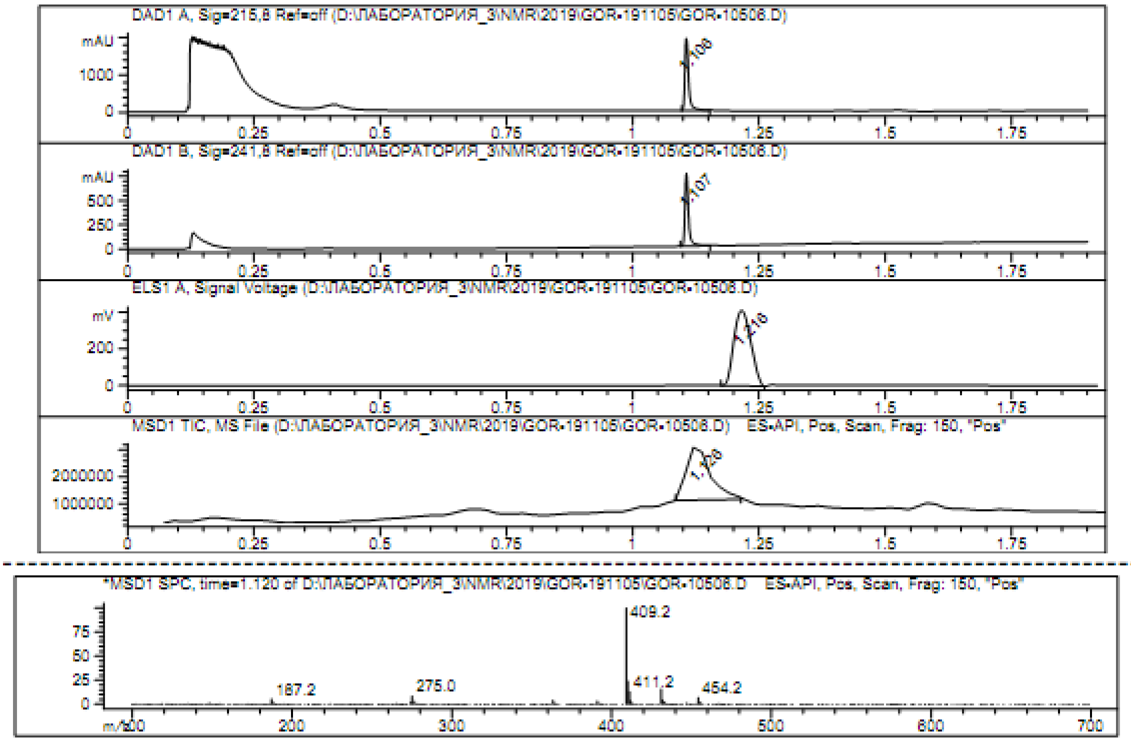

#	Signal	R.Time	Area %
1	DAD1 A, Sig=215,8 Ref=off	1.106	100.000

#	Signal	R.Time	Area %
1	DAD1 B, Sig=241,8 Ref=off	1.107	100.000

#	Signal	R.Time	Area %
1	ELS1 A, Signal Voltage	1.216	100.000

#	Signal	R.Time	Area %
1	MSD1 TIC, MS File	1.126	100.000

Inj. Data 06.11.2019

Figure S56. MS (ESI) spectrum of compound **5f.**

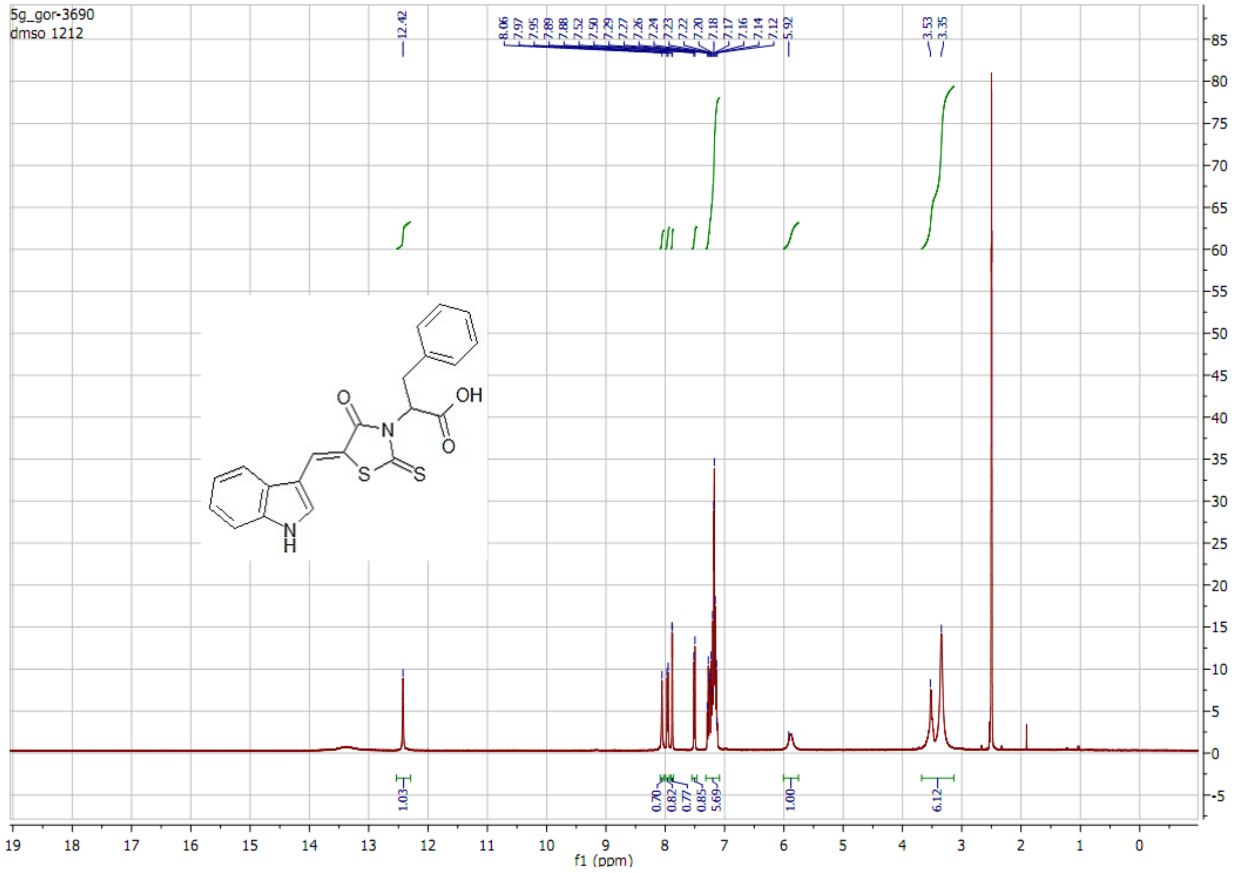

Figure S57. ^{1}HNMR spectrum (400 MHz, DMSO-d_6) of compound **5g**.

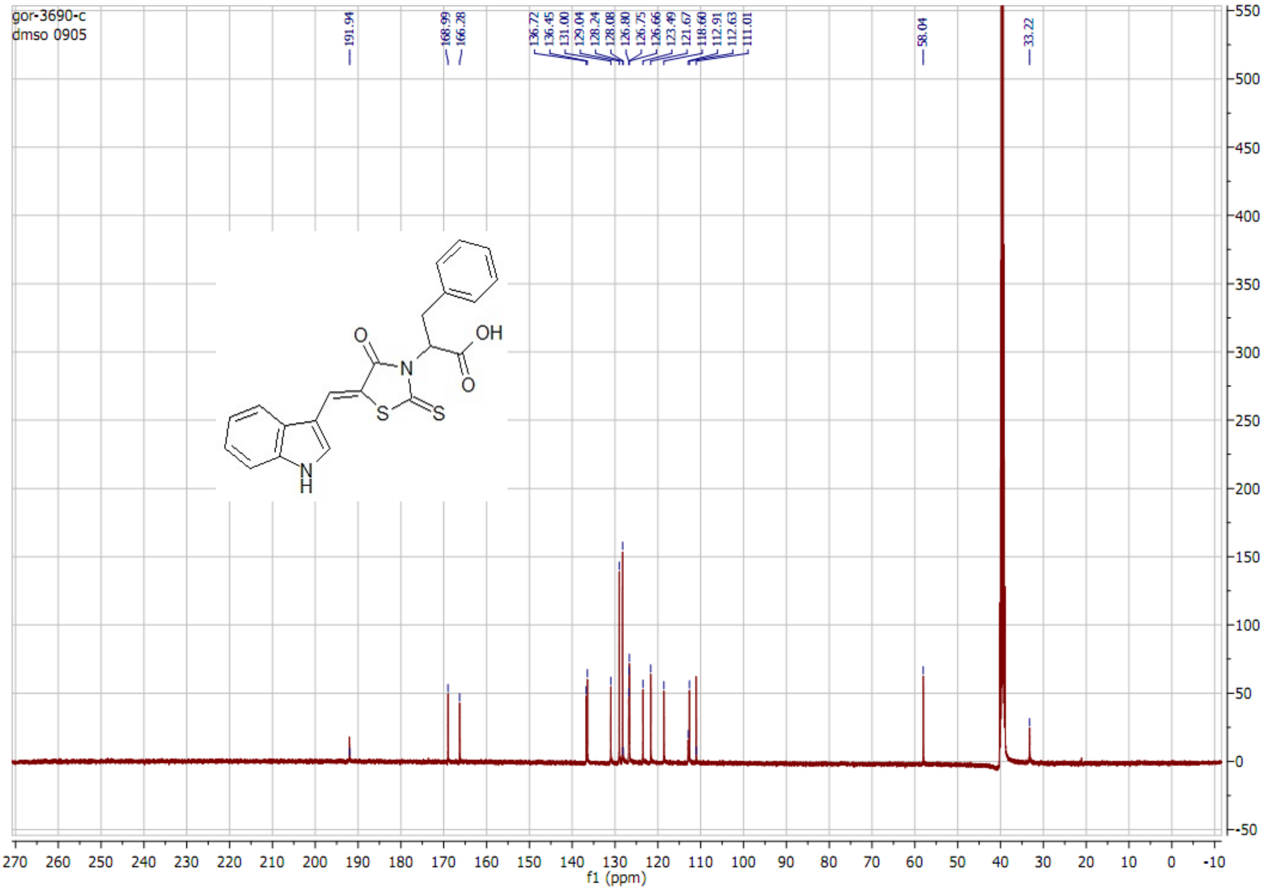

Figure S58. ^{13}C NMR spectrum (101 MHz, DMSO-d_6) of compound **5g.**

Sample Name: GOR-3490

-.o.-Syntez Purity Report -.o.-

System: Agilent 1200 LC/MSD SL
Separation column: Rapid Resolutionn HT Cartige 4.6x30mm, 1.8-Micron, Zorbx SB-C18

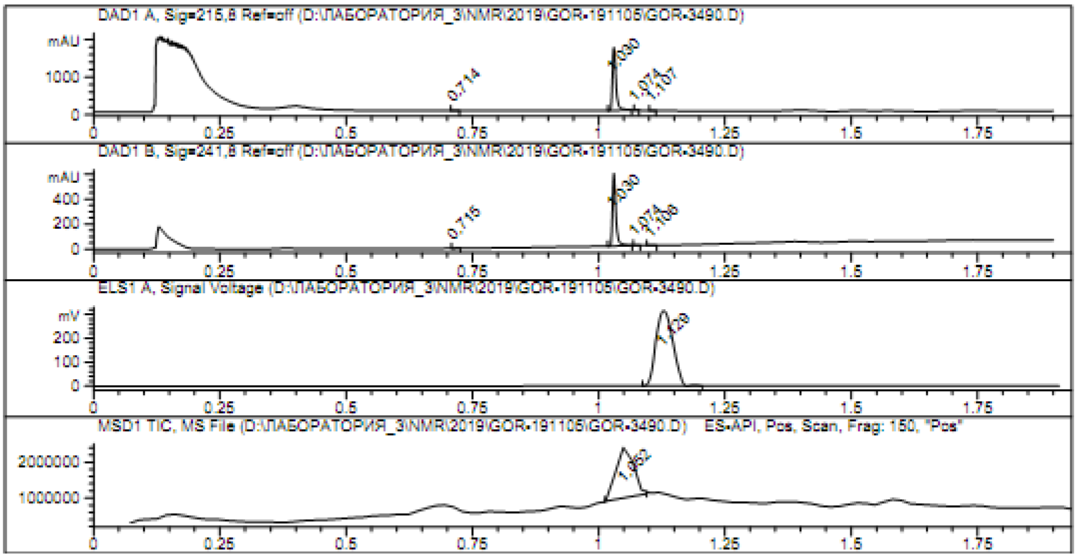

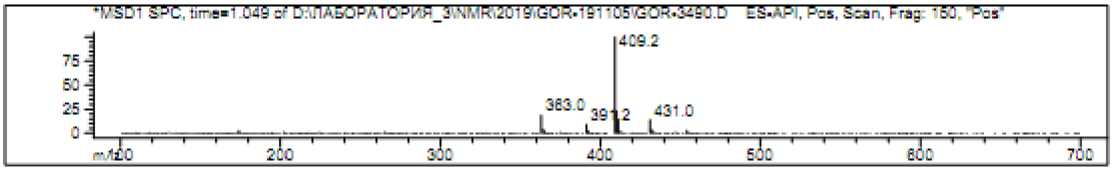

#	Signal	R.Time	Area %
1	DAD1 A, Sig=215,8 Ref=off	0.714	0.604
2		1.030	98.747
3		1.074	0.358
4		1.107	0.291

#	Signal	R.Time	Area %
1	DAD1 B, Sig=241,8 Ref=off	0.715	0.925
2		1.030	98.497
3		1.074	0.230
4		1.106	0.349

#	Signal	R.Time	Area %
1	ELS1 A, Signal Voltage	1.129	100.000

#	Signal	R.Time	Area %
1	MSD1 TIC, MS File	1.052	100.000

Inj. Data 06.11.2019

Figure S59. MS (ESI) spectrum of compound **5g.**

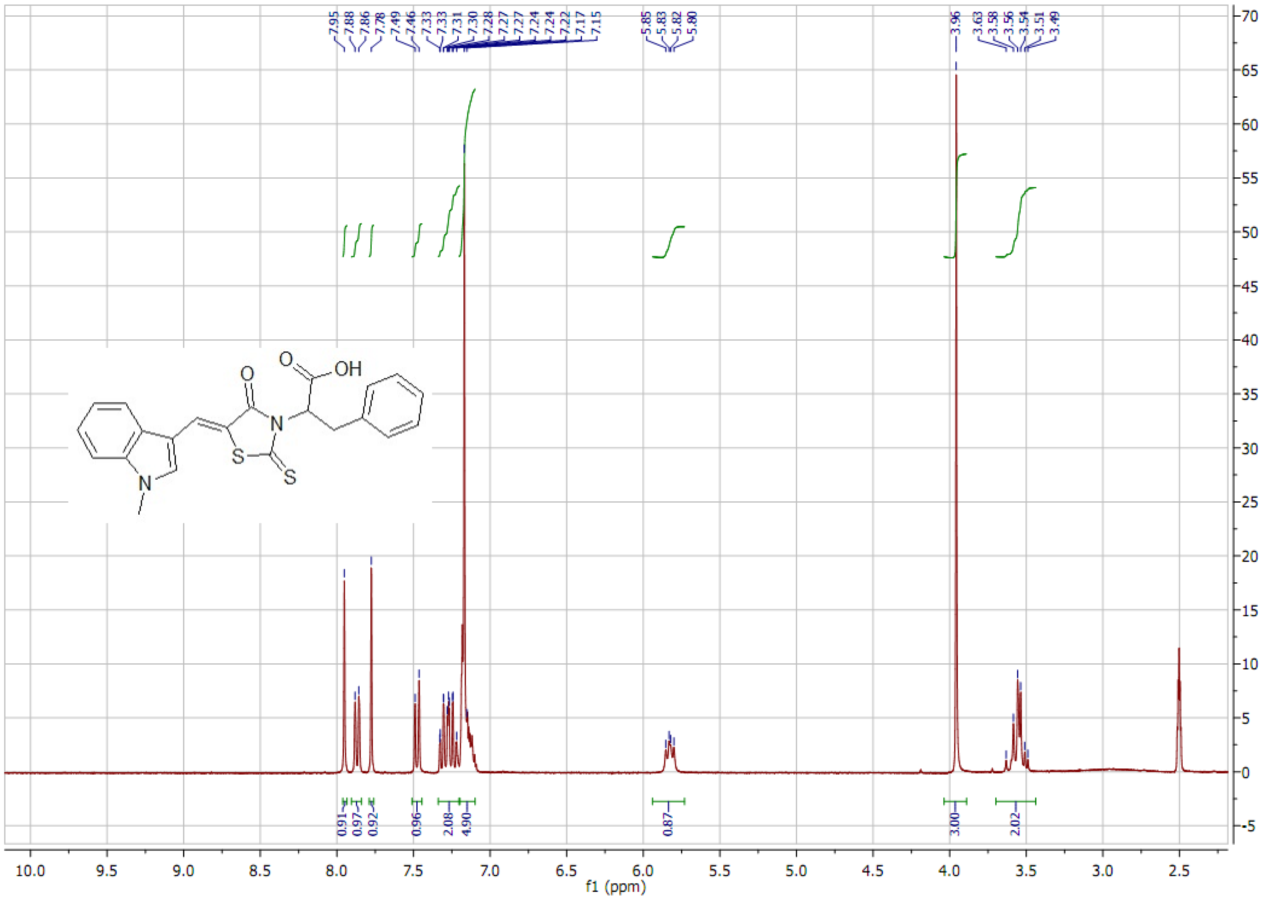

Figure S60. ^{1}HNMR spectrum (300 MHz, DMSO-d_6) of compound **5h**.

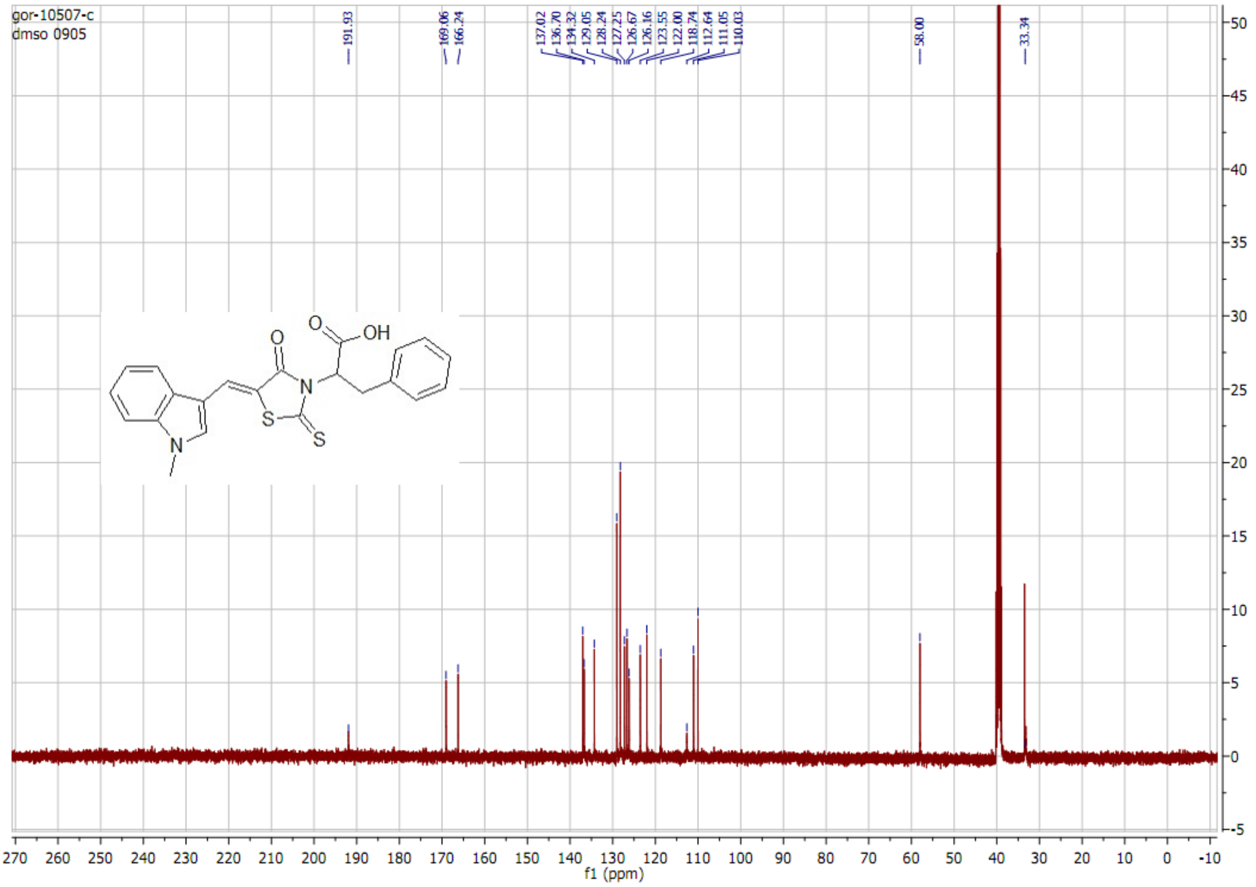

Figure S61. ^{13}C NMR spectrum (101 MHz, DMSO-d_6) of compound **5h.**

Sample Name: GOR-10507

-.o.-Syntez Purity Report -.o.-

System: Agilent 1200 LC/MSD SL
Separation column: Rapid Resolutionn HT Cartige 4.6x30mm, 1.8-Micron, Zorbx SB-C18

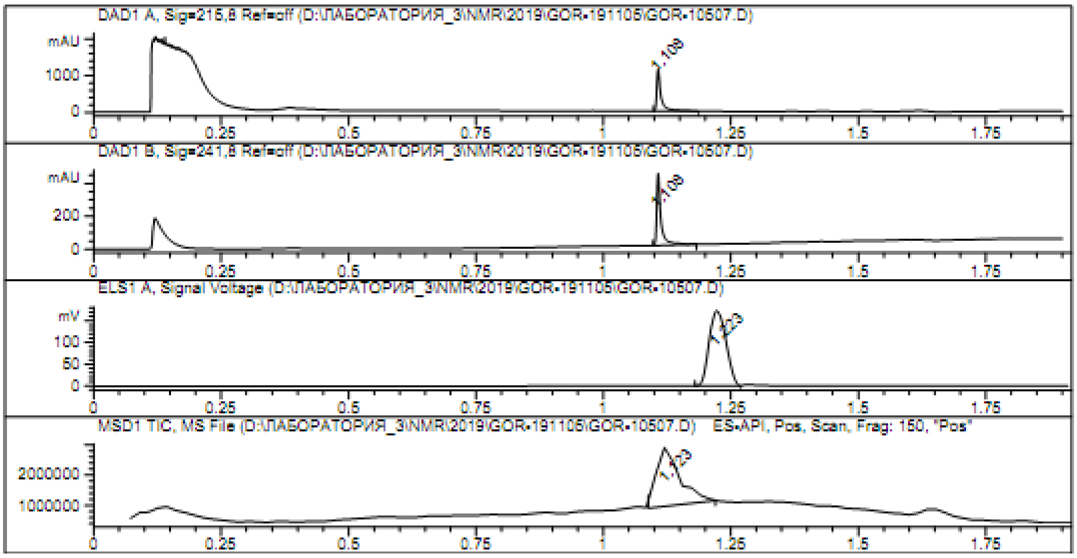

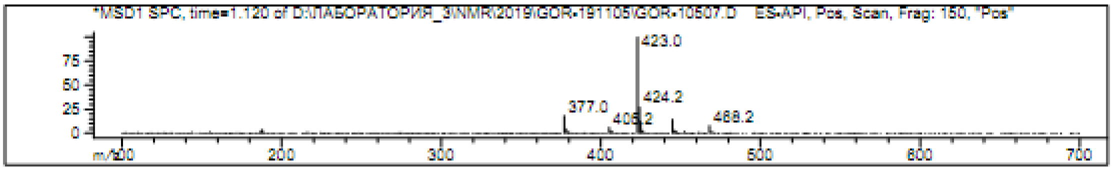

#	Signal	R.Time	Area %
1	DAD1 A, Sig=215,8 Ref=off	1.108	100.000

#	Signal	R.Time	Area %
1	DAD1 B, Sig=241,8 Ref=off	1.108	100.000

#	Signal	R.Time	Area %
1	ELS1 A, Signal Voltage	1.223	100.000

#	Signal	R.Time	Area %
1	MSD1 TIC, MS File	1.123	100.000

Inj. Data 05.11.2019

Figure S62. MS (ESI) spectrum of compound **5h.**

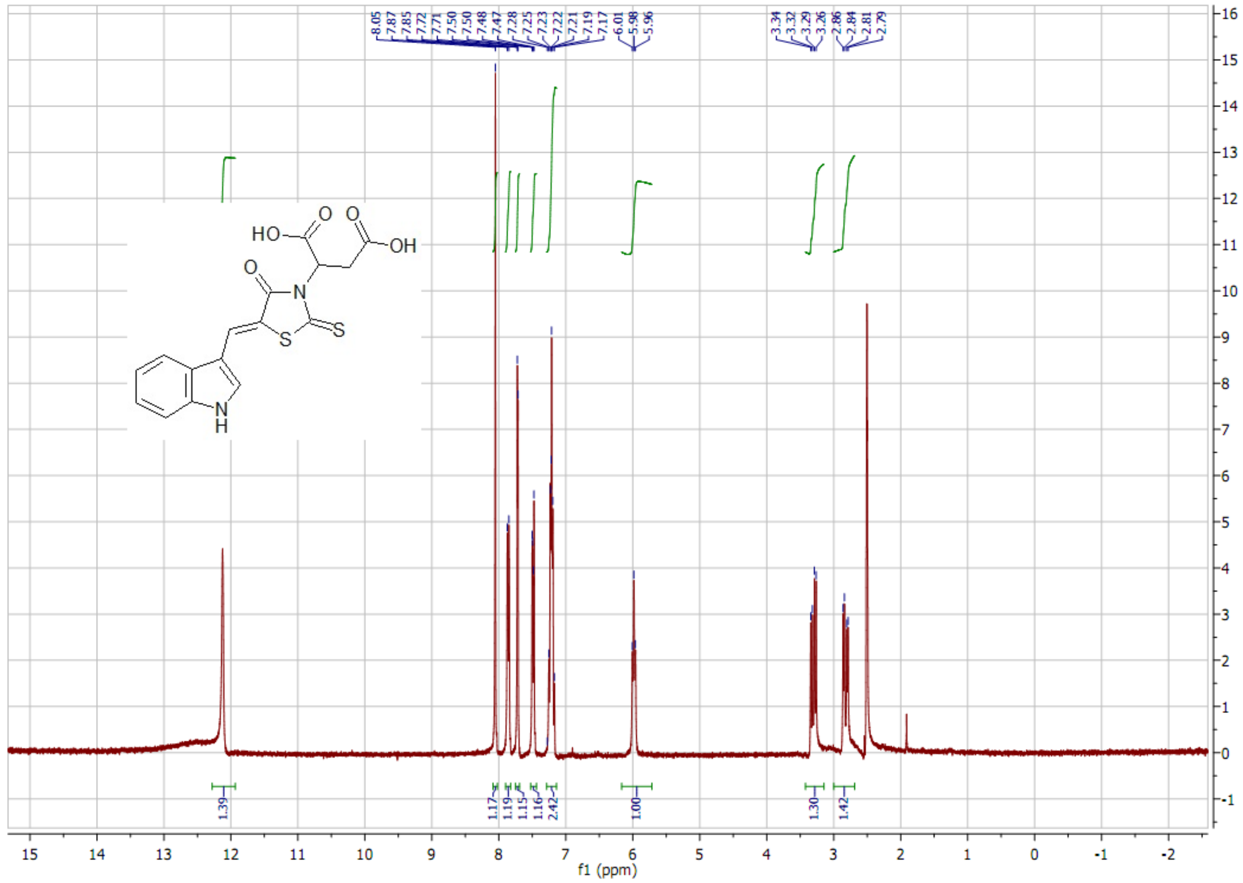

Figure S63. ^{1}HNMR spectrum (300 MHz, DMSO-d_6) of compound **5i**.

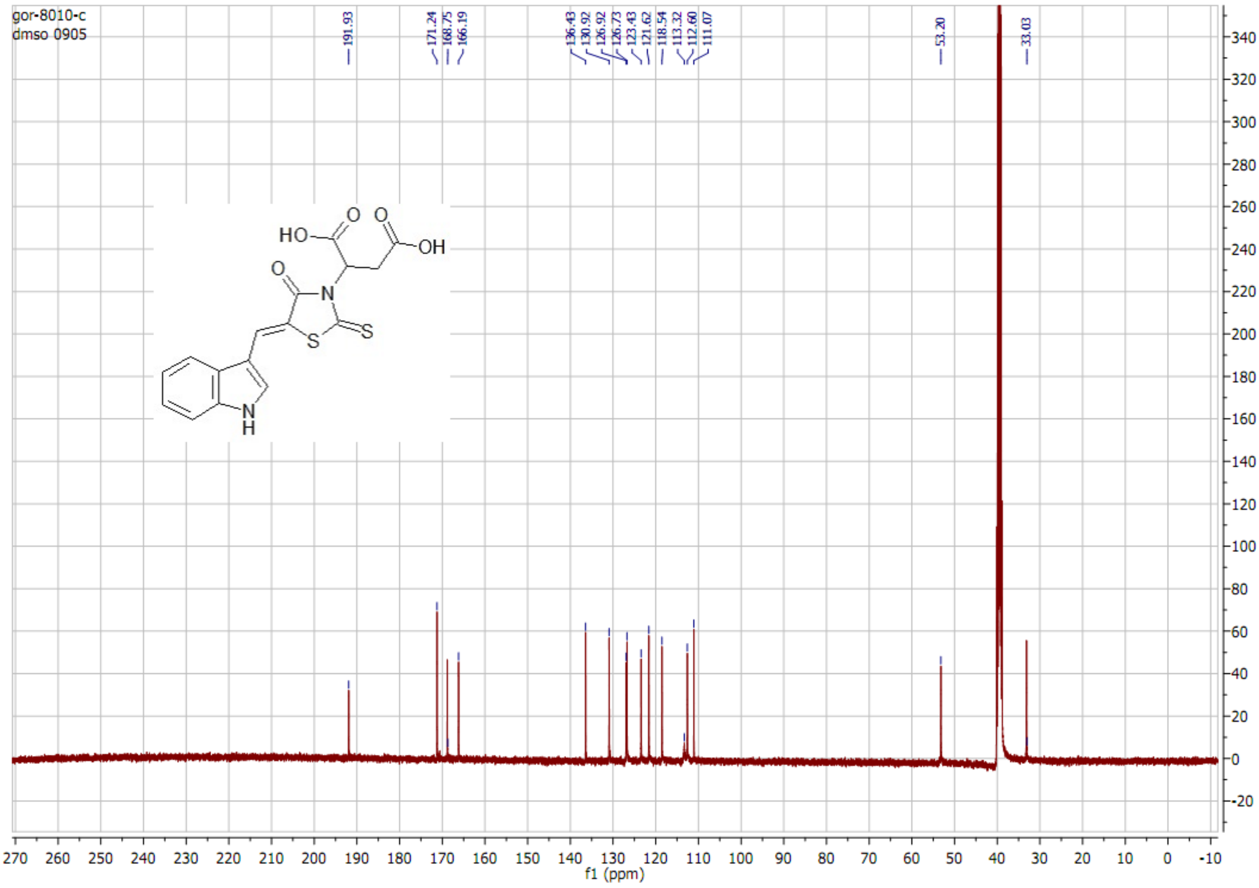

Figure S64. ^{13}C NMR spectrum (101 MHz, DMSO-d_6) of compound **5i.**

Sample Name: GOR-8010

-.o.-Syntez Purity Report -.o.-

System: Agilent 1200 LC/MSD SL
Separation column: Rapid Resolutionn HT Cartige 4.6x30mm, 1.8-Micron, Zorbx SB-C18

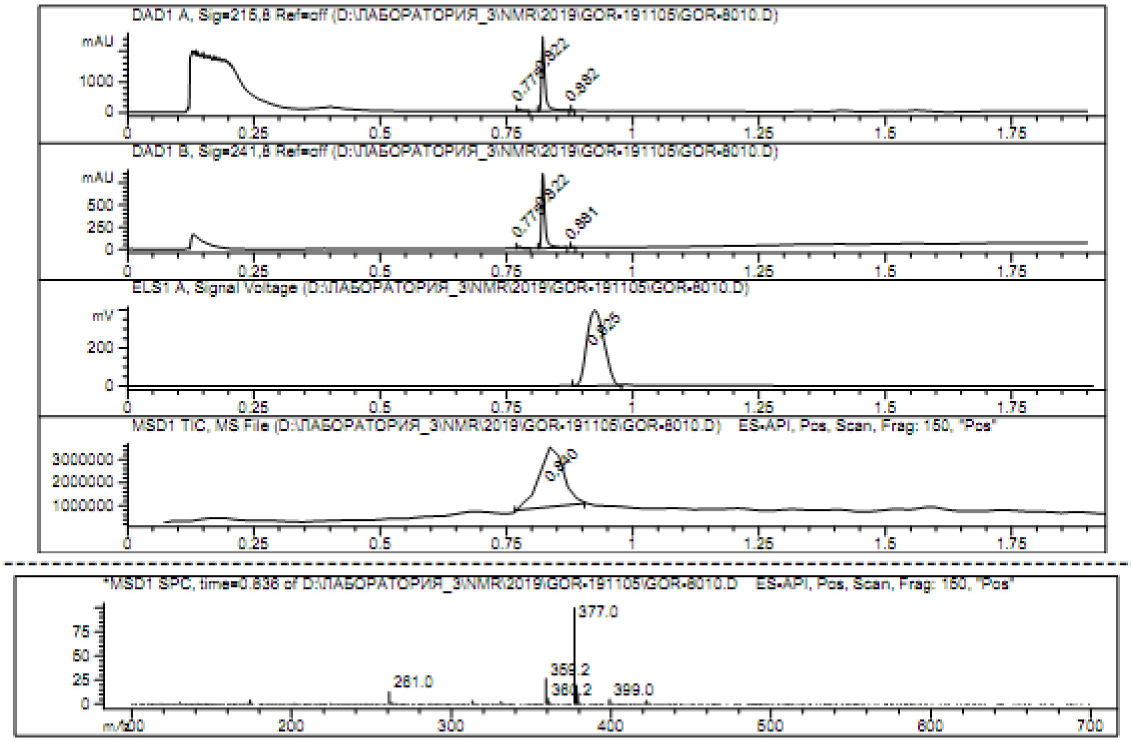

#	Signal	R.Time	Area %
1	DAD1 A, Sig=215,8 Ref=off	0.775	3.142
2		0.822	96.774
3		0.882	0.085

#	Signal	R.Time	Area %
1	DAD1 B, Sig=241,8 Ref=off	0.775	2.670
2		0.822	97.100
3		0.881	0.230

#	Signal	R.Time	Area %
1	ELS1 A, Signal Voltage	0.925	100.000

#	Signal	R.Time	Area %
1	MSD1 TIC, MS File	0.840	100.000

Inj. Data 06.11.2019

Figure S65. MS (ESI) spectrum of compound **5i.**

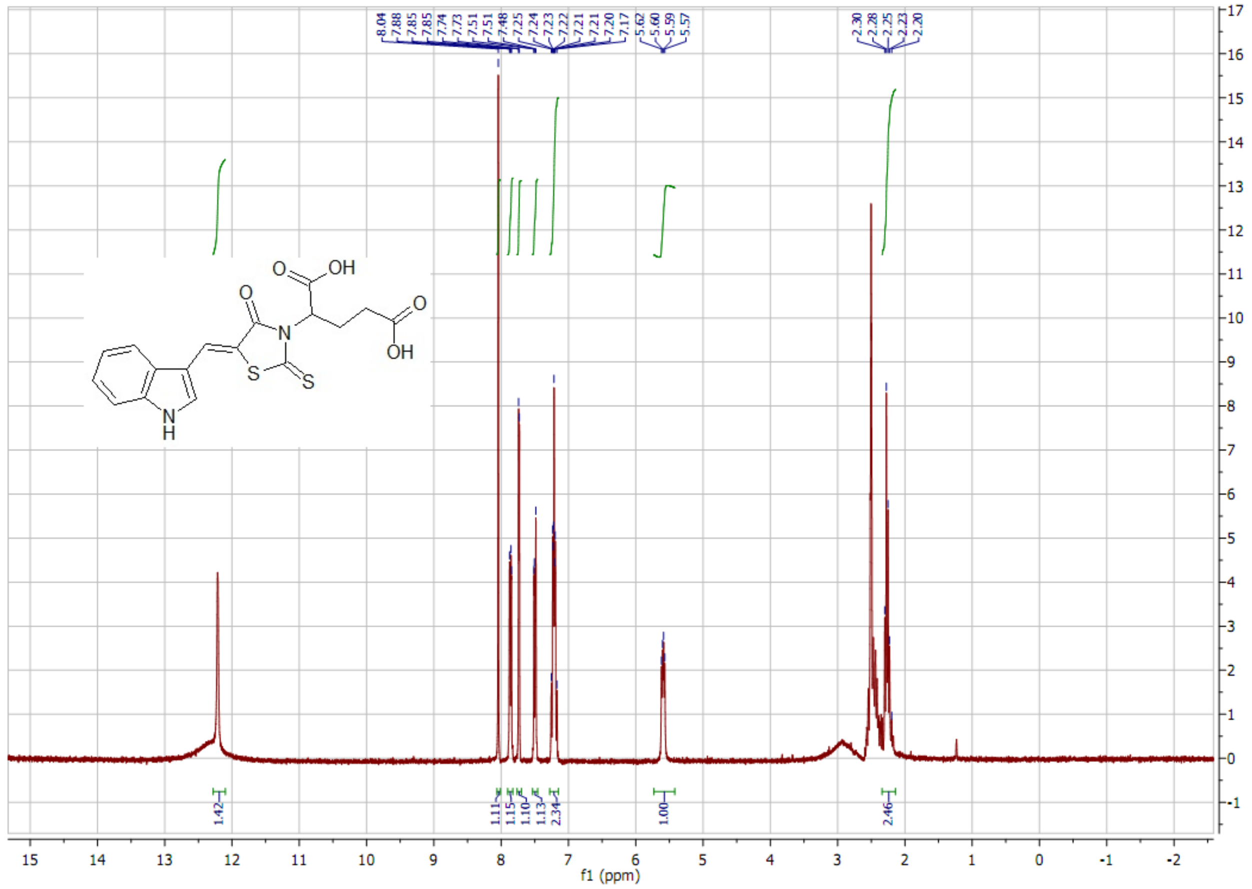

Figure S66. ^{1}HNMR spectrum (300 MHz, DMSO-d_6) of compound **5j**.

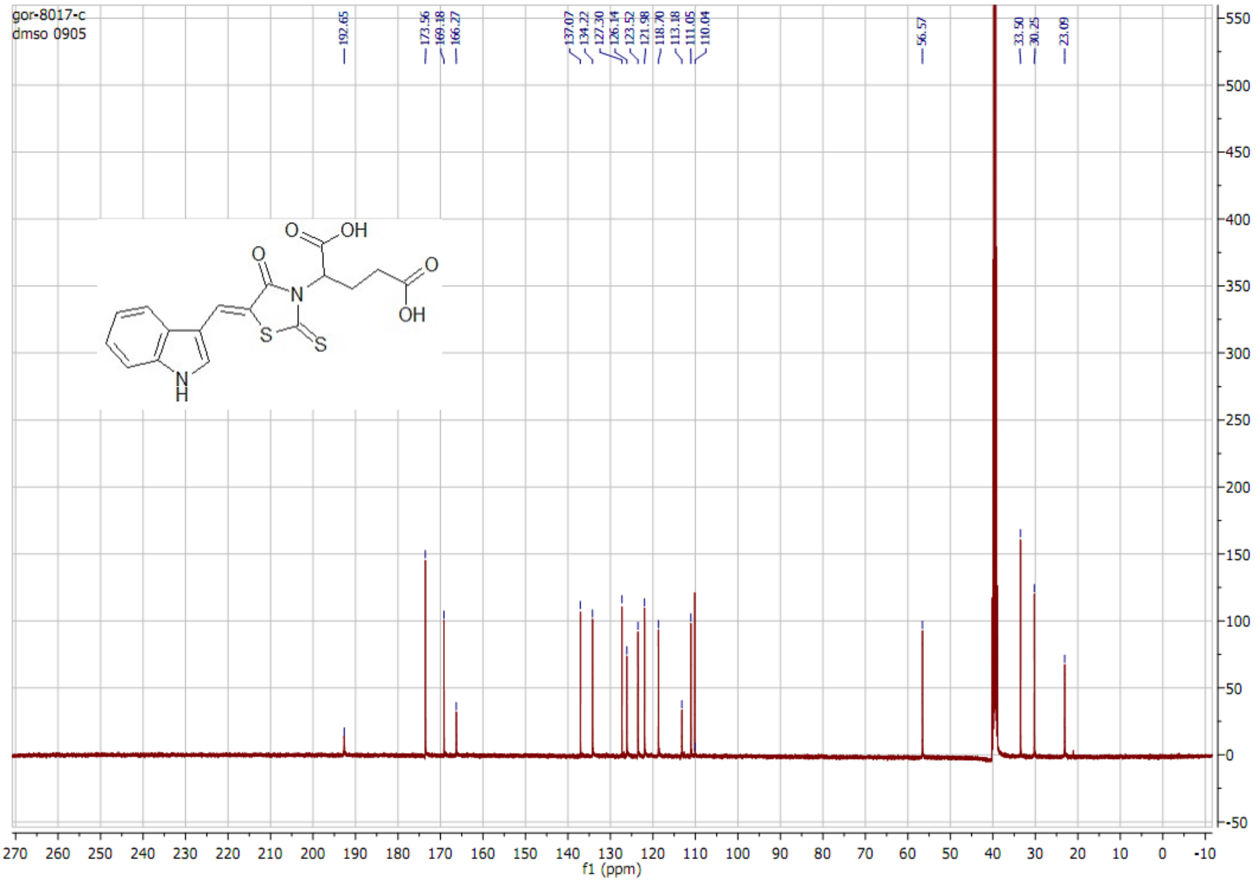

Figure S67. ^{13}C NMR spectrum (101 MHz, DMSO-d_6) of compound **5j.**

Sample Name: GOR-8017

-.o.-Syntez Purity Report -.o.-

System: Agilent 1200 LC/MSD SL
Separation column: Rapid Resolutionn HT Cartige 4.6x30mm, 1.8-Micron, Zorbx SB-C18

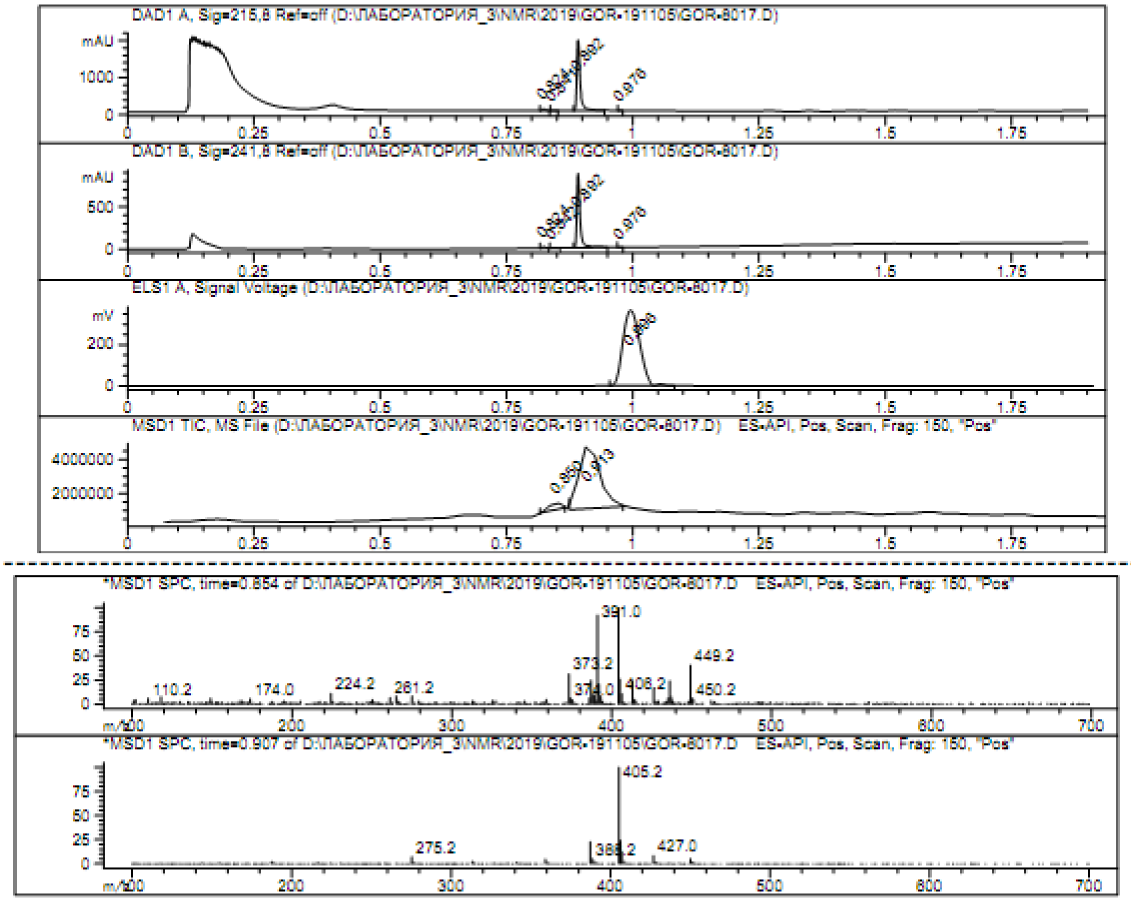

#	Signal	R.Time	Area %
1	DAD1 A, Sig=215,8 Ref=off	0.824	2.024
2		0.841	0.377
3		0.892	97.395
4		0.976	0.204

#	Signal	R.Time	Area %
1	DAD1 B, Sig=241,8 Ref=off	0.824	1.643
2		0.842	0.513
3		0.892	97.629
4		0.976	0.215

#	Signal	R.Time	Area %
1	ELS1 A, Signal Voltage	0.996	100.000

#	Signal	R.Time	Area %
1	MSD1 TIC, MS File	0.850	6.581
2		0.913	93.419

Inj. Data 06.11.2019

Figure S68. MS (ESI) spectrum of compound **5j.**

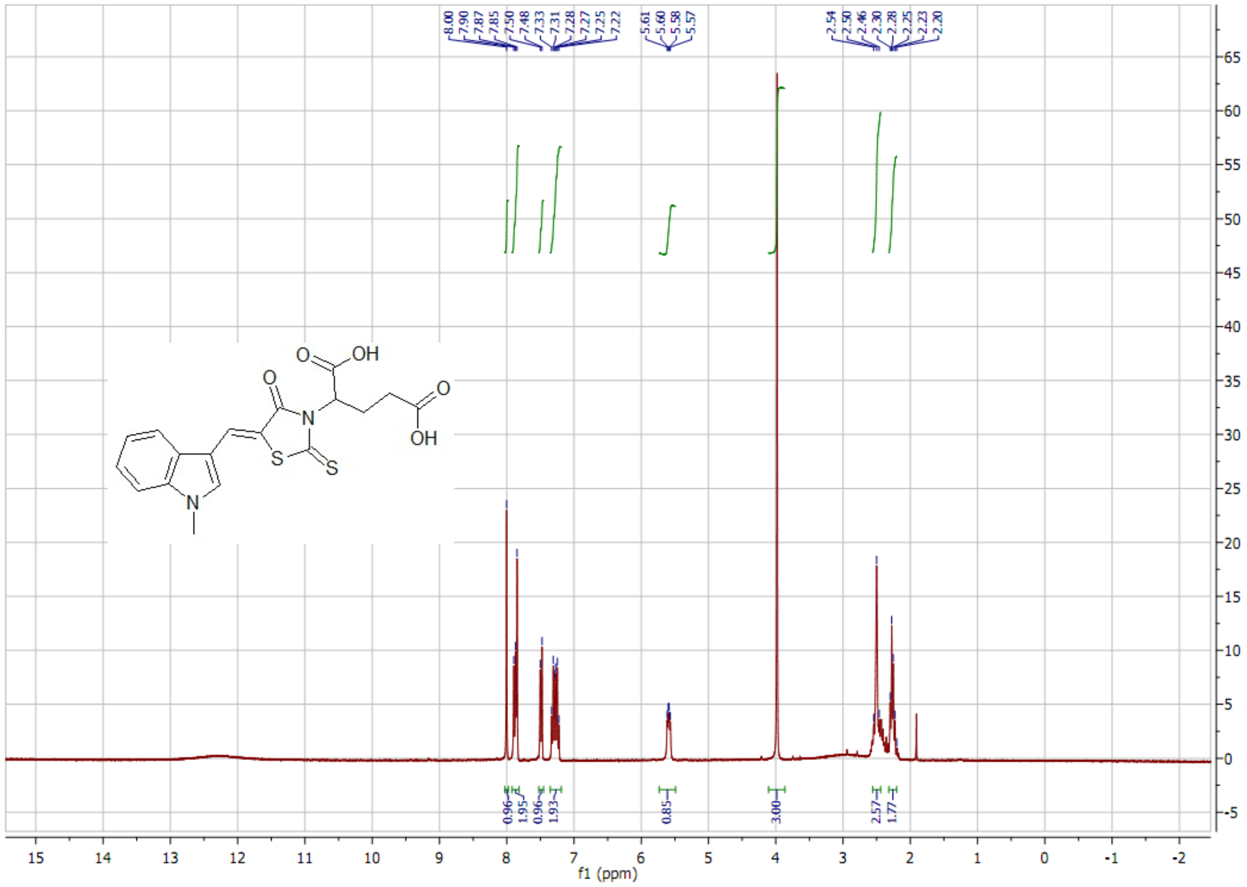

Figure S69. ^{1}HNMR spectrum (300 MHz, DMSO-d$_6$) of compound **5k**.

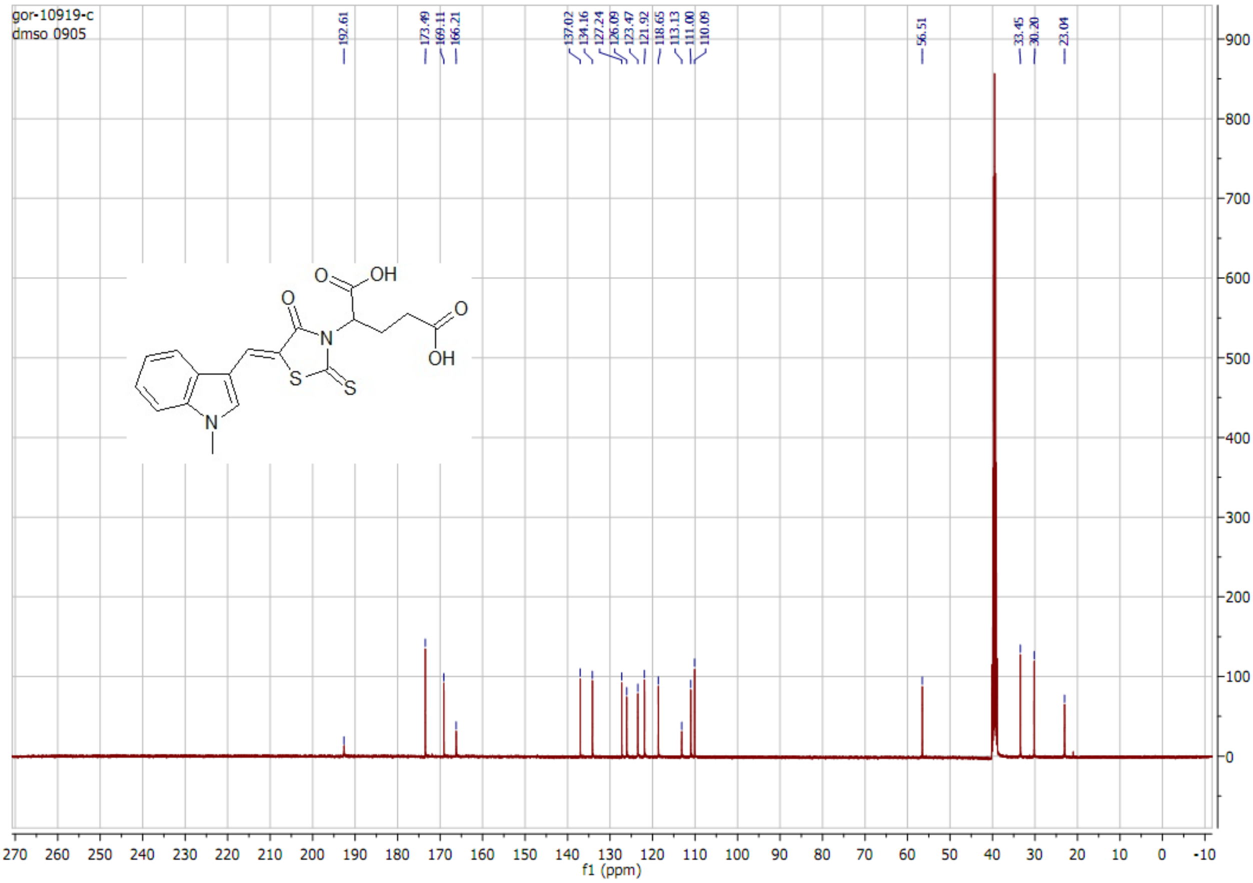

Figure S70. ^{13}C NMR spectrum (101 MHz, DMSO-d$_6$) of compound **5k.**

[illegible]

-.o.-Syntez Purity Report -.o.-

System: Agilent 1200 LC/MSD SL
Separation column: Rapid Resolutionn HT Cartige 4.6x30mm, 1.8-Micron, Zorbx SB-C18

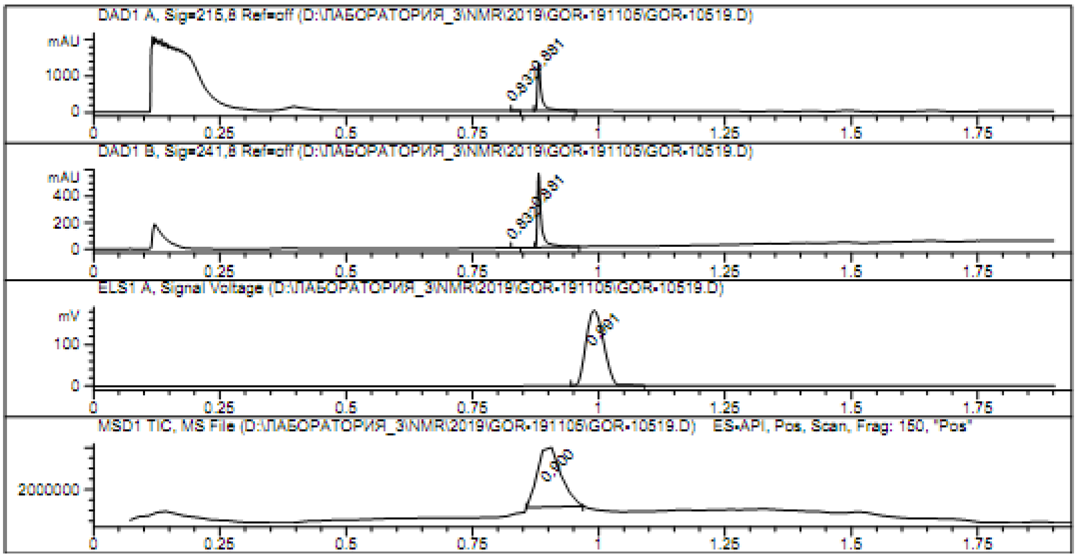

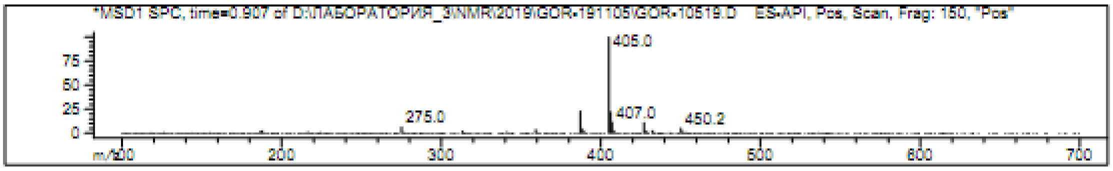

#	Signal	R.Time	Area %
1	DAD1 A, Sig=215,8 Ref=off	0.832	0.394
2		0.881	99.606

#	Signal	R.Time	Area %
1	DAD1 B, Sig=241,8 Ref=off	0.832	0.785
2		0.881	99.215

#	Signal	R.Time	Area %
1	ELS1 A, Signal Voltage	0.991	100.000

#	Signal	R.Time	Area %
1	MSD1 TIC, MS File	0.900	100.000

Inj. Data 05.11.2019

Figure S71. MS (ESI) spectrum of compound **5k.**
